# Temporal Analysis of Pharmaceuticals as Emerging Contaminants in Surface Water and Wastewater Samples: A Case Study

**DOI:** 10.3390/jox14030048

**Published:** 2024-07-03

**Authors:** Paula Paíga, Luísa Correia-Sá, Manuela Correia, Sónia Figueiredo, Joana Vieira, Sandra Jorge, Jaime Gabriel Silva, Cristina Delerue-Matos

**Affiliations:** 1 REQUIMTE/LAQV, Instituto Superior de Engenharia do Porto, Instituto Politécnico do Porto, Rua Dr. António Bernardino de Almeida, 431, 4249-015 Porto, Portugal; mlsrs@isep.ipp.pt (L.C.-S.); mmb@isep.ipp.pt (M.C.); saf@isep.ipp.pt (S.F.); 2 Águas do Centro Litoral, SA, Grupo Águas de Portugal, ETA da Boavista, Avenida Dr. Luís Albuquerque, 3030-410 Coimbra, Portugal; j.vieira@adp.pt (J.V.);; 3 Águas do Douro e Paiva, SA, Grupo Águas de Portugal, Rua de Vilar, 235 5°, 4050-626 Porto, Portugal; g.silva@adp.pt; 4 Departamento de Engenharia Civil, Instituto Superior de Engenharia do Porto, Instituto Politécnico do Porto, Rua Dr. António Bernardino de Almeida, 431, 4249-015 Porto, Portugal

**Keywords:** overtime analysis, pharmaceuticals, public health, surface and wastewater samples, liquid chromatography, mass spectrometry

## Abstract

Pharmaceuticals in the environment are a global concern, with studies in all continents highlighting their widespread occurrence and potential ecological impacts, revealing their presence, fate, and associated risks in aquatic ecosystems. Despite typically occurring at low concentrations (ranging from ng/L to µg/L), advancements in analytical methods and more sensitive equipment have enabled the detection of a higher number of pharmaceuticals. In this study, surface and wastewater samples were extracted using solid phase extraction and analyzed using ultra-high-performance liquid chromatography with tandem mass spectrometry. Among the therapeutic classes investigated, nonsteroidal anti-inflammatory drugs/analgesics, antibiotics, and psychiatric drugs showed a higher number of detected pharmaceuticals. Concentrations ranged from below method detection limit (<MDL) to 3.20 µg/L (caffeine) and <MDL to 639 µg/L (hydroxyibuprofen) in 2018, and from <MDL to 0.848 µg/L (diclofenac) and <MDL to 53.0 µg/L (caffeine) in 2019 for river water and wastewater samples. Temporal analysis showed an increase in the sum of pharmaceutical concentrations over the study years, highlighting the importance of monitoring pharmaceuticals in the environment and their potential accumulation over time.

## 1. Introduction

Environmental pollution continues to increase day after day in a proportion that is difficult to control or eliminate and has become a global transboundary problem [1]. Emerging contaminants are a group of compounds of high global concern due to their potential threat to humans, animals, and the environment. Pharmaceuticals (for human and veterinary use) have been recognized among these contaminants to monitor since they are continually released into the environment [2,3]. Five decades after the first published study [4] the number of publications by the scientific community continues to increase when pharmaceuticals and the environment are the keywords of the research topic.

Wastewater treatment plants (WWTPs) were built as an important stage in the purification of wastewater, primarily designed to remove organic matter, suspended solids, and nutrients [5]. However, WWTPs have emerged as the primary pathway for pharmaceuticals to enter the environment [6,7]. As WWTPs were not designed to remove pharmaceuticals, the treatments applied may not be fully effective in completely removing these contaminants, leading to their release into the aquatic environment.

Another important subject is the inclusion of metabolites and degradation products in the list of compounds to be monitored. The majority of the published works are focused on the parent compounds and less attention is given to metabolites or degradation products. Although there are many published papers (382,833) about “pharmaceuticals and environment”, only a few (359) address metabolites and degradation products. This is likely because they are usually more challenging to detect due to their lower concentrations and complex chemical structures [8].

Hence, conducting monitoring studies becomes crucial to (i) ascertain the presence and levels of pharmaceuticals in the environment, (ii) assess the impacts of human activities, (iii) identify the primary pathways of contamination, (iv) evaluate the potential risks to humans, animals, and the environment, (v) raise public awareness, and (vi) alert relevant authorities. Vigilance, regulation, and proactive measures must be emphasized to address the pressing concern of pollution. Additionally, it is important to explore the design of new effective purification methods for WWTPs, particularly in the removal of pharmaceutical compounds, namely combining biological treatment processes with physical-chemical ones to enhance their removal efficiency. Advanced oxidation processes (such as photocatalysis [9], Fenton or photo-Fenton or electro-Fenton [10], ozonation [11], ultrasonication [12], electrochemical oxidation [13], persulfate oxidation [14]), and membrane filtration [15] have shown promising results in reducing pharmaceutical concentrations in treated wastewater. These advanced treatment methods offer different approaches to removing pharmaceuticals from wastewater, each with its advantages and limitations. In 2023, Liu et al. [16] published a review article stating that there is a growing need to explore novel removal techniques for pharmaceuticals and presented the latest advancements while providing a concise overview of the types, features, and risks of pharmaceuticals to the environment and human health.

Studies around the world report the presence of different classes of pharmaceuticals in the environment [3]. To determine whether the presence of these pollutants is punctual, or continuous, long-term monitoring campaigns are essential. The primary objective of this study is to analyze pharmaceuticals in samples collected during the years 2018 and 2019 and compare them with the results obtained in previous years, 2013 [17], 2014 [17], and 2017 [18]. The collected samples include river water, as well as effluent and influent samples from WWTPs. Solid phase extraction (SPE) was employed for the extraction process, followed by analysis using ultra-high performance liquid chromatography with tandem mass spectrometry (UHPLC-MS/MS). All samples analyzed in this study were collected from the same sampling sites [17,18].

This study covers a wide range of pharmaceutical compounds, including their metabolites and degradation products, and comprises those listed in the EU watch lists [19,20,21,22] (six out of 10; namely, sulfamethoxazole, trimethoprim, venlafaxine, *O*-desmethylvenlafaxine, ofloxacin, and metformin) and those regulated by the Swiss Water Protection Act (five out of eight in group I; namely, carbamazepine, citalopram, clarithromycin, diclofenac, and venlafaxine). The Act mandates advanced treatment for WWTPs to achieve an 80% reduction in indicative organic micropollutants [23].

As stated in 2023 by Liu et al. [16], globally, the pharmaceuticals most commonly detected in water samples are antibiotics (erythromycin, sulfamethoxazole, and trimethoprim); antiepileptics, especially carbamazepine; nonsteroidal anti-inflammatory drugs (NSAIDs), particularly ibuprofen; and the analgesic acetaminophen. In the United States, the Environmental Protection Agency (EPA) has released guidelines for establishing national water quality criteria using aquatic toxicological data. These guidelines facilitate the assessment of the hazards posed by pharmaceuticals and personal care products to aquatic life. Although these guidelines do not specify priority compounds, they suggest the need for increased attention to identify the ecotoxicological risks associated with pharmaceuticals and personal care products [16]. In Sweden, data related to environmental hazard assessment, bioaccumulation, toxicity and persistence of a comprehensive classification of marketed pharmaceuticals have been made available online since 2006 and the classification has been enhanced in 2010 [24]. In 2015 the European Union’s Watch List for emerging pollutants included pharmaceuticals for the first time, recognizing their potential ecological relevance. This list served as a starting point for many countries and regions to develop their own monitoring programs. Between 2015 and 2022, the watch lists for pharmaceuticals have evolved significantly, reflecting an increasing concern about their presence in the environment.

Compared to other studies, the present study stands out due to the number of pharmaceuticals analyzed, specifically, 83 pharmaceuticals in the monitoring campaigns and 33 pharmaceuticals in the temporal analysis, covering various therapeutic classes. The set of pharmaceutical compounds commonly monitored in recent years was focused on the most consumed and the ones included in the Watch Lists [19,20,21,22]. However, according to the proposal for the new directive concerning urban wastewater treatment [23], monitoring campaigns must include the pharmaceuticals on its list, which were also included in this study. It is also important to highlight the different matrix types of samples, which include surface water (river) and wastewater samples (influents and effluents). It is worth noting the geographical extension of the sampling area; the Lis River was monitored along its entire course, from its source to its mouth, thus evaluating the impact of discharges from the two wastewater treatment plants (WWTPs) that discharge their effluents into this river, as well as unknown inputs. Moreover, the samples were collected at the same sampling points throughout a long monitoring period (2013 to 2019), allowing the evaluation of the contamination evolution. Therefore, this study aims to gather essential information, regarding: (i) pharmaceuticals that enter the environment, (ii) metabolites and degradation products detected, (iii) identification of the presence and persistence of these compounds, (iv) acquiring knowledge about the levels found in the collected samples, (v) analysis of the pollution profile along the river course, and (vi) examination of temporal variations throughout the study years. The information obtained provides valuable insights for responsible authorities, researchers, and WWTP staff in their continuous efforts to effectively assess and manage environmental conditions.

## 2. Materials and Methods

### 2.1. Sampling Campaign (2013–2017)

In 2013/2014, 33 pharmaceuticals (including NSAIDs/analgesics, antibiotics, and psychiatric drugs) were assessed over 11 months in Lis River samples and nine months in influents and effluents from two WWTPs (Olhalvas (1) and Coimbrão (2)) [17]. Effluents E1 and E2 are discharged into the Lis River (Figure S1, Supplementary Materials). Then, in 2017, additional analytes belonging to several therapeutic classes were analyzed to monitor concentration variations in WWTP effluents and influents (WWTP 2) over 24 h [18].

### 2.2. Sampling Campaign (2018–2019)

The 2018 and 2019 research aimed to analyze the same types of samples collected during the 2013/2014 sampling campaigns (SCs) [17], which included river water, as well as effluents and influents from two WWTPs (Figure S1, Supplementary Materials). This study focused on assessing 83 pharmaceuticals as investigated in the 2017 study [18], thus maintaining continuity with previous research efforts. The samples collected in the SCs of 2018 and 2019 were taken from the same sampling points as those collected in 2013 and 2014.

### 2.3. River and WWTPs under Study

Lis River flows from its source, at Fontes, to the Beach of Vieira, in the Atlantic Ocean coast, passing through both rural fields and urban areas, such as the city of Leiria located in the Central Region of Portugal. It receives inputs from industrial, agricultural, and livestock activities, as well as effluents from WWTPs [25]. Freshwater pollution problems are gaining attention regionally due to their social, economic, and health impacts. Rivers are fundamental resources for domestic, industrial, and irrigation purposes. Consequently, understanding and managing pollution is of utmost importance. Localization, type of treatment, hydraulic retention time, sludge retention time, average flow rate, and equivalent population (design and served) of both WWTPs are listed in Table S1 (Supplementary Materials).

### 2.4. Reagents and Materials

Reagents, eluents, materials and equipment (Table S2), pharmaceuticals, metabolites, degradation products, isotopically labeled internal standards (ILIS), CAS [26,27], molecular formula, molecular weight, supplier company, solvents used for preparing the stock solutions (Table S3), and ILIS concentration in standards and samples (Table S4) are provided in the Supplementary Materials. Stock standards for each analyte were prepared at 1 g/L and stored at −20 °C. Working standard solutions containing all compounds were prepared in acetonitrile/ultra-pure water (30:70, *v*/*v*). A mixture of ILIS was prepared for internal standard calibration.

### 2.5. Sample Pre-Treatment and Sampling Points

Before collection, bottles were rinsed with ultrapure water and then with the sample itself. Surface water and wastewater were collected in high-density polyethylene bottles. River samples were taken at the source (SP1), upstream (SP2 and SP4), and downstream of each WWTP (SP3 and SP5), located approximately 500 m from the effluent discharge (Figure S1, Supplementary Materials). Wastewater samples were composite samples collected at regular intervals (1 h) by mixing grab samples taken over 24 h using an automatic sampler. In each sampling campaign (SC), samples were transported in hermetic boxes refrigerated with ice, and upon arrival at the laboratory, they were vacuum-filtered through a 0.45 µm nylon membrane filter, immediately extracted using SPE and analyzed by UHPLC-MS/MS.

### 2.6. Analytical Method

The analytical method employed for the extraction and analysis of pharmaceuticals, metabolites, and degradation products in wastewaters (WWTP influent and effluent) and river water samples has been described in authors’ previous works [17,18,28,29]. The target compounds were extracted using Strata-X SPE cartridges (200 mg, 3 mL). A suitable volume of a 0.1 M Na_2_EDTA solution was added to the samples (50 mL of WWTP influent, 100 mL of WWTP effluent, or 250 mL of river water) to achieve a final concentration of 0.1% (g solute/g solution). After, the pH of each sample was adjusted to pH 2 with HCl 37%. Then, pre-treated samples passed through conditioned (5 mL methanol) and equilibrated (5 mL ultrapure water and 5 mL ultrapure water pH 2) SPE cartridges. The analytes were eluted with 10 mL of methanol, evaporated until dryness under nitrogen, and reconstituted with 500 µL of acetonitrile:water (30:70, *v*/*v*). The SPE procedure is illustrated in Figure S2 (Supplementary Materials). Then, 5 µL of the ILIS mixture was added to both standards and samples and the final concentration of the ILIS is presented in Table S4 (Supplementary Materials). Instrumental analysis was conducted using a UHPLC-MS/MS coupled to a triple-quadrupole mass spectrometer operated in electrospray ionization (ESI) mode (Table S2, Supplementary Materials), with Lab Solutions LCMS software (version 5.80, Shimadzu, Kyoto, Japan) used for system control and data processing. Quantification of the analytes was performed by Multiple Reaction Monitoring (MRM). Detailed information can be found in Tables S5 and S6 in the Supplementary Materials.

### 2.7. Analytical Method Validation

Linearity, method detection (MDLs) and method quantification (MQLs) limits, precision (intra- and inter-day), recovery, and matrix effect (ME) were evaluated for validation purposes. Calibration curves were constructed using linear regression analysis, correlating peak area with analyte concentration. MDLs and MQLs were determined based on the minimum detectable amount of each analyte at signal-to-noise ratios. Intra- and inter-day precision were assessed by calculating the relative standard deviation (RSD, %). Standard solutions containing all analytes (250 μg/L) were consecutively injected. Recovery was determined by comparing quantification ion areas of pre-spiked samples with those of post-spiked samples after SPE extraction. ME was evaluated by comparing standard areas prepared in matrix and solvent (acetonitrile/ultrapure water 30:70, *v*/*v*) at a concentration of 250 μg/L for each analyte. Figure S3 (Supplementary Materials) illustrates the equations and schemas used for ME determination. Absence of ME, ion enhancement signal, and ion suppression signal were possible, with values within −20% to +20% indicating minor matrix effects [30].

### 2.8. Environmental Risk Characterization

The risk that may represent to the aquatic environment of the detected pharmaceuticals in the study river was estimated through the risk quotient (RQ), calculated following the EU guidelines [31] using the following equations:

(1) $$\mathrm{RQ}=\frac{\mathrm{Highest}\;\mathrm{concentration}\;\mathrm{detected}\;(\mathrm{ng}/L)}{\mathrm{PNEC}\;(\mathrm{ng}/L)}$$

(2) $$\mathrm{PNEC}\;\left(\frac{\mathrm{ng}}{L}\right)=\frac{\mathrm{LC}50\;\mathrm{or}\;\mathrm{EC}50\;}{\mathrm{AF}}\times 10^{6}$$

where RQ is the risk quotient; PNEC is the predicted no-effect concentration (PNEC calculation from ECHA guidance on chemical risk assessment); AF is the assessment factor (The assessment factor [AF] considering the type of sample [water] and at least one short-term LC50 or EC50 from each of the three trophic levels is 1000 [32]); LC50 (mg/L) is the concentration of a pollutant that is lethal for 50% of the exposed population; EC50 (mg/L) is the concentration of a pollutant necessary to produce an effect in 50% of the exposed population; and 1 × 10^6^ is the conversion factor from mg/L to ng/L. 

The ECOSAR predictive model (v1.11) [33] was used to determine EC50 or LC50 values for each compound across three trophic levels of the aquatic ecosystem (algae, *Daphnia magna*, and fish). Risk quotient (RQ) calculations were conducted based on the worst-case scenario, employing the highest concentration for each detected analyte. For concentrations below the MDL or MQL, half of the MQL value was assumed [17]. RQ values equal to or exceeding 1 indicate potential environmental risk, while values below 1 suggest negligible risk [34].

## 3. Results and Discussion

### 3.1. Validation Results

#### 3.1.1. Linearity and Correlation Coefficients

Method linearity was confirmed graphically across a concentration range of 0.5 to 1000 μg/L (12 levels), with correlation coefficients (R) exceeding 0.997, indicating a strong linear relationship between the analyte concentration and the peak area. Retention time and ion ratios for each analyte are detailed in Table S7 (Supplementary Materials).

#### 3.1.2. Recovery Tests

**Figure 1:**
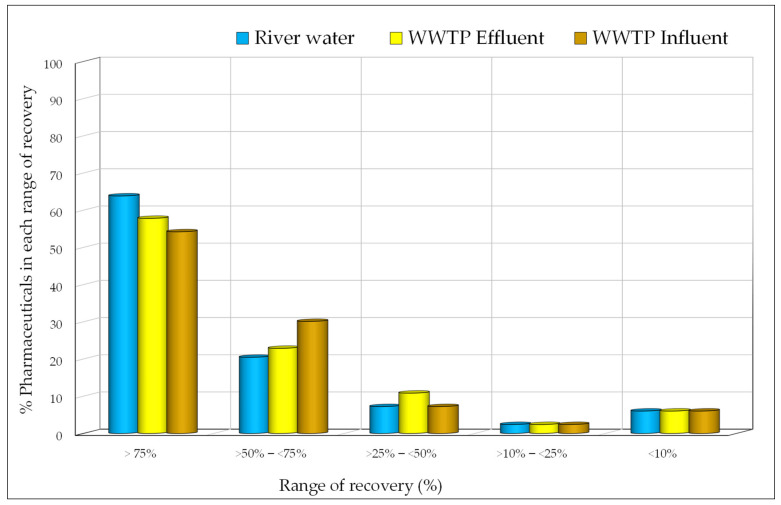
Percentage of the studied compounds in each range of recovery.

Recovery tests involved three fortification levels per matrix, with two extractions per level. Results were consistent for all levels, showing RSD < 10%. Average recoveries are summarized in Table S8 (Supplementary Materials). Generally, SPE extraction yielded satisfactory recoveries for most compounds in river water and wastewater matrices. Nimesulide achieved 127% recovery in river water, norfluoxetine 114% in WWTP effluent, and ibuprofen 113% in WWTP influent. Additionally, 63.9% (river water), 57.8% (WWTP effluents), and 54.2% (WWTP influents) of compounds showed recoveries above 75% (Figure 1). Lowering sample pH to 2 enhanced analyte interaction with the Strata-X adsorbent, resulting in good recoveries for most analytes. However, cathine, lansoprazole, metformin, dl-norephedrine, and synephrine exhibited recovery values <10% in all matrices. At pH 2, these pharmaceuticals are charged and, therefore, are not being effectively retained in the Strata-X cartridge.

#### 3.1.3. Matrix Effect

**Figure 2:**
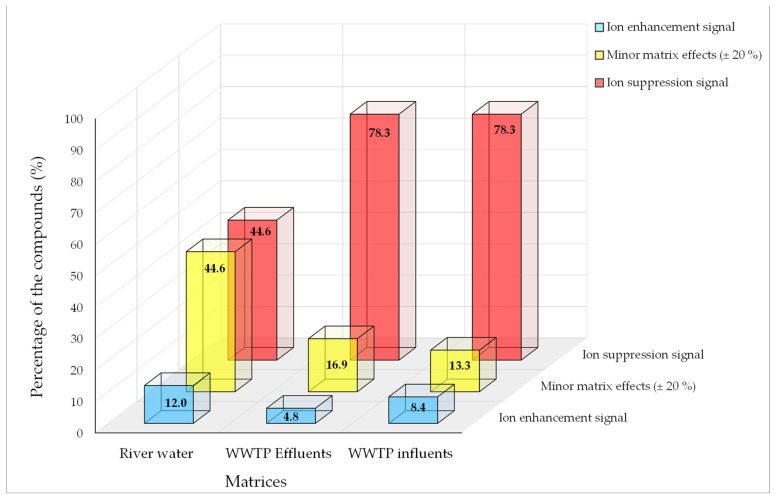
Percentage of the compounds in the three groups of matrix effect: ion enhancement, minor matrix effect, and ion suppression.

Matrix effects (ME) in river water, WWTP effluent, and WWTP influent were assessed using the equation presented in Figure S3 (Supplementary Materials). As expected, in general, higher ME was observed in wastewaters when compared with surface water (Figure S4). The highest ion enhancement was observed for lansoprazole in river water, tetracycline in WWTP effluent, and oxytetracycline in WWTP influent matrices and the highest signal suppression was observed for rimonabant in river water, naproxen in WWTP effluent, and erythromycin in WWTP influent, respectively. It is important to highlight that atorvastatin and the antibiotics azithromycin, oxytetracycline, tetracycline, doxycycline, and chlorocycline were the pharmaceuticals with ion enhancement in all matrices. In the river water matrix, 44.6% of analytes showed minor ME and ion suppression. In wastewater matrices, 78.3% displayed ion suppression. Ion enhancement was observed for 12.0%, 4.8%, and 8.4% of compounds in river water, WWTP effluent, and WWTP influent matrices, respectively (Figure 2).

Other studies also noted increased matrix effects (ME) in wastewater samples [35,36,37,38]. Borecka et al. [39] evaluated ME across various water samples, observing signal suppression, particularly in wastewater samples. They attributed this suppression to the higher organic matter content in these matrices. Steen et al. (1999) [40] demonstrated that co-extracted organic matter like humic acids can cause signal suppression in ESI-MS detection.

#### 3.1.4. Intra- and Inter-Day Precision

The method led to good precision values, with RSD (%) of intra- and inter-day analysis lower than 10%.

#### 3.1.5. Method Detection and Method Quantification Limits

MDL and MQL were determined for all matrices where pharmaceuticals were detected. Fortified samples were used otherwise (Figure S5, Supplementary Materials). Limits ranged from 0.0200 to 57.7 ng/L in river water, 0.0500 to 168 ng/L in WWTP effluent, and 0.100 to 391 ng/L in WWTP influent. As observed, higher MDL values were found in WWTP influent matrix compared to WWTP effluents and river water. The lowest MDL values showed high sensitivity and revealed qualities of UHPLC-MS/MS for accurate quantification and confirmation of trace levels of the pharmaceuticals, metabolites, and degradation products in environmental samples.

### 3.2. Sampling Campaign Performed in 2018 and 2019

**Figure 3:**
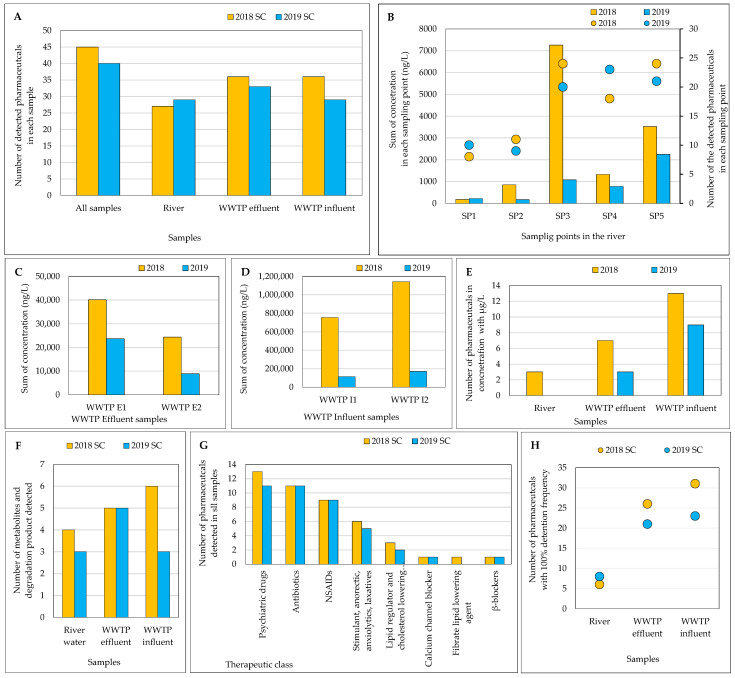
Results for each type of sample (river water and WWTP effluent and influent samples) in the sampling campaigns performed in 2018 and 2019: (**A**) Number of detected pharmaceuticals; (**B**) Sum of the concentrations and number of detected pharmaceuticals in each sampling site; (**C**) Sum of the concentrations of detected pharmaceuticals in WWTP effluent samples; (**D**) Sum of the concentrations of detected pharmaceuticals in WWTP influent samples; (**E**) Number of pharmaceuticals with concentration at µg/L level, (**F**) Number of metabolites and degradation product; (**G**) Number of pharmaceuticals detected by therapeutic class; and (**H**) Number of pharmaceuticals with 100% of detection frequency.

The SCs were carried out in winter 2018 and spring 2019. The concentrations of the detected analytes are presented in Tables S9 and S10 of Supplementary Materials and Figure 3. The main conclusions of the obtained results are listed in Tables S11 and S12 (Supplementary Materials) and the discussion of the obtained results is presented in the following subsections.

#### 3.2.1. Number of Analytes Detected in Each Sampling Campaign

Of the 83 target analytes, 45 in 2018 and 40 in 2019 were detected in at least one sample (Figure 3A). The difference between the two SCs was the detection of didemethylcitalopram, diazepam, ephedrine, phentermine, pravastatin, and fenofibrate in the SC performed in 2018, and fenfluramine was detected only in the SC performed in 2019. Twenty-two pharmaceuticals were detected in all matrices across both years, highlighting their consistent presence (Tables S9 and S10, Supplementary Materials). More pharmaceuticals were found in the wastewaters samples when compared with the number of pharmaceuticals detected in river samples. There are several factors that influence the presence, and, therefore, the detection, of compounds as well as their concentration levels in surface waters and wastewaters. For example: (i) the fact that certain medications are taken more frequently during specific times of the year, (ii) the season in which samples were taken, (iii) meteorological conditions; (iv) removal of the compound by the WWTP, (v) deconjugation or reversion back to the parent form during biological treatments, (vi) adsorption or desorption of the compound to or from sludges, and (vii) unknown discharges that can lead to an increase of the concentration levels in samples collected from river water. Although most pharmaceuticals are detected at ng/L levels in the environment (Tables S9 and S10, Supplementary Materials), their concentrations in analyzed samples should be noted. Ankley et al. [41] highlighted the ecological concern posed by pharmaceuticals due to their long-term adverse effects on humans and animals. Identifying pollution sources, testing various WWTP treatments, and monitoring environmental pollution are crucial for minimizing pharmaceutical release into terrestrial and aquatic systems, even at low concentrations [42,43].

#### 3.2.2. Main Results Obtained in River Water Samples

The results indicate an increase in both the number of detected analytes and their total concentrations along the river course, with the highest values generally observed downstream of both WWTPs (SP3 and SP5), highlighting their influence on the river (see Figure 3B). Goulart et al. similarly noted higher concentrations downstream of effluent discharge, underscoring the significant role of WWTPs in pollutant presence and concentration variation in rivers [44].

Lis River faces ongoing pollution from WWTP effluent discharges and unidentified inputs [25]. Detection of study compounds at SP1 and SP2 (located upstream of the first effluent discharge—Figure S1, Supplementary Materials) suggests the presence of these unknown inputs. Five NSAIDs (ibuprofen, hydroxyibuprofen, acetaminophen, salicylic acid, and ketoprofen) and caffeine were detected at both SP1 and SP2 in both SCs. Additionally, carbamazepine and venlafaxine were found in 2018, while carbamazepine, fluoxetine, sulfadiazine, and trimethoprim were found in 2019 at SP1. At SP2, citalopram, propionic acid, fluoxetine, venlafaxine, topiramate, and gemfibrozil were detected in 2018, and carbamazepine, fluoxetine, and nimesulide in 2019. Detection of caffeine is significant as indicates anthropogenic pollution [45,46,47,48]. Another crucial sampling point along Lis River is SP4, which could indicate unknown inputs to the river. Positioned downstream of SP3 and upstream of WWTP2 effluent discharge (Figure S1, Supplementary Materials), SP4’s analyte levels are expected to be lower than SP3 due to dilution in the river, and lower than SP5 due to WWTP2 discharge. However, exceptions were observed, such as higher concentrations of acetaminophen in 2018, and sulfamethazine, sulfapyridine, carboxyibuprofen, ketoprofen, and caffeine in 2019 at SP4 compared to SP3 and SP5. Additionally, tetracycline (SP3) and phenolphthalein (SP3, SP4, and SP5) were detected in 2018, and sulfamethazine (SP4) and nimesulide (SP2) in 2019 were found only in river water samples (Tables S9 and S10, Supplementary Materials), which may indicate untreated wastewater discharges near these points or other sources of contamination such as leaching from cattle-raising activities.

Analytes at µg/L levels were only detected in the 2018 SC, including carboxyibuprofen (SP3), hydroxyibuprofen (SP3 and SP5), and caffeine (SP3). The lowest concentrations (<MDL) were found for fluoxetine (SP2, SP3, SP4, and SP5), acetaminophen (SP1), ketoprofen (SP1, SP2, and SP4), phenolphthalein (SP3, SP4, and SP5), and diltiazem (SP3, SP4, and SP5) in 2018, and for citalopram (SP4), bupropion (SP4), ofloxacin (SP3 and SP4), trimethoprim (SP1), and diltiazem (SP4) in 2019.

#### 3.2.3. Main Results Obtained in the Wastewater Samples

A higher number of pharmaceuticals (Figure 3A) and a higher sum of concentrations were found in wastewaters (river < WWTP effluent < WWTP influent) (Figure 3B–D), with the highest concentration being observed in WWTP influent samples.

The highest concentrations (µg/L) were detected for 17 compounds in the SC of 2018 and 10 compounds in the SC of 2019. Notably, hydroxyibuprofen, diclofenac, ibuprofen, naproxen, atenolol, and caffeine showed the highest concentrations in both years. It is worth noting that 41% (2018) and 70% (2019) of the compounds detected at µg/L levels belong to the NSAIDs/analgesics group (Tables S9 and S10, Supplementary Materials). In the analyzed wastewater samples, concentrations below the MDL were observed for several compounds. For instance, in the 2018 SC, fluoxetine (I1, I2), trimethoprim (E2, I1, and I2), simvastatin (E1), diltiazem (E1, E, I1, and I2), and fenofibrate (I2) were detected below the MDL. Similarly, in the 2019 SC, ofloxacin (I1), sulfadiazine (E2), and dl-methamphetamine (E2) were found below the MDL (Tables S9 and S10, Supplementary Materials).

Another important consideration is the detection of pharmaceuticals in wastewater samples (effluent and influent) but not in river water. Specifically, 10,11-epoxycarbamazepine, ciprofloxacin, ofloxacin, and sulfamethoxazole were detected in both SCs, while acetylsalicylic acid, atenolol, and fenofibrate were detected only in the 2018 SC, and ciprofloxacin and atenolol were detected only in 2019. The absence of these compounds in the river can be related to several factors, such as treatment processes, dilution, and degradation, as well as their physicochemical properties. Before reaching the surface waters, pharmaceuticals and metabolites pass through the WWTP, where they may undergo removal (e.g., biodegradation), resulting in lower or undetectable levels in the effluent and consequently in the receiving waters. This explains why they are detected in influents but not in effluents and consequently also not detected in surface waters. When treated wastewater is discharged, it is diluted by the volume of the receiving waters, significantly reducing the concentration of the contaminants. Natural processes in the aquatic environment, such as sunlight exposure, microbial degradation, and chemical reactions, can further break down or transform these compounds into other forms, resulting in lower concentrations or non-detectable levels of the studied compounds. Another consideration is the octanol–water partition coefficient (log Kow). Positive values for log Kow indicate hydrophobic behavior, and higher values reveal extreme hydrophobicity. Molecules with low (log Kow < 1) or negative values for log Kow are frequently classified as polar [49]. Hydrophilic compounds may remain dissolved in the aqueous phase, while more hydrophobic substances may bind to biosolids and sediments [50]. The hydrophobic properties of 10,11-epoxycarbamazepine and fenofibrate compounds limit their solubility in water and tend to associate with organic matter or sediment particles [50,51]. Acetylsalicylic acid exhibits moderate hydrophobicity (log Kow 1.19) and also can be adsorbed to organic matter [51]. On the other hand, ciprofloxacin, ofloxacin, sulfamethoxazole, and atenolol have log Kow values lower than 1 [51]. Consequently, if not completely removed in the WWTP, the absence of detection in the river is likely due to dilution factors, as these compounds exhibit hydrophilic properties.

Comparing both SCs, a higher sum of concentrations of pharmaceutical compounds was observed in the samples collected in the SC performed in 2018. Also, Kibuye et al. [52] reported higher pharmaceutical concentrations in the WWTP wastewaters (influent and effluent) in the winter season. The number of analytes with concentration in the µg/L range increases with the complexity of the matrix (Figure 3E), which is in line with what would be expected. WWTP influent is a mixture of wastewaters, from various sources, that did not undergo any treatment process and, therefore, the concentration in influent is expected to be higher.

#### 3.2.4. Pharmaceuticals and Their Transformation Products

In the present study, 10 metabolites and one degradation product were included. As can be seen in Figure 3F, these compounds were detected either in river samples or in wastewater samples.

Ibuprofen metabolites, hydroxyibuprofen and carboxyibuprofen, were analyzed. As carboxyibuprofen is excreted in higher percentages, it would be expected to be found in the environment at higher concentrations when compared to ibuprofen and hydroxyibuprofen [53]. The results are not always a mirror of what is expected. Carboxyibuprofen was detected in seven out of 18 samples (SP3, I1, and I2 of 2018 SC and SP3, SP4, I1, and I2 of 2019 SC) and hydroxyibuprofen was the metabolite detected in all samples. It is important to highlight that carboxyibuprofen was not detected in any WWTP effluent samples, showing its high removal rate in studied WWTP. On the other hand, when carboxyibuprofen is detected, in most of the analyzed samples, it was always with a higher concentration when compared with hydroxyibuprofen and ibuprofen (Tables S9 and S10, Supplementary Materials). Similar conclusions were reported in other studies [17,18,53]. Ferrando-Climent et al. and Winkler et al., noted that hydroxyibuprofen demonstrates greater stability than ibuprofen and carboxyibuprofen [53,54]. Given its high biodegradability in the environment, the presence of carboxyibuprofen in the samples may be attributed to recent discharges from either WWTP effluents or untreated effluents [55].

The metabolite 10,11-epoxycarbamazepine was detected in WWTP effluent (E1) in 2018 and 2019, as well as in WWTP influents (I1) in 2018, but never in the river (Tables S9 and S10, Supplementary Materials). The concentration in E1 is higher than that found in the corresponding influent. Several studies reported that the excreted conjugates can be cleaved by enzymes during the WWTP treatments, converting the metabolites to the parent compound form [17,18,56,57]. Another consideration is the possible release of compounds through desorption. These compounds, owing to their hydrophobic properties, become adsorbed onto the biomass (sludge) during biological treatment. The subsequent release of these compounds, through desorption from the sludge, results in an increase in their concentration in WWTP effluents, potentially leading to a negative removal rate. Similar findings have been reported for psychiatric drugs by several researchers including Jelic et al. [58], Joss et al. [59], Papageorgiou et al. [60], Petrie et al. [61], Bahlmann et al. [56], Castiglioni et al. [62], and Verlicchi et al. [63].

In the case of citalopram, three metabolites were found, namely: citalopram propionic acid (river in 2018, WWTP effluents in 2018 and in 2019, and WWTP influent in 2018), desmethylcitalopram (WWTP influents in 2018 and 2019), and didemethyl citalopram (WWTP effluent in 2018). Concentrations ranged from 9.34 to 24.0 ng/L in the river and between 28.1 and 198 ng/L in wastewaters (Tables S9 and S10, Supplementary Materials), respectively. Citalopram metabolites were in general detected with a higher frequency and with a higher concentration in WWTP effluents. Similarly, a negative removal rate was observed, as mentioned for psychiatric drugs in the studies cited in the preceding paragraph [56,58,59,60,61,62,63].

Salicylic acid is the major metabolite of acetylsalicylic acid [64]. However, there are other sources that salicylic acid can derive. In addition, salicylic acid is highly irritant and is used only externally for skin problems [65], in toothpaste, and as a food preservative [64]. Salicylic acid can also be a degradation product as acetylsalicylic acid is stable in dry air, but gradually, hydrolyses in contact with moisture to acetic and salicylic acids [66]. Detected in all samples, its concentrations varied from 83.6 to 274 ng/L (river), 196 to 247 ng/L (WWTP effluent), and 13.9 to 25.7 µg/L (WWTP influent) in 2018, and from 43.1 to 98.8 ng/L (river), 130 to 893 ng/L (WWTP effluent), and 4.08 to 28.9 µg/L (WWTP influent) in 2019.

#### 3.2.5. Therapeutical Classes

Psychiatric drugs, antibiotics, NSAIDs/analgesics, lipid regulator and cholesterol-lowering statin drugs, β-blockers, calcium channel blockers, fibrate lipid-lowering agents, and stimulants, anorectic, anxiolytics, and laxatives were the therapeutical classes detected in the analyzed samples (Figure 3G, and Tables S9 and S10, Supplementary Materials). Psychiatric drugs, antibiotics, and NSAIDs/analgesics were the most detected, consistent with previous studies [17,18].

NSAIDs/analgesics are widely consumed for their anti-inflammatory, analgesic, and antipyretic effects [67]. Acetaminophen, diclofenac, ketoprofen, naproxen, ibuprofen, hydroxyibuprofen, carboxyibuprofen, and salicylic acid were consistently detected, with acetylsalicylic acid found only in 2018 and nimesulide only in 2019. Concentrations reaching µg/L were observed for several of these compounds in at least one sample (Tables S9 and S10, Supplementary Materials).

The effectiveness of antibiotics faces serious threats from the rapid spread of resistant bacteria, emphasizing the danger of their uncontrolled use [68,69]. Azithromycin, clarithromycin, ciprofloxacin, ofloxacin, sulfamethoxazole, sulfapyridine, trimethoprim, and tetracycline were detected in both SCs, with additional antibiotics found exclusively in 2018 (oxytetracycline, alprazolam, and lorazepam) or 2019 (chlorocycline, sulfadiazine, and sulfamethazine). At the µg/L level, concentrations of two macrolide antibiotics (azithromycin and clarithromycin) and two fluoroquinolone antibiotics (ciprofloxacin and ofloxacin) were detected in wastewater (Tables S9 and S10, Supplementary Materials).

The global prescription of psychotropic drugs, including antipsychotics, antidepressants, and stimulants, has risen significantly over the past few decades [70]. Analyzed samples revealed the presence of several psychotropic drugs and their metabolites (carbamazepine, 10,11-epoxycarbamazepine, citalopram, citalopram propionic acid, desmethylcitalopram, didemethylcitalopram, fluoxetine, paroxetine, sertraline, trazodone, venlafaxine, bupropion, and diazepam), with carbamazepine showing the highest concentration in WWTP effluents and influents (Tables S9 and S10, Supplementary Materials).

#### 3.2.6. Detection Frequency

The number of pharmaceuticals with 100% detection frequency increases with sample complexity (Figure 3H). Venlafaxine, hydroxyibuprofen, ibuprofen, ketoprofen, salicylic acid, and caffeine were detected in all samples of the 2018 SC, while carbamazepine, fluoxetine, acetaminophen, hydroxyibuprofen, ibuprofen, ketoprofen, salicylic acid, and caffeine were found in all samples of the 2019 SC (Tables S9 and S10, Supplementary Materials). Hydroxyibuprofen, ibuprofen, ketoprofen, salicylic acid, and caffeine were consistently detected with 100% frequency in both SCs.

### 3.3. WWTP Removal Rate

High removal rates were observed for several pharmaceuticals in 2018, including desmethylcitalopram, diazepam, sulfapyridine, ephedrine, fentermine, dl-methamphetamine, and carboxyibuprofen, and in 2019, for venlafaxine, sulfamethoxazole, sulfapyridine, phenolphthalein, and carboxyibuprofen. However, negative removal rates were noted when pharmaceuticals were exclusively detected in WWTP effluents or when their concentration in effluents surpassed that in influents. Negative removal rates can be associated either with deconjugation or reversion back to the parent form, as is the case with metabolites transforming into their original form, resulting in increased concentrations of the parent compound. Additionally, when hydrophobic compounds are adsorbed onto organic matter, they can later be released through desorption from the sludge, leading to an increase in their concentration. Desorption occurs due to various factors such as pH and temperature changes, microbial activity, or the presence of other chemicals that compete for binding sites on the sludge particles. These factors can weaken the attraction between the compounds and the sludge, allowing the compounds to detach the sludges and re-enter the effluents.

For most of the compounds showing negative removal, they range from moderately hydrophobic (such as citalopram, paroxetine, carbamazepine, alprazolam, pravastatin, fenfluramine, and dl-methamphetamine) to extremely hydrophobic (such as azithromycin, simvastatin, and gemfibrozil). As mentioned earlier, due to their hydrophobic properties, pharmaceuticals and metabolites are adsorbed in sludge and can be subsequently released into the effluent due to desorption. Additionally, metabolites can also undergo deconjugation. All these phenomena lead to increased concentrations of certain compounds in WWTP effluents compared to the corresponding WWTP influents. Regarding oxytetracycline, trimethoprim, and tetracycline, these compounds have a log Kow < 1. One possible explanation may be due to the gradual release of the compounds from fecal particles, as also mentioned in the studies performed by Göbel et al. [71], Kagle et al. [72], and Alfonso-Muniozguren et al. [73]. Moreover, Kumar et al. (2022) reported that the negative removal rate observed for some compounds is a global phenomenon and a common feature for almost all types of WWTPs, making humans and the environment vulnerable. They concluded that conjugation–deconjugation, complex formation, compound transformation, and treatment processes are major controlling factors for negative removal. Furthermore, they highlighted the lack of data on adsorbed contaminants in the sludge/solid phase as an obstacle to understanding the negative removal potential of WWTPs [74].

### 3.4. Risk Assessment

The RQ is a valuable tool for assessing ecological risks in aquatic ecosystems [75]. The results from both SCs in river water samples are shown in Figure S6. Most compounds exhibited negligible effects, with RQ values below 0.1. However, several compounds showed RQ values between 0.1 and 1.0, indicating potential ecotoxicological risks. For instance, caffeine in *Daphnia magna*, citalopram in algae, and azithromycin, sertraline, and venlafaxine in *Daphnia magna* and algae in SC performed in 2018, as well as sulfamethazine, sulfamethoxazole, sulfapyridine, and trimethoprim in *Daphnia magna*, carbamazepine in algae, and sulfadiazine in *Daphnia magna* and algae in SC performed in 2019, had RQ values within this range. Caffeine (in both 2018 and 2019) and carbamazepine (2018) in algae with RQ > 1 indicated potential ecotoxicological risks. Notably, the highest RQ value was observed for caffeine in 2018. Caffeine, an indicator of urban pollution, suggests the influence of anthropogenic activities in the studied area. Carbamazepine, a widely used pharmaceutical, indicates the contribution of wastewater effluents to the contamination of the aquatic environment. Consistent with our findings, previous studies have highlighted the ecotoxicological risks associated with caffeine in urban rivers [76,77]. Moreover, Liu et al. [78] demonstrated RQ values exceeding 1 for caffeine and carbamazepine also in urban rivers, supporting the significant ecotoxicological risks posed by these compounds. Wang et al. [79] noted that due to the widespread use and incomplete removal, pharmaceutical and personal care products are continuously introduced into the receiving water, which gradually affects organisms and the environment, and ultimately human health. Given the observed RQ values, it is important to consider the potential impacts on aquatic ecosystems. Future monitoring efforts should focus on assessing the long-term effects of these compounds on aquatic organisms and ecosystems. Additionally, measures should be taken to reduce the input of pharmaceuticals into aquatic environments through improved wastewater treatment processes and public awareness campaigns. The results underscore the need for proactive management strategies to safeguard aquatic ecosystems from the adverse effects of pharmaceuticals.

### 3.5. Assessment of Pharmaceuticals from 2013 to 2019—Temporal Analysis

The temporal analysis focused solely on common pharmaceutical compounds, being analyzed 33 in all the SCs. Pharmaceuticals were grouped by their families and three therapeutical classes: NSAIDs/analgesics (acetaminophen, acetylsalicylic acid, salicylic acid, ibuprofen, carboxyibuprofen, hydroxyibuprofen, diclofenac, ketoprofen, naproxen, and nimesulide), antibiotics (azithromycin, ciprofloxacin, enrofloxacin, ofloxacin, clarithromycin, sulfadiazine, sulfadimethoxine, sulfamethazine, sulfamethoxazole, sulfamethoxypyridazine, sulfapyridine, and trimethoprim), and psychiatric drugs (carbamazepine, 10,11̶epoxycarbamazepine, citalopram, diazepam, fluoxetine, norfluoxetine, paroxetine, sertraline, norsertraline, trazodone, and venlafaxine). The 33 target compounds were analyzed in WWTP influent and effluent and surface water samples.

The study focuses on pharmaceuticals due to their high prescription rates, particularly for antibiotics and psychiatric drugs, which has garnered media attention. Additionally, the widespread use of NSAIDs/analgesics presents challenges for control, as they are prescribed for chronic inflammatory conditions and available over the counter for various ailments.

**Figure 4:**
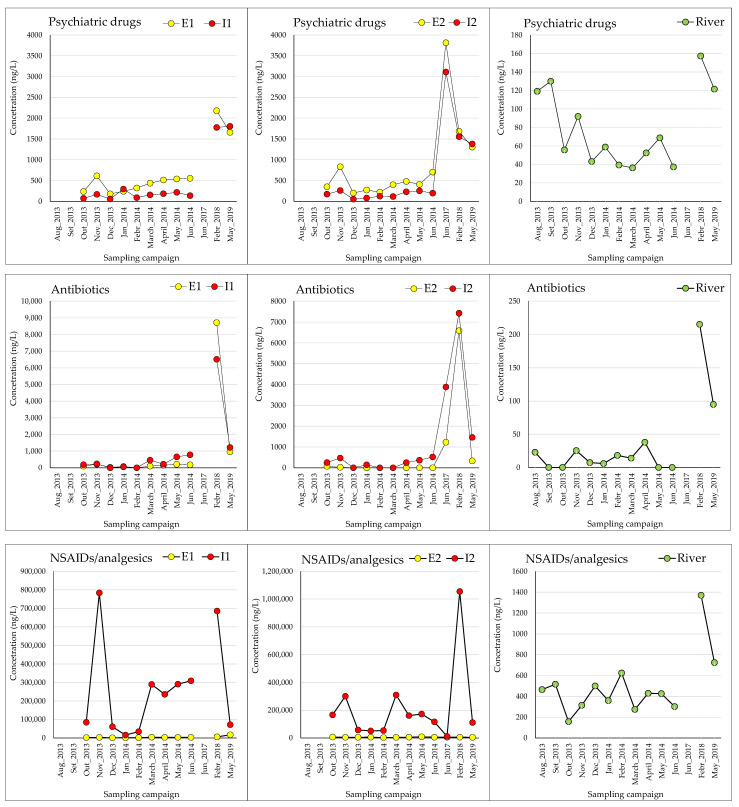
Temporal analysis for the study of pharmaceuticals during 5 years of campaigns (results from 2013 [17], 2014 [17], and 2017 [18] were published).

A total of 111 samples were analyzed from 2013 to 2019: 65 samples from river locations, 11 samples from WWTP E1 and I1, and 12 samples from WWTP E2 and I2. The results are summarized in Tables S13 to S21 (Supplementary Materials), with concentrations on graphs (Figure 4) representing sums within therapeutic families. River sample results are averages across five sampling points.

From 2013 to 2019, NSAIDs/analgesics consistently showed the highest concentrations, except for samples from WWTP-E1 and -E2 in the 2018 SC, where antibiotics were predominant (Figure 4). These findings are in line with the widespread usage of NSAIDs across all age groups of the population. Adams et al. (2011) found no significant association between advancing age and NSAID usage [80]. Across all sample groups, the highest concentrations were seen from 2017 to 2019, except for sample I1, where the peak occurred in November 2013. This suggests a gradual rise in pharmaceutical usage over time, consistent with OECD studies projecting increased pharmaceutical presence in the environment alongside rising consumption [81].

Meteorological fluctuations can cause inconsistent atmospheric conditions across seasons and years, complicating the correlation of pharmaceutical consumption with seasonal periods. Changes in wind patterns significantly affect weather and temperature annually. Paíga et al. noted a lack of noticeable seasonal variation in pharmaceutical presence in the study river [17], while Khan et al. suggested that seasonal agricultural practices, rainfall, and temperature could influence pharmaceutical residue levels and compositions in aquatic ecosystems [82]. In our study, samples collected across different years and seasons showed the highest concentration, particularly in the winter of 2018. Correspondingly, INFARMED data indicate a significant increase in medication purchases by Portuguese consumers in 2018 compared to previous years [83].

## 4. Conclusions

It is crucial to consider the safety and efficacy of medicines for human and animal health while also acknowledging their potential environmental impact. Risk assessment and environmental monitoring are necessary to prioritize pharmaceuticals for monitoring and regulation. Additionally, research into the treatment processes in WWTPs is essential, as many pharmaceuticals remain incompletely removed. This incomplete removal raises concerns about potential subtle effects on aquatic and terrestrial organisms. Among the therapeutic classes investigated, NSAIDs/analgesics, antibiotics, and psychiatric drugs showed a higher number of detected pharmaceuticals. Temporal analysis of the studied compounds was performed, and an increase in the concentration of detected pharmaceuticals was observed over time. For most of the analyzed samples, the sum of the concentration is higher between 2017 and 2019 when compared with the results found in 2013 and 2014. Although there were no changes in the operational conditions of the studied WWTP an increase in the total concentration of pharmaceutical compounds was observed, which is of great concern to the scientific community, water stakeholders, and policymakers, who should be committed to the challenge of reducing the entrance of these emerging contaminants in the environment. These findings highlight the importance of monitoring pharmaceuticals in the environment and shed light on their potential accumulation over time. Since WWTPs are one of the main sources of pharmaceuticals released into the environment, these studies also allow us to evaluate which are the most efficient treatments for eliminating these emerging pollutants and the results can contribute to improving the management of the WWTPs. These studies release crucial data that are important for humanity to be acutely aware of, emphasizing the urgent need to act.

Further monitoring studies should be performed in the surrounding areas of the largest WWTPs to better understand their impact on environmental contamination by pharmaceutical compounds, and to evaluate the necessity for quaternary treatment, as preconized by the revision of the Council and Parliament directive concerning urban wastewater treatment. Source identification (specific and diffuse) and developing mitigation strategies are essential to contribute to the development of policies and regulations to reduce pharmaceutical pollution, framed in the new directive concerning urban wastewater treatment of the European Union (EU), which is one of the deliverables under the EU’s zero-pollution action plan.

## Data Availability

Data are available upon reasonable request to the corresponding authors.

## Supplementary Materials

The following supporting information can be downloaded at: https://www.mdpi.com/article/10.3390/jox14030048/s1. Table S1: Localization, type of treatment, hydraulic retention time, sludge retention time, average flow rate and equivalent population for WWTP1 and WWTP2; Table S2: Reagents, eluents, materials, and equipments used in the extraction and analysis methodologies; Table S3: Pharmaceuticals, metabolites, degradation product, isotopically labeled internal standards (ILIS), chemical abstracts service (CAS), formula, molecular weight, supplier company, and solvent used for the preparation of each stock solution; Table S4: Concentration of the isotopically labeled internal standards (ILIS) in the standards and samples; Table S5: Chromatographic programs and temperature, gas flow, and energy used in the negative and positive ionization modes; Table S6: Therapeutic class, pharmaceuticals, ionization mode, precursor ions, product ions, mass spectrometry conditions, ion ratio, and isotopically labeled internal standards (ILIS) for each pharmaceutical in the study. Legend: P- Chromatographic program; Table S7: Retention time and ion ratio obtained for each study analyte; Table S8: Recoveries obtained in river water matrix, WWTP effluent and WWTP influent matrices; Table S9: Pharmaceuticals, metabolites and degradation products detected in each sample and their concentration (ng/L) in the sampling campaign of 2018; Table S10: Pharmaceuticals, metabolites and degradation products detected in each sample and their concentration (ng/L) in the sampling campaign of 2019; Table S11: Number of the compounds, metabolites, degradation products, therapeutical classes, detection frequency, and concentration in µg/L of the detected analytes in the sampling campaign performed in 2018; Table S12: Number of the compounds, metabolites, degradation products, therapeutical classes, detection frequency, and concentration in µg/L of the detected analytes in the sampling campaign performed in 2019; Table S13: Concentrations of the pharmaceuticals detected during five years in sampling point R1; Table S14: Concentrations of the pharmaceuticals detected during five years in sampling point R2; Table S15: Concentrations of the pharmaceuticals detected during five years in sampling point R3; Table S16: Concentrations of the pharmaceuticals detected during five years in sampling point R4; Table S17: Concentrations of the pharmaceuticals detected during five years in sampling point R5; Table S18: Concentrations of the pharmaceuticals detected during five years in WWTP Effluent E1; Table S19: Concentrations of the pharmaceuticals detected during five years in WWTP Effluent E2; Table S20: Concentrations of the pharmaceuticals detected during five years in WWTP Influent I1; Table S21: Concentrations of the pharmaceuticals detected during five years in WWTP Influent I2; Figure S1: Scheme of sampling points in the Lis River (SP1 to SP5) and WWTP influents (WWTP I1 and WWTP I2) and effluents (WWTP E1 and WWTP E2); Figure S2: Solid phase extraction procedure used for the extraction of the studied compound in river water and in wastewaters (effluent and influent) samples; Figure S3: Procedures used for the matrix effect determination in the analyzed matrices; Figure S4: Matrix effect in the river water and WWTP effluent and influent matrices; Figure S5: Method detection limit for the studied compound for river water and WWTP effluent and influent matrices; Figure S6: Risk Quotient obtained in each trophic level: (a) lower risk (RQ < 0.1), (b) moderate risk (0.1 < RQ < 1), and (c) high risk (RQ > 1).
