# Supplementary material for: Temporal Analysis of Pharmaceuticals as Emerging Contaminants in Surface Water and Wastewater Samples: A Case Study
Source: J Xenobiot. 2024 Jul 3;14(3):873–92. doi: 10.3390/jox14030048 (PMC11270430; doi:10.3390/jox14030048)
Supplement: Supplementary file 1 [file jox-14-00048-s001.zip › jox-2952777-supplementary.pdf]

Supplementary Materials (SM)

for

Temporal Analysis of Pharmaceuticals as Emerging Contaminants in Surface Water and Wastewater Samples: A Case Study

Paula Paíga [1,*,†], Luísa Correia-Sá [1,†], Manuela Correia [1], Sónia Figueiredo [1], Joana Vieira [2], Sandra Jorge [2], Jaime Gabriel Silva [3,4] and Cristina Delerue-Matos [1,*]

[1] REQUIMTE/LAQV, Instituto Superior de Engenharia do Porto, Instituto Politécnico do Porto, Rua Dr. António Bernardino de Almeida, 431, 4249-015 Porto, Portugal;; mlsrs@isep.ipp.pt (L.C.-S.); mmb@isep.ipp.pt (M.C.); saf@isep.ipp.pt (S.F.)
[2] Águas do Centro Litoral, SA, Grupo Águas de Portugal, ETA da Boavista, Avenida Dr. Luís Albuquerque, 3030-410 Coimbra, Portugal; j.vieira@adp.pt (J.V.); s.jorge@adp.pt (S.J.)
[3] Águas do Douro e Paiva, SA, Grupo Águas de Portugal, Rua de Vilar, 235 5º 4050-626 Porto, Portugal; g.silva@adp.pt (G.S.)
[4] Departamento de Engenharia Civil, Instituto Superior de Engenharia do Porto, Instituto Politécnico do Porto, Rua Dr. António Bernardino de Almeida, 431, 4249-015 Porto, Portugal; g.silva@adp.pt (G.S.)
[*] Correspondence: pcpa@isep.ipp.pt (P.P.); cmm@isep.ipp.pt (C.D.-M.); Tel.: +351-228340500 (P.P. & C.D.-M.); Fax: +351-228321159 (P.P. & C.D.-M.)
[†] These authors contributed equally to this work.

Number of pages: 37

Number of tables: 21

Number of figures: 5

List of tables

Tables		Page
Table S1	Localization, type of treatment, hydraulic retention time, sludge retention time, average flow rate and equivalent population for WWTP1 and WWTP2.	4
Table S2	Reagents, eluents, materials, and equipments used in the extraction and analysis methodologies.	5
Table S3	Pharmaceuticals, metabolites, degradation product, isotopically labeled internal standards (ILIS), chemical abstracts service (CAS), formula, molecular weight, supplier company, and solvent used for the preparation of each stock solution.	6-8
Table S4	Concentration of the isotopically labeled internal standards (ILIS) in the standards and samples.	9
Table S5	Chromatographic programs and temperature, gas flow, and energy used in the negative and positive ionization modes.	10
Table S6	Therapeutic class, pharmaceuticals, ionization mode, precursor ions, product ions, mass spectrometry conditions, ion ratio, and isotopically labeled internal standards (ILIS) for each pharmaceutical in the study. Legend: P-Chromatographic program.	11-12
Table S7	Retention time and ion ratio obtained for each study analyte.	13-14
Table S8	Recoveries obtained in river water matrix, WWTP effluent and WWTP influent matrices.	15-16
Table S9	Pharmaceuticals, metabolites and degradation products detected in each sample and their concentration (ng/L) in the sampling campaign of 2018.	17
Table S10	Pharmaceuticals, metabolites and degradation products detected in each sample and their concentration (ng/L) in the sampling campaign of 2019.	18
Table S11	Number of the compounds, metabolites, degradation product, therapeutical classes, detection frequency, and concentration in µg/L of the detected analytes in the sampling campaign performed in 2018.	19-20
Table S12	Number of the compounds, metabolites, degradation products, therapeutical classes, detection frequency, and concentration in µg/L of the detected analytes in the sampling campaign performed in 2019.	21-22
Table S13	Concentrations of the pharmaceuticals detected during five years in sampling point R1.	23
Table S14	Concentrations of the pharmaceuticals detected during five years in sampling point R2.	24
Table S15	Concentrations of the pharmaceuticals detected during five years in sampling point R3.	25
Table S16	Concentrations of the pharmaceuticals detected during five years in sampling point R4.	26
Table S17	Concentrations of the pharmaceuticals detected during five years in sampling point R5.	27
Table S18	Concentrations of the pharmaceuticals detected during five years in WWTP Effluent E1.	28
Table S19	Concentrations of the pharmaceuticals detected during five years in WWTP Effluent E2.	29
Table S20	Concentrations of the pharmaceuticals detected during five years in WWTP Influent I1.	30
Table S21	Concentrations of the pharmaceuticals detected during five years in WWTP Influent I2.	31

List of Figures

Figures		Page
Figure S1	Scheme of sampling points in the Lis River (SP1 to SP5) and WWTP influents (WWTP I1 and WWTP I2) and effluents (WWTP E1 and WWTP E2).	32
Figure S2	Solid phase extraction procedure used for the extraction of the studied compound in river water and in wastewaters (effluent and influent) samples.	33
Figure S3	Procedures used for the matrix effect determination in the analyzed matrices.	34
Figure S4	Matrix effect in the river water and WWTP effluent and influent matrices.	35
Figure S5	Method detection limit for the studied compound for river water and WWTP effluent and influent matrices.	36
Figure S6	Risk Quotient obtained in each trophic level: (a) lower risk ($RQ<0.1$), (b) moderate risk ($0.1<RQ<1$), and (c) high risk ($RQ>1$).	37

Table S1. Localization, type of treatment, hydraulic retention time, sludge retention time, average flow rate and equivalent population for WWTP1 and WWTP2 (2013–2019).

Information	WWTP1	WWTP2
**Location from the river mouth**	25 km	5 km
**Inputs**	Domestic and hospital wastewaters	Domestic, hospital effluents, livestock production effluents, and landfill leachate
**Treatments**	Activated sludge with nitrogen removal	Activated sludge with phosphorus removal and tertiary treatment with filtration and UV disinfection
**Hydraulic retention time (h)**	12.5	25.0
**Sludge retention time (d)**	16.4	18.0
**Average flow rate (m^3/d)**	6,250	37,997
**Population equivalent (designed)**	49,351	248,685
**Population equivalent (served)**	21,726	110,131

HRT–Hydraulic retention time; SRT–Sludge retention time.

Table S2. Reagents, eluents, materials, and equipment used in the extraction and in the analysis methodologies.

**Reagents, eluents, materials, and equipment**		**Supplier Company**
Methanol MS grade Hipersolv CHROMANORM®		VWR (Gliwice, Poland)
Acetonitrile MS grade Hipersolv CHROMANORM®		VWR (Fontenay-sous-Bois, France)
Propanol MS grade		Sigma-Aldrich (Steinheim, Germany)
Formic acid (PA-ACS)		Carlo Erba (Rodano, Italy)
Hydrochloric acid (HCl) 37%		Carlo Erba (Rodano, Italy)
Ethylenediaminetetraacetic acid disodium salt 2-hydrate (Na_2EDTA)		Panreac (Barcelona, Spain)
Sodium Hydrochloride		LabKem (Barcelona, Spain)
Ultrapure water (resistivity of 18.2 MΩ.cm) was produced using the Simplicity 185 system		Millipore, Molsheim, France
Stratra-X cartridge (200 mg, 3 mL) used in SPE extraction		Phenomenex, California, USA
PTFE syringe filters (0.22 μm) used to filter sample extracts		Specanalitica, Carcavelos, Portugal
Nylon membrane filter (0.22 μm) used to filter eluents		Fioroni Filters, Ingré, France
Vacuum pump Dinko D-95		Barcelona, Spain
Ultrasonic bath (Sonorex Digital 10P, Bandelin DK 255P) used to degas eluent		Germany
Shimadzu Nexera UHPLC-MS/MS-ESI (LCMS-8030)	Controller (CBM - 20A)	Kyoto, Japan
	Solvent delivery pumps (LC-30 AD) for eluent A	
	Solvent delivery pumps (LC-30 AD) for eluent B	
	Column oven (CTO-20 AC)	
	Auto-sampler (SIL-30 AC)	
	Degasser (DGU-20A 5R)	
	System controller (CBM-20A)	
	Lab Solutions software used for control and data processing (version 5.80, Shimadzu)	

Table S3. Pharmaceuticals, metabolites, degradation product, isotopically labeled internal standards (ILIS), chemical abstracts service (CAS), formula, molecular weight, supplier company, and solvent used for the preparation of each stock solution.

**Pharmaceuticals, metabolites, degradation product, and Isotopically Labeled Internal Standards** Note 1	**CAS** Note 2,3	**Formula (Molecular Weight)** Note 3	**Supplier Company**	**Solvents used for the preparation of each stock solution**
**Acetaminophen**	103-90-2	$C_8H_9NO_2$ (MW=151.165 g/mol)	Sigma-Aldrich (Madrid, Spain)	Acetonitrile
**Acetylsalicylic acid**	50-78-2	$C_9H_8O_4$ (MW= 180.159 g/mol)	Sigma-Aldrich (Madrid, Spain)	Acetonitrile
**Alprazolam**	28981-97-7	$C_{17}H_{13}ClN_4$ (MW= 308.77 g/mol)	Lipomed AG (Arlesheim, Switzerland)	Methanol
**Amfepramone**	134-80-5<br>90-84-6	$C_{13}H_{20}ClNO$ **(Amfepramone hydrochloride)** (MW= 241.76 g/mol)<br>$C_{13}H_{19}NO$ (MW= 205.301 g/mol)	Lipomed AG (Arlesheim, Switzerland)	Methanol
**Amoxicillin**	61336-70-7<br>26787-78-0	$C_{16}H_{25}N_3O_8S$ (**Amoxicillin trihydrate)** (MW= 419.449 g/mol)<br>$C_{16}H_{19}N_3O_5S$ (MW= 365.404 g/mol)	Sigma-Aldrich (Madrid, Spain)	Methanol-Ultrapure water (2:1, v/v)
**Ampicillin**	7177-48-2	$C_{16}H_{19}N_3O_4S$ (MW= 349.5 g/mol)	Sigma-Aldrich (Madrid, Spain)	Acetonitrile-metanol (1:1, v/v)
**Atenolol**	29122-68-7	$C_{14}H_{22}N_2O_3$ (MW= 266.341 g/mol)	Sigma-Aldrich (Madrid, Spain)	Methanol
**Atorvastatin**	344423-98-9<br>134523-00-5	$C_{66}H_{74}CaF_2N_4O_{13}$ (**Atorvastatin calcium trihydrate)** (MW= 1209.408 g/mol)<br>$C_{33}H_{35}FN_2O_5$ (MW= 558.65 g/mol)	Sigma-Aldrich (Madrid, Spain)	Acetonitrile-5% acetic acid in Ultrapure water
**Azithromycin**	83905-01-5	$C_{38}H_{72}N_2O_{12}$ (MW= 748.996 g/mol)	Sigma-Aldrich (Madrid, Spain)	Methanol
**Bupropion**	31677-93-7<br>34911-55-2	$C_{13}H_{19}Cl_2NO$ (**Bupropion hydrochloride)** (MW= 276.201 g/mol)<br>$C_{13}H_{18}ClNO$ (MW= 239.743 g/mol)	Lipomed AG (Arlesheim, Switzerland)	Methanol
**Caffeine**	58-08-2	$C_8H_{10}N_4O_2$ (MW= 194.194 g/mol)	Sigma-Aldrich (Madrid, Spain)	Methanol
**Carbamazepine**	298-46-4	$C_{15}H_{12}N_2O$ (MW= 236.274 g/mol)	Sigma-Aldrich (Madrid, Spain)	Methanol
**Carboxyibuprofen**	15935-54-3	$C_{13}H_{16}O_4$ (MW= 236.267 g/mol)	Sigma-Aldrich (Madrid, Spain)	Acetonitrile
**Chlorpromazine**	69-09-0<br>318.863	$C_{17}H_{20}Cl_2N_2S$ (**Chlorpromazine hydrochloride)** (MW= 355.321 g/mol)<br>$C_{17}H_{19}ClN_2S$ (MW= 318.863 g/mol)	Sigma-Aldrich (Madrid, Spain)	Methanol-Ultrapure water
**Chlortetracycline**	64-72-2<br>57-62-5	$C_{22}H_{24}Cl_2N_2O_8$ (**Chlortetracycline hydrochloride)** (MW= 515.34 g/mol)<br>$C_{22}H_{23}ClN_2O_8$ (MW= 478.882 g/mol)	Sigma-Aldrich (Madrid, Spain)	Acetonitrile-metanol (1:1, v/v)
**Ciprofloxacin**	85721-33-1	$C_{17}H_{18}FN_3O_3$ (MW= 331.347 g/mol)	Sigma-Aldrich (Madrid, Spain)	Ultrapure Water-10% acetic acid in Ultrapure water (1:1, v/v)
**Citalopram**	59729-33-8	$C_{20}H_{21}FN_2O$ (MW= 324.399 g/mol)	Sigma-Aldrich (Madrid, Spain)	Methanol
**Citalopram N-oxide**	62498-71-9<br>917482-45-2	$C_{20}H_{22}ClFN_2O_2$ (**Citalopram N-oxide hydrochloride)** (MW= 376.856 g/mol)<br>$C_{20}H_{21}FN_2O_2$ (MW= 340.398 g/mol)	H. Lundbeck (Copenhagen, Denmark)	Methanol
**Citalopram propionic acid***	**Not Available**	$C_{18}H_{14}FNO_3$ (MW= 311.312 g/mol)	H. Lundbeck (Copenhagen, Denmark)	Methanol
**Clarithromycin**	81103-11-9	$C_{38}H_{69}NO_{13}$ (MW= 747.964 g/mol)	Sigma-Aldrich (Madrid, Spain)	Methanol
**Clavulanate potassium**	61177-45-5	$C_8H_8N_{O5}K$ (MW= 237.25 g/mol)	Sigma-Aldrich (Madrid, Spain)	Methanol.Ultrapure water (1:1,v/v)
**Clobenzorex**	5843-53-8<br>13364-32-4	$C_{16}H_{19}Cl_2N$ (**Clobenzorex hydrochloride)** (MW= 296.235 g/mol)<br>$C_{16}H_{18}ClN$ (MW= 259.777 g/mol)	LGC (Middlesex, UK)	Methanol
***d*-Cathine**	2153-98-2	$C_9H_{14}ClNO$ (***d*-Cathine hydrochloride)** (MW= 187.667 g/mol)	Lipomed AG (Arlesheim, Switzerland)	Methanol
**Desmethylcitalopram**	97743-99-2<br>62498-67-3	$C_{19}H_{20}ClFN_2O$ (**Desmethylcitalopram hydrochloride)** (MW= 346.83 g/mol)<br>$C_{19}H_{19}FN^2O$	H. Lundbeck (Copenhagen, Denmark)	Methanol
**Diazepam**	439-14-5	$C_{16}H_{13}ClN_2O$ (MW= 284.743 g/mol)	Lipomed AG (Arlesheim, Switzerland)	Methanol
**Diclofenac**	15307-79-6<br>15307-86-5	$C_{14}H_{10}Cl_2NNaO_2$ (**Diclofenac sodium salt)** (MW= 318.12 g/mol)<br>$C_{14}H_{11}Cl_2NO_2$	Sigma-Aldrich (Madrid, Spain)	Acetonitrile-metanol (1:1, v/v)
**Didemethylcitalopram**	1189694-81-2<br>62498-69-5	$C_{18}H_{18}ClFN_2O$ (**Didemethylcitalopram hydrochloride)** (MW= 332.803 g/mol)<br>$C_{18}H_{17}FN_2O$ (MW= 296.3 g/mol)	H. Lundbeck (Copenhagen, Denmark)	Methanol
**Diltiazem**	33286-22-5<br>42399-41-7	$C_{22}H_{27}ClN_2O_4S$ (**Diltiazem hydrochloride)** (MW= 450.978 g/mol)<br>$C_{22}H_{26}N_2O_4S$ (MW= 414.5 g/mol)	Sigma-Aldrich (Madrid, Spain)	Methanol
**Doxycycline**	24390-14-5<br>564-25-0	$C_{22}H_{24}N_2O_8$ HCl $0.5H_2O$ $0.5C_2H_6O$ (**Doxycycline hyclate)** (MW= 512.94 g/mol)<br>$C_{22}H_{24}N_2O_8$ (MW= 444.44 g/mol)	Sigma-Aldrich (Madrid, Spain)	Acetonitrile-metanol (1:1, v/v)
**Enrofloxacin**	93106-60-6	$C_{19}H_{22}FN_3O_3$ (MW= 359.401 g/mol)	Sigma-Aldrich (Madrid, Spain)	Ultrapure Water-10% acetic acid in Ultrapure water (1:1, v/v)
**(+)-Ephedrine**	134-71-4<br>134-72-5	$C_{10}H_{16}ClNO$ (**(+)-Ephedrine hydrochloride)** (MW= 201.694 g/mol)<br>$C_{10}H_{15}NO$ (MW= 165.236 g/mol)	Lipomed AG (Arlesheim, Switzerland)	Methanol
**10,11-Epoxycarbamazepine**	36507-30-9	$C_{15}H_{12}N_2O_2$ (MW= 252.273 g/mol)	Sigma-Aldrich (Madrid, Spain)	Methanol
**Erythromycin**	643-22-1	$C_{37}H_{67}NO_{13}$ (MW= 733.937 g/mol)	Sigma-Aldrich (Madrid, Spain)	Methanol

(cont. Table S3)

**Pharmaceuticals, metabolites, degradation product, and Isotopically Labeled Internal Standards** Note 1	CAS Note 2,3	Formula (Molecular Weight) Note 3	Supplier Company	Solvents used for the preparation of each stock solution
**Fenfluramine**	404-82-0<br>404-82-0	$C_{12}H_{17}ClF_3N$ (**Fenfluramine hydrochloride**) (MW= 267.72 g/mol)<br>$C_{12}H_{16}F_3N$ (MW= 231.262 g/mol)	LGC (Middlesex, UK)	Methanol
**Fenofibrate**	49562-28-9	$C_{20}H_{21}ClO_4$ (MW= 360.834 g/mol)	Sigma-Aldrich (Madrid, Spain)	
**Fluoxetine**	56296-78-7<br>54910-89-3	$C_{17}H_{19}ClF_3NO$ (**Fluoxetine hydrochloride**) (MW= 345.79 g/mol)<br>$C_{17}H_{18}F_3NO$ (MW= 309.332 g/mol)	Sigma-Aldrich (Madrid, Spain)	Methanol
**Gemfibrozil**	25812-30-0	$C_{15}H_{22}O_3$ (MW= 250.338 g/mol)	Sigma-Aldrich (Madrid, Spain)	Methanol
**2-Hydroxyibuprofen**	51146-55-5	$C_{13}H_{18}O_3$ (MW= 222.284 g/mol)	Sigma-Aldrich (Madrid, Spain)	Acetonitrile
**Ibuprofen**	15687-27-1	$C_{13}H_{18}O_2$ (MW= 206.285 g/mol)	Sigma-Aldrich (Madrid, Spain)	Acetonitrile
**Ketoprofen**	22071-15-4	$C_{16}H_{14}O_3$ (MW= 254.285 g/mol)	Sigma-Aldrich (Madrid, Spain)	Acetonitrile
**Lansoprazole**	103577-45-3	$C_{16}H_{14}F_3N_3O_2S$ (MW= 369.362 g/mol)	Sigma-Aldrich (Madrid, Spain)	Methanol
**Lomefloxacin**	8079-52-8<br>98079-51-7	$C_{17}H_{20}ClF_2N_3O_3$ (**Lomefloxacin hydrochloride**)(MW= 387.812 g/mol)<br>$C_{17}H_{19}F_2N_3O_3$ (MW= 351.354 g/mol)	Sigma-Aldrich (Madrid, Spain)	Ultrapure Water-10% acetic acid in Ultrapure water (1:1, v/v)
**Lorazepam**	846-49-1	$C_{15}H_{10}Cl_2N_2O_2$ (MW= 321.157 g/mol)	Lipomed AG (Arlesheim, Switzerland)	Methanol
**Mazindol**	22232-71-9	$C_{16}H_{13}ClN_2O$ (MW= 284.743 g/mol)	Lipomed AG (Arlesheim, Switzerland)	Methanol
**Metformin**	1115-70-4<br>657-24-9	$C_4H_{12}ClN_5$ (**Metformin hydrochloride**) (MW= 165.625 g/mol)<br>$C_4H_{11}N_5$ (MW= 129.167 g/mol)	Sigma-Aldrich (Madrid, Spain)	Methanol
**dl-Methamphetamine**	300-42-5<br>7632-10-2	$C_{10}H_{16}ClN$ (**dl-Methamphetamine hydrochloride**) (MW= 185.70 g/mol)<br>$C_{10}H_{15}N$ (MW= 149.237 g/mol)	Lipomed AG (Arlesheim, Switzerland)	Methanol
**Moxifloxacin**	186826-86-8<br>151096-09-2	$C_{21}H_{25}ClFN_3O_4$ (**Moxifloxacin hydrochloride**) (MW= 437.896 g/mol)<br>$C_{21}H_{24}FN_3O_4$ (MW= 401.438 g/mol)	Sigma-Aldrich (Madrid, Spain)	Ultrapure Water-10% acetic acid in Ultrapure water (1:1, v/v)
**Naproxen**	22204-53-1	$C_{14}H_{14}O_3$ (MW= 230.263 g/mol)	Sigma-Aldrich (Madrid, Spain)	Acetonitrile-metanol (1:1,v/v)
**Nimesulide**	51803-78-2	$C_{13}H_{12}N_2O_5S$ (MW= 308.308 g/mol)	Sigma-Aldrich (Madrid, Spain)	Acetonitrile
**dl-Norephedrine**	154-41-6<br>37577-28-9	$C_9H_{14}ClNO$ (**dl-Norephedrine hydrochloride**) (MW= 187.667 g/mol)<br>$C_9H_{13}NO$ (MW= 151.209 g/mol)	Lipomed AG (Arlesheim, Switzerland)	Methanol
**Norfloxacin**	70458-96-7	$C_{16}H_{18}FN_3O_3$ (MW= 319.33 g/mol)	Sigma-Aldrich (Madrid, Spain)	Ultrapure Water-10% acetic acid in Ultrapure water (1:1, v/v)
**Norfluoxetine**	57226-68-3<br>83891-03-6	$C_{16}H_{17}ClF_3NO$ (**Norfluoxetine hydrochloride)** (MW= 331.763 g/mol)<br>$C_{16}H_{16}F_3NO$ (MW= 295.305 g/mol)	Sigma-Aldrich (Madrid, Spain)	Methanol
**Norsertraline**	675126-08-6<br>87857-41-8	$C_{16}H_{16}Cl_3N$ (**Norsertraline hydrochloride** (MW= 328.661 g/mol)<br>$C_{16}H_{15}Cl_2N$ (MW= 292.203 g/mol)	Cerilliant-Certified Reference Materials (Texas, USA)	Purchased as methanolic solution
**O-Desmethylvenlafaxine**	93413-62-8	$C_{16}H_{25}NO_2$ (MW= 263.381 g/mol)	Sigma-Aldrich (Madrid, Spain)	Purchased as methanolic solution
**Ofloxacin**	82419-36-1	$C_{18}H_{20}FN_3O_4$ (MW= 361.373 g/mol)	Sigma-Aldrich (Madrid, Spain)	Ultrapure Water-10% acetic acid in Ultrapure water (1:1, v/v)
**Oxytetracycline**	2058-46-0<br>2058-46-0	$C_{22}H_{25}ClN_2O_9$ (**Oxytetracycline hydrochloride**) (MW= 496.897 g/mol)<br>$C_{22}H_{24}N_2O_9$ (MW= 460.439 g/mol)	Sigma-Aldrich (Madrid, Spain)	Acetonitrile-metanol (1:1, v/v)
**Paroxetine**	78246-49-8<br>110429-35-1	$C_{19}H_{21}ClFNO_3$ (**Paroxetine hydrochloride)** (MW= 365.829 g/mol)<br>$C_{19}H_{20}FNO_3$ (MW= 329.371 g/mol)	Sigma-Aldrich (Madrid, Spain)	Methanol
**Phenolphthalein**	77-09-8	$C_{20}H_{14}O_4$ (MW= 318.328 g/mol)	Sigma-Aldrich (Madrid, Spain)	Methanol
**Phentermine**	1197-21-3<br>122-09-8	$C_{10}H_{16}ClN$ (**Phentermine hydrochloride**) (MW= 185.695 g/mol)<br>$C_{10}H_{15}N$ (MW= 149.237 g/mol)	Lipomed AG (Arlesheim, Switzerland)	Methanol
**Pravastatin**	81131-70-6<br>81093-37-0	$C_{23}H_{35}NaO_7$ (**Pravastatin sodium salt hydrate**) (MW= 446.516 g/mol)<br>$C_{23}H_{36}O_7$ (MW= 424.534 g/mol)	Sigma-Aldrich (Madrid, Spain)	Methanol
**Propranolol**	318-98-9<br>525-66-6	$C_{16}H_{22}ClNO_2$ (**Propranolol hydrochloride)** (MW= 295.807 g/mol)<br>$C_{16}H_{21}NO_2$ (MW= 259.349 g/mol)	Sigma-Aldrich (Madrid, Spain)	Methanol
**Prulifloxacin**	123447-62-1	$C_{21}H_{20}FN_3O_6S$ (MW= 461.464 g/mol)	Toronto Research Chemicals Inc. (Ontario, Canada)	Ultrapure Water-10% acetic acid in Ultrapure water (1:1, v/v)
**Rimonabant**	168273-06-1	$C_{22}H_{21}Cl_3N_4O$ (MW= 463.787 g/mol)	LGC (Middlesex, UK)	Methanol
**Salicylic acid**	69-72-7	$C_7H_6O_3$ (MW= 138.122 g/mol)	Sigma-Aldrich (Madrid, Spain)	Acetonitrile
**Sertraline**	79559-97-0<br>79617-96-2	$C_{17}H_{18}Cl_3N$ (**Sertraline hydrochloride)** (MW= 342.688 g/mol)<br>$C_{17}H_{17}Cl_2N$ (MW= 306.23 g/mol)	Sigma-Aldrich (Madrid, Spain)	Methanol

(cont. Table S3)

**Pharmaceuticals, metabolites, degradation product, and Isotopically Labeled Internal Standards** Note 1	CAS Note 2,3	Formula (Molecular Weight) Note 3	Supplier Company	Solvents used for the preparation of each stock solution
**Sibutramine**	84485-00-7<br>106650-56-0	$C_{17}H_{27}Cl_2N$ (**Sibutramine hydrochloride**) (MW= 316.31 g/mol)<br>$C_{17}H_{26}ClN$ (MW= 279.852 g/mol)	LGC (Middlesex, UK)	Purchased as methanolic solution
**Simvastatin**	79902-63-9	$C_{25}H_{38}O_5$ (MW= 418.574 g/mol)	Sigma-Aldrich (Madrid, Spain)	Methanol
**Sulfadiazine**	68-35-9	$C_{10}H_{10}N_4O_2S$ (MW= 250.276 g/mol)	Sigma-Aldrich (Madrid, Spain)	Methanol+5µL NaOH+5 µL HCL
**Sulfadimethoxine**	122-11-2	$C_{12}H_{14}N_4O_4S$ (MW= 310.328 g/mol)	Sigma-Aldrich (Madrid, Spain)	Methanol
**Sulfamethazine**	57-68-1	$C_{12}H_{14}N_4O_2S$ (MW= 278.33 g/mol)	Sigma-Aldrich (Madrid, Spain)	Methanol
**Sulfamethizole**	144-82-1	$C_9H_{10}N_4O_2S_2$ (MW= 270.325 g/mol)	Sigma-Aldrich (Madrid, Spain)	Acetonitrile-metanol (1:1, v/v)
**Sulfamethoxazole**	723-46-6	$C_{10}H_{11}N_3O_3S$ (MW= 253.276 g/mol)	Sigma-Aldrich (Madrid, Spain)	Methanol
**Sulfamethoxypyridazine**	80-35-3	$C_{11}H_{12}N_4O_3S$ (MW= 280.302 g/mol)	Sigma-Aldrich (Madrid, Spain)	Methanol
**Sulfapyridine**	144-83-2	$C_{11}H_{11}N_3O_2S$ (MW= 249.288 g/mol)	Sigma-Aldrich (Madrid, Spain)	Methanol
**Sulfaquinoxaline**	59-40-5	$C_{14}H_{12}N_4O_2S$ (MW= 300.336 g/mol)	Sigma-Aldrich (Madrid, Spain)	Methanol
**Sulfathiazole**	144-74-1<br>72-14-0	$C_9H_8N_3NaO_2S_2$ (**Sulfathiazole sodium salt**) (MW= 277.292 g/mol)<br>$C_9H_9N_3O_2S_2$ (MW= 255.31 g/mol)	Sigma-Aldrich (Madrid, Spain)	Acetonitrile-metanol (1:1, v/v)
**Synephrine**	94-07-5	$C_9H_{13}NO_2$ (MW= 167.208 g/mol)	LGC (Middlesex, UK)	Methanol
**Tetracycline**	64-75-5<br>64-75-5	$C_{22}H_{25}ClN_2O_8$ (**Tetracycline hydrochloride**) (MW=480.898 g/mol)<br>$C_{22}H_{24}N_2O_8$ (MW= 444.44 g/mol)	Sigma-Aldrich (Madrid, Spain)	Acetonitrile-metanol (1:1, v/v)
**Topiramate**	97240-79-4	$C_{12}H_{21}NO_8S$ (MW= 339.359 g/mol)	Lipomed AG (Arlesheim, Switzerland)	Methanol
**Trazodone**	19666-36-5<br>19794-93-5	$C_{19}H_{23}Cl_2N_5O$ (**Trazodone hydrochloride**) (MW= 408.327 g/mol)<br>$C_{19}H_{22}ClN_5O$ (MW= 371.869 g/mol)	Sigma-Aldrich (Madrid, Spain)	Methanol
**Trimethoprim**	738-70-5	$C_{14}H_{18}N_4O_3$ (MW= 290.323 g/mol)	Sigma-Aldrich (Madrid, Spain)	Methanol
**Venlafaxine**	99300-78-4	$C_{17}H_{28}ClNO_2$ (**Venlafaxine hydrochloride**) (MW= 313.866 g/mol)	Sigma-Aldrich (Madrid, Spain)	Methanol
	93413-69-5	$C_{17}H_{27}NO_2$ (MW= 277.408 g/mol)		
**Zonisamide**	68291-97-4	$C_8H_8N_2O_3S$ (MW= 212.223 g/mol)	LGC (Middlesex, UK)	Methanol

1	Salicylic acid-d4	97781-16-3	$C_9H_4D_4O_4$ (MW= 184.18 g/mol)	Toronto Research Chemicals Inc. (Ontario, Canada)	Acetonitrile
2	Acetaminophen-d4	64315-36-2	$C_8H_5D_4NO_2$ (MW= 155.189 g/mol)	Toronto Research Chemicals Inc. (Ontario, Canada)	Acetonitrile
3	Ibuprofen-d3	121662-14-4	$C_{13}H_{15}D_3O_2$ (MW= 209.30 g/mol)	Sigma-Aldrich (Madrid, Spain)	Acetonitrile
4	Topiramate-d12	1279037-95-4	$C_{12}H_9D_{12}NO_8S$ (MW= 351.44 g/mol)	LGC (Middlesex, UK)	Purchased as methanolic solution
5	Gemfibrozil-d6	1184986-45-5	$C_8H_5D_4NO_2$ (MW= 256.37 g/mol)	Toronto Research Chemicals Inc. (Ontario, Canada)	Methanol
6	dl-Methamphetamine-d5 hydrochloride	60124-88-1	$C_{10}H_{11}ClD_5N$ (MW= 190.74 g/mol)	Lipomed AG (Arlesheim, Switzerland)	Methanol
7	Diazepam-d5	65854-76-4	$C_{16}H_8D_5ClN_2O$ (MW= 289.77 g/mol)	Lipomed AG (Arlesheim, Switzerland)	Purchased as methanolic solution
8	Caffeine $^{13}C_3$	78072-66-9	$^{13}C_3C_5H_{10}N_4O_2$ (MW= 197.17 g/mol)	Sigma-Aldrich (Madrid, Spain)	Purchased as methanolic solution
9	Azithromycin-d3	163921-65-1	$C_{38}H_{69}D_3N_2O_{12}$ (MW= 752.014 g/mol)	Toronto Research Chemicals Inc. (Ontario, Canada)	Methanol
10	Sulfamethoxazole-d4	1020719-86-1	$C_{10}H_7D_4N_3O_3S$ (MW= 257.3 g/mol)	Toronto Research Chemicals Inc. (Ontario, Canada)	Methanol
11	Carbamazepine-d10	132183-78-9	$C_{15}H_2D_{10}N_2O$ (MW= 246.33 g/mol)	Cerilliant-Certified Reference Materials (Texas, USA)	Purchased as methanolic solution
12	Fluoxetine-d5 hydrochloride	1173020-43-3	$C_{17}H_{14}D_5ClF_3NO$ (MW= 350.821 g/mol)	Sigma-Aldrich (Madrid, Spain)	Methanol
13	Venlafaxine-d6	1062606-12-5	$C_{17}H_{22}ClD_6NO_2$ (MW= 319.90 g/mol)	Cerilliant-Certified Reference Materials (Texas, USA)	Purchased as methanolic solution
14	Metformin-(dimethyl-d6) hydrochloride	1185166-01-1	$C_4D_6H_6ClN_5$ (MW= 171.66 g/mol)	Sigma-Aldrich (Madrid, Spain)	Methanol

* The CAS information is not available for the compound citalopram propionic acid.

Note 1: Pharmaceuticals are organized in the table by alphabetic order.

Note 2: Chemical Abstracts Service, CAS (a unique numerical identifier assigned by the Chemical Abstracts Service to every chemical substance described in the open scientific literature).

Note 3: CAS, formula, and molecular weight for all the compounds were obtained on the website of PubChem [26–27].

Table S4. Concentration of the isotopically labeled internal standards (ILIS) in the standards and samples.

**Isotopically Labeled Internal Standards (ILIS)**		**Final Concentration (µg/L)**
1	Salicylic acid-d4	55
2	Acetaminophen-d4	800
3	Ibuprofen-d3	1000
4	Topiramate-d12	150
5	Gemfibrozil-d6	250
6	dl-Methamphetamine-d5 hydrochloride	200
7	Diazepam-d5	200
8	Caffeine $^{13}C_3$	400
9	Azithromycin-d3	900
10	Sulfamethoxazole-d4	400
11	Carbamazepine-d10	100
12	Fluoxetine-d5 hydrochloride	60
13	Venlafaxine-d6	100
14	Metformin-(dimethyl-d6) hydrochloride	50

Table S5. Chromatographic programs and temperature, gas flow, and energy used in the negative and positive ionization modes.

<table>
<tr><th rowspan="2">CHROMATOGRAPHIC program</th><th rowspan="2">ESI Mode</th><th rowspan="2">Chromatographic Column</th><th rowspan="2">Eluents</th><th rowspan="2">Mode of elution</th><th rowspan="2">Source Dependent Parameters</th><th colspan="2">Gas</th></tr>
<tr><th>Argon</th><th>Nitrogen</th></tr>
<tr><td>Program I</td><td>Negative</td><td><u>Column</u>: Kinetex C18 column (2.6 x 150 mm i.d., 1.7 µm particle size) from Phenomenex, Inc. (California, USA).
<u>Flow rate</u>: 0.22 mL/min
<u>Oven temperature</u>=30ºC
<u>Injection volume</u>= 5 µL</td><td><u>Eluent A</u>
Ultra-pure water

<u>Eluent B</u>
Acetonitrile</td><td><table><tr><th>Time (min)</th><th>%Acetonitrile</th></tr><tr><td>0.0</td><td>30.0</td></tr><tr><td>1.0</td><td>35.6</td></tr><tr><td>2.0</td><td>100</td></tr><tr><td>6.0</td><td>100</td></tr><tr><td>6.5</td><td>30.0</td></tr><tr><td>10.5</td><td>30.0</td></tr></table></td><td>NGF=2.6 L/min
DGF=12.5 L/min
IV=5.0 kV
DLT=250ºC
HBT=300ºC</td><td rowspan="4">Argon was used as the collision-induced dissociation gas (CID) at a pressure of 230 kPa and</td><td rowspan="4">Nitrogen was used in nebulizing and drying gas</td></tr>
<tr><td>Program II</td><td>Positive</td><td><u>Column</u>: CortecsTM UPLC® C18+ column (100 x 2.1 mm i.d.; 1.6 µm particle size) from Waters (Milford, Massachusetts, USA)
<u>Flow rate</u>: 0.30 mL/min
<u>Oven temperature</u>=30ºC
<u>Injection volume</u>= 5 µL</td><td><u>Eluent A</u>
0.1% formic acid in ultrapure water

<u>Eluent B</u>
acetonitrile</td><td><table><tr><th>Time (min)</th><th>%Acetonitrile</th></tr><tr><td>0.0</td><td>5.0</td></tr><tr><td>3.0</td><td>100</td></tr><tr><td>3.5</td><td>100</td></tr><tr><td>4.0</td><td>5.0</td></tr><tr><td>7.0</td><td>5.0</td></tr></table></td><td>NGF=2.6 L/min
DGF=15 L/min
IV=5.0 kV
DLT=300ºC
HBT=425ºC</td></tr>
<tr><td>Program III</td><td>Negative</td><td><u>Column</u>: Kinetex C18 column (2.6 x 150 mm i.d., 1.7 µm particle size) from Phenomenex, Inc. (California, USA)
<u>Flow rate</u>: 0.30 mL/min
<u>Oven temperature</u>=30ºC
<u>Injection volume</u>= 5 µL</td><td><u>Eluent A</u>
Ultra-pure water

<u>Eluent B</u>
Acetonitrile</td><td><table><tr><th>Time (min)</th><th>%Acetonitrile</th></tr><tr><td>0.0</td><td>10</td></tr><tr><td>5.5</td><td>100</td></tr><tr><td>6.5</td><td>100</td></tr><tr><td>7.0</td><td>10</td></tr><tr><td>9.0</td><td>10</td></tr></table></td><td>NGF=2.6 L/min
DGF=15 L/min
IV=5.0 kV
DLT=300ºC
HBT=425ºC</td></tr>
<tr><td>Program IV</td><td>Positive</td><td><u>Column</u>: Kinetex C18 column (2.6 x 150 mm i.d., 1.7 µm particle size) from Phenomenex, Inc. (California, USA)
<u>Flow rate</u>: 0.30 mL/min
<u>Oven temperature</u>=30ºC
<u>Injection volume</u>= 5 µL</td><td><u>Eluent A</u>
0.1% formic acid in ultrapure water

<u>Eluent B</u>
acetonitrile</td><td><table><tr><th>Time (min)</th><th>%Acetonitrile</th></tr><tr><td>0.0</td><td>5.0</td></tr><tr><td>1.0</td><td>5.0</td></tr><tr><td>2.0</td><td>100</td></tr><tr><td>7.0</td><td>100</td></tr><tr><td>8.5</td><td>5.0</td></tr><tr><td>11.0</td><td>5.0</td></tr></table></td><td>NGF=2.6 L/min
DGF=15 L/min
IV=5.0 kV
DLT=300ºC
HBT=425ºC</td></tr>
</table>

NGF-Nebulizing gas flow (nitrogen), **DGF**-Drying gas flow (nitrogen), **IV**-Interface voltage, **DLT**-Desolvation line temperature, and **HBT**-Heat block temperature.

Table S6. Therapeutic class, pharmaceuticals, ionization mode, precursor ions, product ions, mass spectrometry conditions, ion ratio, and isotopically labeled internal standards (ILIS) for each pharmaceutical in the study. Legend: P- Chromatographic program.

Therapeutic class	*Pharmaceuticals, metabolites, degradation products, and isotopically labeled internal standards (ILIS)* Note 4	ESI	Precursor (m/z)	Quantitation product *m/z*	*Q1 Pre Bias (V)*	*CE*	*Q3 Pre Bias (V)*	Qualifier Product *m/z*	*Q1 Pre Bias (V)*	*CE*	*Q3 Pre Bias (V)*	Dwell Time (msec)	ILIS	P
**Analgesic**	Acetaminophen	ESI-	150.20	107.15	16.0	20.0	17.0	**Note 5**				25.0	**2**	**PI**
**NSAIDs**	Acetylsalicylic acid	ESI-	179.30	137.15	20.0	10.0	21.0	92.90	19.0	22.0	10.0	25.0	**1**	**PI**
**NSAIDs-metabolite**	Carboxyibuprofen	ESI-	235.20	191.25	25.0	9.0	16.0	72.90	24.0	17.0	11.0	25.0	**3**	**PI**
**NSAIDs**	Diclofenac	ESI-	294.10	250.20	11.0	11.0	14.0	35.05	14.0	24.0	11.0	25.0	**3**	**PI**
**NSAIDs-metabolite**	2-Hydroxyibuprofen	ESI-	221.20	177.30	23.0	8.0	10.0	**Note 5**				25.0	**3**	**PI**
**NSAIDs**	Ibuprofen	ESI-	205.20	161.30	21.0	10.0	23.0	**Note 5**				25.0	**3**	**PI**
	Ketoprofen	ESI-	253.20	209.20	27.0	7.0	12.0	**Note 5**				25.0	**3**	**PI**
	Naproxen	ESI-	229.20	170.00	24.0	16.0	17.0	169.25	25.0	31.0	17.0	25.0	**3**	**PI**
	Nimesulide	ESI-	307.00	229.15	15.0	16.0	17.0	79.05	15.0	26.0	29.0	25.0	**3**	**PI**
**NSAIDs-degradation product**	Salicylic acid	ESI-	137.30	93.10	15.0	17.0	14.0	65.10	15.0	30.0	10.0	25.0	**1**	**PI**
**Antibiotic**	Azithromycin	ESI+	749.30	158.00	-28.0	-45.0	-16.0	116.10	-38.0	-55.0	-11.0	10.0	**9**	**PII**
	Ciprofloxacin	ESI+	332.00	314.05	-17.0	-22.0	-22.0	231.00	-24.0	-39.0	-16.0	10.0	**10**	**PII**
	Clarithromycin	ESI+	748.30	158.05	-28.0	-34.0	-10.0	83.00	-28.0	-54.0	-17.0	10.0	**9**	**PII**
	Enrofloxacin	ESI+	360.00	316.10	-18.0	-21.0	-15.0	342.10	-27.0	-23.0	-24.0	10.0	**10**	**PII**
	Erythromycin	ESI+	734.40	158.00	-28.0	-33.0	-10.0	83.00	-28.0	-55.0	-19.0	10.0	**9**	**PII**
	Lomefloxacin	ESI+	352.00	264.95	-29.0	-25.0	-19.0	333.95	-18.0	-22.0	-24.0	10.0	**10**	**PII**
	Moxifloxacin	ESI+	402.05	384.05	-21.0	-23.0	-26.0	95.95	-15.0	-48.0	-20.0	10.0	**10**	**PII**
	Norfloxacin	ESI+	320.00	302.05	-27.0	-22.0	-21.0	231.00	-26.0	-43.0	-16.0	10.0	**10**	**PII**
	Ofloxacin	ESI+	362.00	318.10	-29.0	-21.0	-15.0	260.95	-29.0	-29.0	-18.0	10.0	**10**	**PII**
	Prulifloxacin	ESI+	462.00	443.95	-17.0	-22.0	-22.0	360.00	-24.0	-32.0	-24.0	10.0	**10**	**PII**
	Sulfadiazine	ESI+	250.90	155.90	-18.0	-18.0	-10.0	91.95	-19.0	-30.0	-19.0	10.0	**10**	**PII**
	Sulfadimethoxine	ESI+	310.90	155.90	-24.0	-23.0	-15.0	91.95	-25.0	-38.0	-18.0	10.0	**10**	**PII**
	Sulfamethazine	ESI+	278.90	185.90	-23.0	-19.0	-12.0	91.95	-22.0	-36.0	-19.0	10.0	**10**	**PII**
	Sulfamethoxazole	ESI+	253.95	155.90	-29.0	-18.0	-16.0	91.95	-20.0	-32.0	-18.0	10.0	**10**	**PII**
	Sulfamethoxypyridazine	ESI+	280.90	155.95	-14.0	-19.0	-10.0	92.00	-14.0	-32.0	-18.0	10.0	**10**	**PII**
	Sulfapyridine	ESI+	249.90	155.95	-18.0	-18.0	-16.0	91.95	-28.0	-31.0	-20.0	10.0	**10**	**PII**
	Trimethoprim	ESI+	291.00	230.00	-23.0	-25.0	-15.0	123.00	-23.0	-27.0	-12.0	10.0	**10**	**PII**
**Pshychiatric drugs**	Carbamazepine	ESI+	236.95	193.95	-18.0	-21.0	-23.0	193.00	-26.0	-36.0	-13.0	10.0	**11**	**PII**
	Citalopram	ESI+	325.05	108.95	-16.0	-29.0	-23.0	261.95	-16.0	-22.0	-18.0	10.0	**12**	**PII**
	Diazepam	ESI+	284.95	153.90	-23.0	-29.0	-10.0	192.95	-23.0	-33.0	-23.0	10.0	**7**	**PII**
**Pshychiatric drugs-metabolite**	10,11-Epoxycarbamazepine	ESI+	253.00	179.95	-21.0	-27.0	-12.0	235.90	-21.0	-12.0	-16.0	10.0	**11**	**PII**
**Pshychiatric drugs**	Fluoxetine	ESI+	309.95	44.00	-25.0	-14.0	-18.0	**Note 5**				10.0	**12**	**PII**
**Pshychiatric drugs -metabolite**	Norfluoxetine	ESI+	296.00	134.0	-23.0	-8.0	-13.0	30.20	-24.0	-15.0	-12.0	10.0	**12**	**PII**
	Norsertraline	ESI+	291.90	274.90	-23.0	-11.0	-19.0	158.95	-22.0	-24.0	-10.0	10.0	**12**	**PII**
**Pshychiatric drugs**	Paroxetine	ESI+	330.00	70.0	-27.0	-34.0	-14.0	44.0	-37.0	-28.0	-14.0	10.0	**12**	**PII**
	Sertraline	ESI+	305.95	158.90	-15.0	-26.0	-15.0	274.95	-25.0	-13.0	-19.0	10.0	**12**	**PII**
	Trazodone	ESI+	372.05	175.95	-19.0	-26.0	-11.0	147.95	-19.0	-40.0	-15.0	10.0	**12**	**PII**
	Venlafaxine	ESI+	278.10	58.00	-20.0	-25.0	-20.0	260.05	-23.0	-14.0	-12.0	10.0	**13**	**PII**
**Antibiotic**	Ampicillin	ESI+	348.00	307.05	-17.0	-10.0	-21.0	**Note 5**				15.0	**12**	**PII**
**Lipid regulator and cholesterol lowering statin drugs**	Atorvastatin	ESI+	559.10	440.05	-20.0	-24.0	-16.0	250.00	-20.0	-47.0	-16.0	15.0	**12**	**PII**
**β-blockers**	Atenolol	ESI+	267.05	144.95	-10.0	-28.0	-15.0	56.00	-22.0	-34.0	-11.0	15.0	**12**	**PII**
**Stimulant**	Caffeine	ESI+	194.95	138.00	-22.0	-21.0	-13.0	42.00	-23.0	-41.0	-16.0	15.0	**8**	**PII**
**Antibiotic**	Chlorocycline	ESI+	479.00	444.00	-24.0	-24.0	-15.0	462.00	-24.0	-20.0	-22.0	15.0	**12**	**PII**
**Antipsychotic drugs**	Chlorpromazine	ESI+	319.00	85.95	-22.0	-21.0	-18.0	58.00	-25.0	-43.0	-12.0	15.0	**12**	**PII**
**Pshychiatric drugs -metabolite**	Desmethylcitalopram	ESI+	352.05	311.00	-29.0	-8.0	-15.0	108.95	-18.0	-34.0	-22.0	15.0	**12**	**PII**
	Didemethylcitalopram	ESI+	297.00	108.95	-25.0	-23.0	-10.0	262.00	-23.0	-15.0	-18.0	15.0	**12**	**PII**
	Citalopram N-oxide	ESI+	341.05	108.95	-17.0	-26.0	-10.0	261.95	-28.0	-19.0	-18.0	15.0	**12**	**PII**
	O-Desmethylvenlafaxine	ESI+	264.00	58.00	-20.0	-25.0	-20.0	246.05	-21.0	-13.0	-17.0	15.0	**13**	**PII**
**Calcium channel blocker**	Diltiazem	ESI+	415.05	178.00	-20.0	-30.0	-20.0	150.00	-13.0	-48.0	-15.0	15.0	**12**	**PII**
**Antibiotic**	Doxycycline	ESI+	445.05	428.05	-17.0	-21.0	-15.0	97.95	-17.0	-47.0	-20.0	15.0	**12**	**PII**
**Fibrate lipid lowering agent**	Fenofibrate	ESI+	361.00	232.855	-29.0	-16.0	-16.0	138.95	-18.0	-35.0	-13.0	15.0	**12**	**PII**
**Proton pump inhibitor**	Lansoprazole	ESI+	370.00	251.90	-19.0	-13.0	-17.0	118.90	-30.0	-17.0	-12.0	15.0	**12**	**PII**

(cont. Table S6)

Therapeutic class	*Pharmaceuticals, metabolites, degradation products, and isotopically labeled internal standards (ILIS)* **Note 4**	**ESI**	**Precursor (m/z)**	**Quantitation product**				**Qualifier Product**				**Dwell Time (msec)**	**ILIS**	**P**
				m/z	*Q1 Pre Bias (V)*	*CE*	*Q3 Pre Bias (V)*	*m/z*	*Q1 Pre Bias (V)*	*CE*	*Q3 Pre Bias (V)*			
**Antidiabetic drugs**	Metformin	ESI+	129.50	70.95	-10.0	-24.0	-14.0	59.95	-10.0	-15.0	-13.0	15.0	**14**	**PII**
**Antibiotic**	Oxytetracycline	ESI+	460.95	425.95	-23.0	-20.0	-15.0	443.05	-17.0	-15.0	-22.0	15.0	**12**	**PII**
**β-blockers**	Propanolol	ESI+	301.00	260.00	-25.0	-8.0	-18.0	**Note 5**				15.0	**12**	**PII**
**Lipid regulator and cholesterol lowering statin drugs**	Simvastatin	ESI+	419.15	199.00	-15.0	-15.0	-13.0	285.00	-16.0	-13.0	-20.0	15.0	**12**	**PII**
**Antibiotic**	Sulfamethizole	ESI+	270.85	155.95	-13.0	-16.0	-19.0	92.00	-21.0	-31.0	-19.0	15.0	**10**	**PII**
	Sulfaquinoxaline	ESI+	300.90	155.95	-25.0	-19.0	-10.0	92.00	-25.0	-37.0	-18.0	15.0	**10**	**PII**
	Sulfathiazole	ESI+	255.85	155.90	-19.0	-17.0	-16.0	92.00	-19.0	-29.0	-18.0	15.0	**10**	**PII**
	Tetracycline	ESI+	445.05	410.00	-13.0	-22.0	-20.0	153.95	-22.0	-29.0	-15.0	15.0	**12**	**PII**
**Antibiotic**	Amoxicillin	ESI-	364.10	223.00	17.0	11.0	12.0	205.95	18.0	18.0	28.0	25.0	**5**	**PIII**
**Pshychiatric drugs- metabolite**	Citalopram propionic acid	ESI-	310.05	266.10	15.0	11.0	11.0	236.20	30.0	18.0	10.0	25.0	**5**	**PIII**
**Lipid regulator and cholesterol lowering statin drugs**	Gemfibrozil	ESI-	249.20	120.95	26.0	13.0	11.0	105.95	27.0	50.0	15.0	25.0	**5**	**PIII**
**Stimulant, anorectic, anxiolytics, laxatives**	Phenolphthalein	ESI-	317.20	93.10	30.0	14.0	10.0	273.20	28.0	18.0	15.0	25.0	**4**	**PIII**
**Lipid regulator and cholesterol lowering statin drugs**	Pravastatin	ESI-	423.15	101.15	21.0	30.0	15.0	58.85	20.0	24.0	17.0	25.0	**5**	**PIII**
**Stimulant, anorectic, anxiolytics, laxatives**	Topiramate	ESI-	338.10	78.05	21.0	31.0	13.0	95.85	16.0	25.0	10.0	25.0	**4**	**PIII**
	Zonisamide	ESI-	211.10	118.95	22.0	15.0	18.0	147.20	22.0	11.0	11.0	25.0	**4**	**PIII**
**Stimulant, anorectic, anxiolytics, laxatives**	Alprazolam	ESI+	308.50	280.90	-25.0	-29.0	-19.0	205.05	-12.0	-43.0	-14.0	15.0	**7**	**PIV**
	Anfepramone	ESI+	205.80	105.00	-24.0	-24.0	-20.0	100.00	-10.0	-25.0	-20.0	15.0	**6**	**PIV**
	Bupropion	ESI+	239.90	184.00	-29.0	-14.0	-20.0	130.95	-27.0	-30.0	-12.0	15.0	**6**	**PIV**
	d-Cathine	ESI+	151.90	134.00	-17.0	-15.0	-13.0	116.95	-17.0	-20.0	-11.0	15.0	**6**	**PIV**
	Clobenzorex	ESI+	260.00	91.00	-17.0	-27.0	-18.0	119.00	-21.0	-17.0	-11.0	15.0	**6**	**PIV**
	(+)-Ephedrine	ESI+	165.90	148.00	-20.0	-15.0	-16.0	116.95	-18.0	-22.0	-11.0	15.0	**6**	**PIV**
	Fenfluramine	ESI+	231.90	159.90	-27.0	-230	-10.0	109.00	-26.0	-48.0	-10.0	15.0	**6**	**PIV**
	Lorazepam	ESI+	320.90	274.85	-26.0	-24.0	-18.0	302.90	-26.0	-17.0	-14.0	15.0	**7**	**PIV**
	Mazindol	ESI+	284.90	44.00	-23.0	-27.0	-17.0	**Note 5**				15.0	**6**	**PIV**
	dl-Methamphetamine	ESI+	149.80	91.05	-11.0	-22.0	-17.0	119.10	-11.0	-17.0	-24.0	15.0	**6**	**PIV**
	dl-Norephedrine	ESI+	151.80	134.10	-10.0	-15.0	-14.0	117.05	-10.0	-21.0	-23.0	15.0	**6**	**PIV**
	Phentermine	ESI+	149.90	90.95	-17.0	-22.0	-20.0	18.05	-17.0	-9.0	-20.0	15.0	**6**	**PIV**
	Rimonabant	ESI+	462.90	362.90	-23.0	-30.0	-25.0	84.00	-23.0	-28.0	-18.0	15.0	**6**	**PIV**
	Sibutramine	ESI+	279.90	124.90	-23.0	-26.0	-13.0	139.00	-19.0	-17.0	-14.0	15.0	**6**	**PIV**
	Synephrine	ESI+	167.90	149.95	-19.0	-12.0	-18.0	90.95	-19.0	-23.0	-19.0	15.0	**6**	**PIV**
**Isotopically labeled internal standards (ILIS)**	**1-Salycilin acid-d4**	**ESI-**	**141.00**	**97.05**	**16.0**	**17.0**	**20.0**	**Note 6**				**25.0**	**-**	**PI**
	**2-Acetaminophen-d4**	**ESI-**	**154.10**	**111.05**	**20.0**	**20.0**	**20.0**	**Note 6**				**25.0**	**-**	**PI**
	**3-Ibuprofen-d3**	**ESI-**	**208.00**	**164.00**	**21.0**	**10.0**	**23.0**	**Note 6**				**25.0**	**-**	**PI**
	**4-Topiramate-d12**	**ESI-**	**350.20**	**77.95**	**21.0**	**31.0**	**13.0**	**Note 6**				**25.0**	**-**	**PIII**
	**5-Gemfibrazil-d6**	**ESI-**	**255.20**	**121.10**	**26.0**	**13.0**	**11.0**	**Note 6**				**25.0**	**-**	**PIII**
	**6-dl-Methamphetamine-d5**	**ESI+**	**154.70**	**92.10**	**-11.0**	**-21.0**	**-17.0**	**Note 6**				**15.0**	**-**	**PIV**
	**7-Diazepam-d5****	**ESI+**	**289.90**	**154.05**	**-23.0**	**-31.0**	**-10.0-**	**Note 6**				**10.0**	**--**	**PII**
	**7-Diazepam-d5*****	**ESI+**	**289.90**	**154.05**	**-23.0**	**-31.0**	**10.0**	**Note 6**				**15.0**	**-**	**PIV**
	**8-Caffeine $^{13}C_3$**	**ESI+**	**197.95**	**140.05**	**-22.0**	**-22.0**	**-14.0**	**Note 6**				**10.0**	**-**	**PII**
	**9-Azithromycin-d3**	**ESI+**	**752.30**	**83.15**	**-38.0**	**-55.0**	**-11.0**	**Note 6**				**10.0**	**-**	**PII**
	**10-Sulfamethoxazole-d4**	**ESI+**	**258.00**	**96.10**	**-29.0**	**-18.0**	**-16.0**	**Note 6**				**10.0**	**-**	**PII**
	**11-Carbamazepine-d10**	**ESI+**	**246.95**	**204.10**	**-18.0**	**-22.0**	**-23.0**	**Note 6**				**10.0**	**-**	**PII**
	**12-Fluoxetine-d5**	**ESI+**	**315.05**	**44.05**	**-25.0**	**-15.0**	**-20.0**	**Note 6**				**10.0**	**-**	**PII**
	**13-Venlafaxine-d6**	**ESI+**	**283.80**	**64.05**	**-20.0**	**-25.0**	**-20.0**	**Note 6**				**10.0**	**-**	**PII**
	**14-Metformin-d6**	**ESI+**	**135.95**	**60.10**	**-10.0**	**-16.0**	**-13.0**	**Note 6**				**10.0**	**-**	**PII**

**Conditions of diazepam-d5 in program II; ***Conditions of diazepam-d5 in program IV.

<u>Note 4</u>: Pharmaceuticals organized in the table by alphabetic order in each chromatographic program.

<u>Note 5</u>: Only one transition could be recorded due to the poor fragmentation of the compound -[43].

<u>Note 6</u>: For the isotopically labeled internal standards only one transition is needed.

Table S7. Retention time and ion ratio obtained for each analyte.

Pharmaceuticals	tr (min)	**Ion Ratio**				
	Average (min)	Average	RSD (%)	n	-20% Ion ratio	+ 20% Ion ratio
Acetaminophen	1.661	**Note 7**				
Acetylsalicylic acid	1.677	**2.03**	4.75	23	**1.62**	**2.43**
Alprazolam	3.599	**1.88**	4.95	14	**1.50**	**2.25**
Amoxicillin	1.281	**2.61**	4.03	18	**2.09**	**3.14**
Ampicillin	2.816	**Note 7**				
Anfepramone	3.277	**1.39**	2.79	28	**1.11**	**1.66**
Astorvatatin	3.538	**1.84**	2.44	24	**1.47**	**2.20**
Atenolol	0.913	**1.05**	5.64	20	**0.840**	**1.26**
Azithromycin	2.777	**2.70**	3.24	19	**2.16**	**3.25**
Bupropion	3.356	**2.16**	3.58	30	**1.73**	**2.59**
Caffeine	2.473	**1.22**	4.96	33	**0.976**	**1.46**
Carbamazepine	3.110	**4.16**	2.16	27	**3.33**	**4.99**
Carboxyibuprofen	2.905	**1.40**	1.95	28	**1.12**	**1.68**
Cathine	3.251	**2.30**	3.47	35	**1.84**	**2.76**
Chlorocycline	2.498	**1.17**	4.58	11	**0.936**	**1.40**
Chlorpromazine	2.821	**1.49**	3.26	29	**1.19**	**1.79**
Ciprofloxacin	2.379	**2.09**	2.73	25	**1.67**	**2.50**
Citalopram	2.655	**3.20**	2.83	31	**2.56**	**3.84**
Citalopram N-oxide	2.676	**1.38**	3.94	27	**1.11**	**1.66**
Citalopram propionic acid	4.286	**2.30**	2.57	28	**1.90**	**2.80**
Clarithromycin	2.777	**3.05**	2.29	25	**2.44**	**3.66**
Clobenzorex	3.449	**1.81**	4.98	25	**1.45**	**2.18**
Desmethylcitalopram	2.621	**2.82**	3.37	25	**2.26**	**3.39**
O-Desmethylvenlafaxine	2.339	**4.01**	2.05	30	**3.21**	**4.81**
Diazepam	3.529	**1.27**	3.95	29	**1.02**	**1.52**
Diclofenac	4.169	**10.2**	3.51	31	**8.14**	**12.2**
Didemethylcitalopram	2.599	**1.60**	3.49	28	**1.30**	**1.90**
Diltiazem	2.654	**3.19**	3.48	30	**2.55**	**3.82**
Dodycycline	2.526	**5.21**	3.65	19	**4.17**	**6.25**
Enrofloxacin	2.406	**1.32**	4.21	22	**1.06**	**1.59**
Ephendrine	3.257	**3.96**	2.19	30	**3.16**	**4.75**
10, 11-Epoxycarbamazepine	2.899	**1.09**	2.03	30	**0.87**	**1.31**
Erythromycin	2.634	**2.69**	3.79	26	**2.15**	**3.23**
Fenfluramine	3.372	**3.24**	1.93	33	**2.59**	**3.89**
Fenofibrate	4.450	**1.15**	3.42	25	**0.920**	**1.38**
Fentermine	3.257	**17.0**	4.17	23	**13.6**	**20.4**
Fluoxetine	2.804	**Note 7**				
Gemfibrozil	5.677	**52.4**	2.07	20	**41.9**	**62.8**
Hydroxyibuprofen	3.012	**Note 7**				
Ibuprofen	4.307	**Note 7**				
Ketoprofen	3.890	**Note 7**				
Lansoprazole	2.838	**2.89**	3.07	23	**2.32**	**3.47**

(cont. Table S7)

Pharmaceuticals	tr (min)	Ion Ratio				
	Average (min)	Average	RSD (%)	n	-20% Ion ratio	+ 20% Ion ratio
Lomefloxacin	2.391	**1.22**	3.95	15	**0.976**	**1.47**
Lorazepam	3.554	**2.24**	2.73	25	**1.79**	**2.69**
Mazindol	3.356	**Note 7**				
Metformin	0.660	**1.21**	2.95	24	**0.968**	**1.45**
dl-Methamphetamine	3.257	**4.90**	2.99	31	**3.92**	**5.88**
Moxifloxacin	2.469	**3.30**	3.41	19	**2.64**	**3.97**
Naproxen	3.948	**1.84**	3.74	32	**1.47**	**2.21**
Nimesulide	4.133	**8.82**	0.97	24	**7.06**	**10.6**
dl-Norephedrine	3.251	**2.10**	2.96	30	**1.68**	**2.52**
Norfloxacin	2.375	**5.84**	2.25	17	**4.67**	**7.01**
Norfluoxetine	2.778	**1.09**	3.90	29	**0.872**	**1.31**
Norsertraline	2.801	**1.39**	3.24	19	**1.11**	**1.67**
Ofloxacin	2.371	**1.29**	4.44	26	**1.03**	**1.55**
Oxytetracycline	2.357	**2.48**	3.10	13	**1.98**	**2.98**
Paroxetine	2.717	**1.39**	2.87	29	**1.11**	**1.67**
Phenolphthalein	4.531	**5.11**	1.25	25	**4.09**	**6.57**
Potassium clavulanate	1,532	**1.32**	6.04	23	**1.05**	**1.58**
Pravastatin	3.535	**1.15**	2.71	23	**0.920**	**1.38**
Propanolol	2.632	**Note 7**				
Prulifloxacin	2.741	**1.18**	3.55	26	**0.944**	**1.41**
Rimonabant	4.337	**4.70**	2.10	30	**3.76**	**5.64**
Salicylic acid	1.601	**9.74**	3.61	23	**7.79**	**11.7**
Sertraline	2.822	**1.41**	2.82	23	**1.13**	**1.69**
Sibutramine	3.721	**2.09**	3.17	27	**1.67**	**2.50**
Simvastatin	4.259	**1.22**	3.33	29	**0.976**	**1.46**
Sulfadiazine	2.554	**1.26**	2.01	24	**1.01**	**1.51**
Sulfadimethoxine	3.008	**2.03**	2.98	25	**1.62**	**2.43**
Sulfamethazine	2.702	**1.43**	3.11	27	**1.15**	**1.72**
Sulfamethizole	2.666	**1.93**	4.46	25	**1.54**	**2.31**
Sulfamethoxazole	2.891	**1.20**	2.96	30	**0.960**	**1.43**
Sulfamethoxypyridazine	2.695	**1.15**	2.73	24	**0.920**	**1.38**
Sulfapyridine	2.581	**1.31**	3.18	28	**1.04**	**1.57**
Sulfaquinoxaline	2.995	**1.56**	3.82	27	**1.25**	**1.88**
Sulfathiazole	2.511	**1.74**	3.92	26	**1.40**	**2.09**
Synephrine	1.294	**4.45**	2.53	28	**3.56**	**5.34**
Tetracycline	2.384	**2.00**	3.59	14	**1.60**	**2.41**
Topiramate	4.229	**5.54**	3.13	18	**4.43**	**6.65**
Trazodone	2.546	**1.22**	2.43	29	**0.976**	**1.47**
Trimethoprim	2.366	**1.06**	3.30	24	**0.848**	**1.28**
Venlafaxine	2.518	**4.84**	1.78	24	**3.87**	**5.81**
Zonisamide	3.733	**1.81**	2.61	27	**1.45**	**2.17**

Pharmaceuticals organized in the table by alphabetic order.
Note 7: Only one transition could be recorded due to their poor fragmentation.

Table S8. Recoveries (average of three fortification levels) obtained in river water, WWTP effluent and WWTP influent matrices.

Pharmaceuticals, metabolites and degradation products	Average recovery (%)		
	River Water	WWTP Effluent	WWTP Influent
Acetaminophen	25.4	46.5	43.2
Acetylsalicylic acid	84.4	78.1	82.4
Alprazolam	93.9	68.7	79.5
Amoxicillin	54.1	58.9	56.8
Ampicillin	81.1	72.3	56.8
Anfepramone	91.8	92.1	99.9
Astorvatatin	44.6	51.1	61.2
Atenolol	15.1	16.1	23.6
Azithromycin	78.7	74.4	92.1
Bupropion	104	104	94.8
Caffeine	104	102	84.2
Carbamazepine	103	100	96.8
Carboxyibuprofen	90.4	64.9	74.1
Cathine	5.63	8.51	10.0
Chlorocycline	68.9	28.9	28.4
Chlorpromazine	72.7	50.7	59.5
Ciprofloxacin	53.2	76.0	54.7
Citalopram	97.1	103	92.6
Citalopram N-oxide	104	101	100
Citalopram propionic acid	94.8	86.3	105
Clarithromycin	84.2	85.4	80.3
Clavulanate potassium	34.2	48.9	45.7
Clobenzorex	93.3	90.5	83.9
Desmethylcitalopram	95.2	91.8	93.8
O-Demethylvenlafaxine	101	90.6	102
Diazepam	98.2	81.2	97.2
Diclofenac	97.4	102	92.9
Didemethylcitalopram	91.5	86.9	81.5
Diltiazem	106	97.4	92.9
Doxycycline	64.7	52.7	57.8
Enrofloxacin	57.8	81.9	69.8
Ephendrine	12.2	21.0	20.5
10,11-Epoxycarbamazepine	76.9	83.0	78.0
Erythromycin	1.89	73.1	77.5
Fenfluramine	99.4	91.7	101
Fenofibrate	75.3	30.8	54.0
Fentermine	47.8	44.9	44.3
Fluoxetine	86.7	93.9	83.1
Gemfibrozil	102	96.0	87.1
Hydroxyibuprofen	87.4	96.4	86.6
Ibuprofen	103	89.8	113
Ketoprofen	103	95.8	86.4
Lansoprazole	7.68	n.d.	n.d.
Lomefloxacin	61.4	82.8	83.6
Lorazepam	92.9	87.2	84.6
Mazindol	103	95.3	89.2
Metformin	0.800	1.04	0.650

(cont. Table S8)

Pharmaceuticals, metabolites and degradation products	Average recovery (%)		
	River Water	WWTP Effluent	WWTP Influent
dl-Methamphetamine	41.3	42.3	42.9
Moxifloxacin	58.3	76.3	82.9
Naproxen	104	101	96.8
Nimesulide	127	86.8	98.6
dl-Norephedrine	5.29	8.90	9.87
Norfloxacin	43.8	80.3	67.8
Norfluoxetine	94.0	114	73.5
Norsertraline	96.9	91.6	88.5
Ofloxacin	51.6	90.3	71.5
Oxytetracycline	69.9	39.9	39.6
Paroxetine	86.1	77.4	85.6
Phenolphthalein	104	85.9	101
Pravastatin	62.8	55.0	69.3
Propanolol	90.4	110	93.2
Prulifloxacin	74.3	94.4	69.4
Ribonabant	99.3	51.1	68.9
Salicylic acid	78.9	91.3	88.4
Sertraline	100	75.3	64.1
Sibutramine	79.3	79.8	77.1
Simvastatin	60.1	46.2	53.9
Sulfadiazine	83.2	73.6	74.3
Sulfadimethoxine	81.0	68.4	66.5
Sulfamethazine	78.6	61.5	61.0
Sulfamethizole	78.4	6.00	65.9
Sulfamethoxazole	76.6	62.7	68.7
Sulfamethoxypyridazine	84.1	77.4	80.3
Sulfapyridine	68.9	70.3	72.7
Sulfaquinoxaline	80.1	2.78	64.9
Sulfathiazole	74.9	4.84	77.8
Synephrine	0.350	3.26	2.71
Tetracycline	62.3	40.2	59.7
Topiramate	102	93.9	102
Trazodone	82.4	95.7	83.6
Trimethoprim	99.3	93.0	96.6
Venlafaxine	107	97.2	99.4
Zonisamide	100	95.6	97.3

Pharmaceuticals were organized by alphabetic order.

River matrix

(i) <u>Level I</u> (0.10 $\mu g_{pharmaceutical}/L_{sample}$): 1 ml of 25 µg/L of fortified concentration using 250 mL of sample;
(ii) <u>Level II</u> (0.20 µgpharmaceutical/Lsample): 1 ml of 50 µg/L of fortified concentration using 250 mL of sample;
(iii) <u>Level III</u> (0.50 µgpharmaceutical/Lsample): 1 ml of 125 µg/L of fortified concentration using 250 mL of sample.

WWTP effluents:

(i) <u>Level I</u> (0.25 $\mu g_{pharmaceutical}/L_{sample}$): 1 ml of 25 µg/L of fortified concentration using 100 mL of sample;
(ii) <u>Level II</u> (0.50 $\mu g_{pharmaceutical}/L_{sample}$): 1 ml of 50 µg/L of fortified concentration using 100 mL of sample;
(iii) <u>Level III</u> (1.25 $\mu g_{pharmaceutical}/L_{sample}$): 1 ml of 125 µg/L of fortified concentration using 100 mL of sample.

WWTP influents

(i) <u>Level I</u> (0.5 $\mu g_{pharmaceutical}/L_{sample}$): 1 ml of 25 µg/L of fortified concentration using 50 mL of sample;
(ii) <u>Level II</u> (1.0 $\mu g_{pharmaceutical}/L_{sample}$): 1 ml of 50 µg/L of fortified concentration using 50 mL of sample;
(iii) <u>Level III</u> (2.5 $\mu g_{pharmaceutical}/L_{sample}$): 1 ml of 125 µg/L of fortified concentration using 50 mL of sample;

Table S9. Pharmaceuticals, metabolites and degradation products detected in each sample and their concentration (ng/L) in the sampling campaign of 2018.

Pharmaceutical	River sample SP1		River sample SP2		River sample SP3		River sample SP4		River sample SP5		WWTP Effluent-E1		WWTP Effluent-E2		WWTP Influent-I1		WWTP Influent-I2	
	Conc (ng/L)	RSD (%)	Conc (ng/L)	RSD (%)	Conc (ng/L)	RSD (%)	Conc (ng/L)	RSD (%)	Conc (ng/L)	RSD (%)	Conc (ng/L)	RSD (%)	Conc (ng/L)	RSD (%)	Conc (ng/L)	RSD (%)	Conc (ng/L)	RSD (%)
Carbamazepine	8.50	4.0	n.d.		166	5.6	41.7	13	266	8.0	1337	7.6	858	1.7	1048	3.7	652	1.0
10,11-Epoxycarbamazepine	n.d.		n.d.		n.d.		n.d.		n.d.		86.9	12	n.d.		79.9	15	56.1	4.8
Citalopram	n.d.		n.d.		14.4	8.9	n.d.		41.5	13	158	3.2	158	0.60	131	13	144	12
Citalopram propionic acid	n.d.		9.30	1.2	19.4	0.74	11.1	5.3	24.0	2.8	124	2.1	89.6	18	61.2	0.69	52.3	13
Desmethylcitalopram	n.d.		n.d.		n.d.		n.d.		n.d.		n.d.		n.d.		198	10	n.d.	
Didemethylcitalopram	n.d.		n.d.		n.d.		n.d.		n.d.		61.6	0.083	n.d.		n.d.		n.d.	
Diazepam	n.d.		n.d.		n.d.		n.d.		13.9	1.5	n.d.		n.d.		n.d.		73.2	7.8
Fluoxetine	n.d.		<MDL		<MDL		<MDL		<MDL		13.5	37	5.72	25	<MDL		<MDL	
Paroxetine	n.d.		n.d.		n.d.		n.d.		n.d.		17.8	8.3	n.d.		n.d.		n.d.	
Sertraline	n.d.		n.d.		n.d.		n.d.		8.74	10	84.9	1.7	50.8	11	121	1.2	106	2.7
Trazodone	n.d.		n.d.		2.15	11	n.d.		35.2	19	111	2.0	126	9.1	29.1	13	115	8.5
Venlafaxine	6.19	27	3.85	15	45.6	2.1	9.34	6.9	124	6.5	370	4.7	488	7.3	367	3.9	408	4.4
Bupropion	n.d.		n.d.		27.1	1.6	15.7	7.2	60.6	2.2	169	8.5	205	0.064	178	1.1	201	11
Azithromycin	n.d.		n.d.		187	8.4	n.d.		532	7.4	4399	2.8	4370	3.1	652	14	1096	9.1
Clarithromycin	n.d.		n.d.		99.1	9.8	23.0	5.1	187	2.6	2566	2.2	1291	1.2	2214	8.6	1690	5.8
Ciprofloxacin	n.d.		n.d.		n.d.		n.d.		n.d.		580	13	482	15	939	10	1814	1.1
Ofloxacin	n.d.		n.d.		n.d.		n.d.		n.d.		1037	11	382	18	1071	4.0	954	16
Trimethoprim	n.d.		n.d.		n.d.		n.d.		38.1	19	41.9	10	<MDL		<MDL		<MDL	
Sulfamethoxazole	n.d.		n.d.		n.d.		n.d.		n.d.		97.7	9.0	67.5	16	945	2.6	917	14
Sulfapyridine	n.d.		n.d.		11.6	20	n.d.		n.d.		n.d.		n.d.		696	5.9	955	16
Oxytetracycline	n.d.		n.d.		n.d.		n.d.		n.d.		n.d.		8685	19	n.d.		n.d.	
Tetracycline	n.d.		n.d.		55.1	18	n.d.		n.d.		n.d.		n.d.		n.d.		n.d.	
Alprazolam	n.d.		n.d.		n.d.		n.d.		n.d.		78.7	11	104	10	n.d.		n.d.	
Lorazepam	n.d.		n.d.		n.d.		n.d.		n.d.		656	7.5	n.d.		n.d.		n.d.	
Acetaminophen	<MDL		77.6	7.3	20.6	7.9	142	9.7	93.8	18	194	14	182	5.3	96770	2.6	82702	7.2
Acetylsalicylic acid	n.d.		n.d.		n.d.		n.d.		n.d.		84.7	6.8	n.d.		99.5	4.7	115	4.0
Salicylic acid	106	16	348	13	180	17	118	21	205	4.2	215	3.4	271	15	15719	5.8	29012	20
Ibuprofen	1.38	29	4.78	3.8	69.1	0.63	45.5	2.8	94.7	8.1	79.5	14	290	6.3	5862	2.5	12490	15
Carboxyibuprofen	n.d.		n.d.		1277	2.8	n.d.		n.d.		n.d.		n.d.		392986	0.069	639403	12
Hydroxyibuprofen	15.3	0.59	36.2	7.2	1673	1.5	388	0.65	1295	4.9	3035	0.42	2336	18	169633	7.3	283651	8.7
Diclofenac	n.d.		n.d.		285	3.4	13.1	1.9	112	1.6	1606	0.95	1648	18	1939	6.8	3316	11
Ketoprofen	<MDL		<MDL		10.1	0.37	<MDL		42.4	15	140	4.7	222	1.6	66.3	15	564	15
Naproxen	n.d.		n.d.		156	2.0	12.3	12	28.3	6.2	534	2.8	95.4	6.0	2225	7.1	3004	8.5
Gemfibrozil	n.d.		5.72	12	15.2	14	9.46	23	39.9	3.1	80.9	2.5	141	6.0	109	5.4	189	3.1
Simvastatin	n.d.		n.d.		n.d.		n.d.		n.d.		<MDL		n.d.		n.d.		n.d.	
Pravastatin	n.d.		n.d.		n.d.		n.d.		n.d.		235	0.081	n.d.		n.d.		n.d.	
Phenolphthalein	n.d.		n.d.		<MDL		<MDL		<MDL		n.d.		n.d.		n.d.		n.d.	
Ephendrine	n.d.		n.d.		n.d.		n.d.		n.d.		n.d.		n.d.		4786	1.1	5816	4.4
Fentermine	n.d.		n.d.		n.d.		n.d.		n.d.		n.d.		n.d.		n.d.		193	7.7
Topiramate	n.d.		0.66	6.9	77.4	2.9	23.4	3.6	192	0.58	730	3.7	985	1.7	n.d.		1074	0.40
dl-Methamphetamine	n.d.		n.d.		n.d.		n.d.		n.d.		n.d.		n.d.		8.2	14	n.d.	
Caffeine	57.9	12	482	12	3202	1.8	628	4.6	474	5.8	19312	0.80	844	18	53251	14	67203	4.7
Atenolol	n.d.		n.d.		n.d.		n.d.		n.d.		1835	8.4	n.d.		2432	11	3012	9.2
Diltiazem	n.d.		n.d.		<MDL		<MDL		<MDL		<MDL		<MDL		<MDL		<MDL	
Fenofibrate	n.d.		n.d.		n.d.		n.d.		n.d.		n.d.		<MDL		n.d.		356	4.7

Table S10. Pharmaceuticals, metabolites and degradation products detected in each sample and their concentration (ng/L) in the sampling campaign of 2019.

Pharmaceutical	River sample SP1		River sample SP2		River sample SP3		River sample SP4		River sample SP5		WWTP Effluent-E1		WWTP Effluent-E2		WWTP Influent-I1		WWTP Influent-I2	
	Conc (ng/L)	RSD (%)	Conc (ng/L)	RSD (%)	Conc (ng/L)	RSD (%)	Conc (ng/L)	RSD (%)	Conc (ng/L)	RSD (%)	Conc (ng/L)	RSD (%)	Conc (ng/L)	RSD (%)	Conc (ng/L)	RSD (%)	Conc (ng/L)	RSD (%)
Carbamazepine	0.593	4.0	2.43	16	45.7	5.6	21.8	13	185	8.0	834	7.6	639	1.7	733	3.7	633	1.0
10,11-Epoxycarbamazepine	n.d.		n.d.		n.d.		n.d.		n.d.		57.0	12	n.d.		n.d.		n.d.	
Citalopram	n.d.		n.d.		6.64	8.9	<MDL		46.2	13	15	3.2	153	0.60	71.6	13	81.2	12
Citalopram propionic acid	n.d.		n.d.		n.d.		n.d.		n.d.		134	2.1	28.1	18	n.d.		n.d.	
Desmethylcitalopram	n.d.		n.d.		n.d.		n.d.		n.d.		185	5.1	n.d.		n.d.		n.d.	
Fluoxetine	5.79	13	6.42	2.8	8.53	13	7.19	13	21.1	10	66.8	37	63.2	25	82.1	15	81.8	6.2
Paroxetine	n.d.		n.d.		n.d.		n.d.		n.d.		112	8.3	n.d.				n.d.	
Sertraline	n.d.		n.d.		12.5	12	9.34	3.0	21.4	10	89.7	1.7	87.8	11	224	1.2	171	2.7
Trazodone	n.d.		n.d.		34.0	11	15.8	3.7	148	19	344	2.0	362	9.1	298	13	414	8.5
Venlafaxine	n.d.		n.d.		8.26	2.1	n.d.		n.d.		n.d.		n.d.		397	3.9	n.d.	
Bupropion	n.d.		n.d.		n.d.		<MDL		28.5	2.2	60.6	8.5	77.9	0.064	52.2	1.1	147	11
Azithromycin	n.d.		n.d.		73.0	8.4	6.20	5.7	41.7	7.4	210	2.8	34.3	3.1	139	14	15.1	9.1
Ciprofloxacin	n.d.		n.d.		n.d.		n.d.		n.d.		257	13	n.d.		264	10	378	1.1
Clarithromycin	n.d.		n.d.		69.4	9.8	9.73	5.1	31.6	2.6	166	2.2	16.6	1.2	158	8.6	12.5	5.8
Ofloxacin	n.d.		n.d.		<MDL		<MDL		n.d.		110	11	41.7	18	<MDL		39.9	16
Sulfadiazine	114	9.3	n.d.		n.d.		n.d.		n.d.		n.d.		<MDL		n.d.		20.6	5.7
Sulfamethazine	n.d.		n.d.		n.d.		4.87	13	n.d.		n.d.		n.d.		n.d.		n.d.	
Sulfamethoxazole	n.d.		n.d.		n.d.		6.96	12	22.1	1.8	n.d.		n.d.		179	2.6	291	14
Sulfapyridine	n.d.		n.d.		n.d.		15.2	14	n.d.		n.d.		n.d.		245	5.9	353	16
Trimethoprim	<MDL		n.d.		n.d.		n.d.		80.6	19	215	10	236	17	236	16	349	15
Tetracycline	n.d.		n.d.		n.d.		n.d.		n.d.		n.d.		332	3.6	n.d.		n.d.	
Chlorocycline	n.d.		n.d.		n.d.		n.d.		n.d.		n.d.		1006	1.6	n.d.		n.d.	
Acetaminophen	35.2	11	44.3	7.3	51.7	7.9	43.7	9.7	148	18	70.8	14	494	5.3	3094	2.6	31965	7.2
Carboxyibuprofen	n.d.		n.d.		43.8	2.8	109	6.3	n.d.		n.d.		n.d.		14282	0.069	21480	12
Diclofenac	n.d.		n.d.		119	3.4	72.5	1.9	848	1.6	2272	0.95	2799	18	2178	6.8	3372	11
Hydroxyibuprofen	49.0	0.59	65.4	7.2	480	1.5	300	0.65	226	4.9	10002	0.42	299	18	11364	7.3	11273	8.7
Ibuprofen	3.41	29	8.88	3.8	57.8	0.63	43.1	2.8	35.9	8.1	2457	13.6	163	6.3	4655	2.5	8229	15
Ketoprofen	17.5	7.0	18.8	3.2	28.6	0.37	71.7	0.71	61.3	15	22	4.7	179	1.6	384	15	552	15
Naproxen	n.d.		n.d.		50.5	2.0	40.9	12	130	6.2	900	2.8	492	6.0	3126	7.1	1696	8.5
Nimesulide	n.d.		6.50	15	n.d.		n.d.		n.d.		n.d.		n.d.		n.d.		n.d.	
Salicylic acid	54.6	16	74.9	13	75.4	17	85.0	21	125	4.2	978	3.4	142	15	4612	5.8	32733	20
Gemfibrozil	n.d.		n.d.		n.d.		n.d.		25.8	3.1	82.0	2.5	78.9	6.0	n.d.		n.d.	
Astorvatatin	n.d.		n.d.		68.3	2.7	n.d.		n.d.		686	0.22	298	4.4	507	3.8	n.d.	
Fenfluramine	n.d.		n.d.		n.d.		n.d.		n.d.		20.9	4.2	n.d.		n.d.		n.d.	
dl-Methamphetamine	n.d.		n.d.		n.d.		n.d.		n.d.		n.d.		<MDL		n.d.		n.d.	
Phenolphthalein	n.d.		n.d.		n.d.		n.d.		n.d.		n.d.		n.d.		406	4.4	n.d.	
Topiramate	n.d.		n.d.		35.8	2.9	24.5	3.6	237	0.58	894	3.7	858	1.7	n.d.		548	0.40
Caffeine	76.8	12	65.9	12	151	1.8	399	4.6	145	5.8	836	0.80	n.d.		35104	14	52959	4.7
Atenolol	n.d.		n.d.		n.d.		n.d.				1215	8.4	n.d.		3171	11	2368	9.2
Diltiazem	n.d.		n.d.		n.d.		<MDL		25.6	14	24.8	15.0	n.d.		28.4	12	n.d.	

Table S11. Number of compounds, metabolites, degradation products, therapeutical classes, detection frequency, and concentration in µg/L of the detected analytes in the sampling campaign performed in 2018.

SC	Observations	River water	WWTP Effluents	WWTP influents
2018	**Analytes**<br>(Gray shadow-compounds that appear in all matrices)	**27compounds**<br>Carbamazepine; Azithromycin; Diclofenac<br>Citalopram; Clarithromycin; Ketoprofen<br>Citalopram propionic acid; Sulfapyridine; Naproxen<br>Diazepam; Trimethoprim; Salicylic acid<br>Fluoxetine; Tetracycline; Caffeine<br>Sertraline; Acetaminophen; Phenolphthalein<br>Trazodone; Carboxyibuprofen; Topiramate<br>Venlafaxine; Hydroxyibuprofen; Diltiazem<br>Bupropion; Ibuprofen; Gemfibrozil	**36 compounds**<br>Carbamazepine; Clarithromycin; Ibuprofen<br>10,11-Epoxycarbamazepine; Ciprofloxacin; Ketoprofen<br>Citalopram; Ofloxacin; Naproxen<br>Citalopram propionic acid; Trimethoprim; Salicylic acid<br>Didemethylcitalopram; Sulfamethoxazole; Gemfibrozil<br>Fluoxetine; Oxytetracycline; Pravastatin<br>Paroxetine; Alprazolam; Simvastatin<br>Sertraline; Lorazepam; Topiramate<br>Trazodone; Acetaminophen; Caffeine<br>Venlafaxine; Acetylsalicylic acid; Atenolol<br>Bupropion; Diclofenac; Diltiazem<br>Azithromycin; Hydroxyibuprofen; Fenofibrate	**36 compounds**<br>Carbamazepine; Clarithromycin; Ketoprofen<br>10,11-Epoxycarbamazepine; Ciprofloxacin; Naproxen<br>Citalopram; Ofloxacin; Salicylic acid<br>Citalopram propionic acid; Trimethoprim; Gemfibrozil<br>Desmethylcitalopram; Sulfamethoxazole; Topiramate<br>Diazepam; Sulfapyridine; Caffeine<br>Fluoxetine; Acetaminophen; Ephendrine<br>Sertraline; Acetylsalicylic acid; Fentermine<br>Trazodone; Carboxyibuprofen; dl-Methamphetamine<br>Venlafaxine; Diclofenac; Atenolol<br>Bupropion; Hydroxyibuprofen; Diltiazem<br>Azithromycin; Ibuprofen; Fenofibrate
	**Metabolites**	Citalopram propionic acid	Citalopram propionic acid	Citalopram propionic acid
		Hydroxyibuprofen	Hydroxyibuprofen	Hydroxyibuprofen
		Carboxyibuprofen	--	Carboxyibuprofen
		--	10,11 Epoxycarbamazepine	10,11 Epoxycarbamazepine
		--	Didemethylcitalopram	--
		--	--	DesmethylCitalopram
	**Degradation product**	Salicylic acid	Salicylic acid	Salicylic acid
	**Therapeutic classes**	Psychiatric drugs	Psychiatric drugs	Psychiatric drugs
		Antibiotics	Antibiotics	Antibiotics
		NSAIDs	NSAIDS	NSAIDS
		Calcium channel blocker	Calcium channel blocker	Calcium channel blocker
		Lipid regulator and cholesterol lowering statin drugs	Lipid regulator and cholesterol lowering statin drugs	Lipid regulator and cholesterol lowering statin drugs
		Stimulant, anorectic, anxiolytics, laxatives	Stimulant, anorectic, anxiolytics, and laxatives	Stimulant, anorectic, anxiolytics, and laxatives
			B-blockers	B-blockers
			Fibrate lipid lowering agent	Fibrate lipid lowering agent
	**Therapeutic classes of the most detected compounds**	**With 22 compounds:**	**With 28 compounds:**	**With 27 compounds:**
		NSAIDs	NSAIDs	NSAIDs
		Antibiotics	Antibiotics	Antibiotics
		Psychiatric drugs	Psychiatric drugs	Psychiatric drugs
	**Detection frequency (100%)**	**6 compounds:**	**26 compounds:**	**30 compounds:**
		Venlafaxine	Venlafaxine	Venlafaxine
		Caffeine	Caffeine	Caffeine
		Hydroxyibuprofen	Hydroxyibuprofen	Hydroxyibuprofen
		Ibuprofen	Ibuprofen	Ibuprofen
		Salicylic acid	Salicylic acid	Salicylic acid
		Ketoprofen	Ketoprofen	Ketoprofen
		--	Carbamazepine	Carbamazepine
		--	Citalopram	Citalopram
		--	Citalopram propionic acid	Citalopram propionic acid
		--	Fluoxetine	Fluoxetine
		--	Sertraline	Sertraline
		--	Trazodone	Trazodone
		--	Bupropion	Bupropion
		--	Azithromycin	Azithromycin
		--	Clarithromycin	Clarithromycin
		--	Ciprofloxacin	Ciprofloxacin
		--	Ofloxacin	Ofloxacin
		--	Trimethoprim	Trimethoprim

(cont. Table S11)

SC	Observations	River water	WWTP Effluents	WWTP influents
		--	Sulfamethoxazole	Sulfamethoxazole
		--	Acetaminophen	Acetaminophen
		--	Diclofenac	Diclofenac
		--	Naproxen	Naproxen
		--	Gemfibrozil	Gemfibrozil
		--	Diltiazem	Diltiazem
		--	Alprazolam	--
		--	Topiramate	--
		--		10,11-Epoxycarbamazepine
		--		Sulfapyridine
		--		Carboxyibuprofen
		--		Ephendrine
		--		Acetylsalicylic acid
		--		Atenolol
	**Concentration (µg/L)**	Hydroxyibuprofen (SP3 and SP5)	Hydroxyibuprofen (E1 and E2)	Hydroxyibuprofen (I1 and I2)
		Caffeine (SP3)	Caffeine (E1 and E2)	Caffeine (I1 and I2)
		Carboxyibuprofen (SP3)	--	Carboxyibuprofen (I1 and I2)
		--	Carbamazepine (E1)	Carbamazepine (I1)
		--	Azithromycin (E1 and E2)	Azithromycin (I1 and I2)
		--	Clarithromycin (E1 and E2)	Clarithromycin (I1 and I2)
		--	Ofloxacin (E1)	Ofloxacin (I1)
		--	Diclofenac (E1 and E2)	Diclofenac (I1 and I2)
		--	Atenolol (E1)	Atenolol (I1 and I2)
		--	Oxytetracycline (E2)	--
		--	--	Ciprofloxacin (I2)
		--	--	Acetaminophen (I1 and I2)
		--	--	Ibuprofen (I1 and I2)
		--	--	Naproxen (I1 and I2)
		--	--	Salicylic acid (I1 and I2)
		--	--	Ephedrine (I1 and I2)
		--	--	Topiramate (I1)
	**Concentration < MDL**	Diltiazem (SP3 to SP5)	Diltiazem (E1 and E2)	Diltiazem (I1 and I2)
		Fluoxetine (SP2 to SP5)	--	Fluoxetine (I1 and I2)
		Acetaminophen (SP1)	--	--
		Ketoprofen (SP1, SP2, and SP4)	--	--
		Phenolphthalein (SP3 to SP5)	--	Trimethoprim (I1 and I2)
		--	Trimethoprim (E2)	--
		--	Simvastatin (E1)	--
		--	--	Fenofibrate (I2)
	**Analytes never detected**	**37 compounds**<br>Amoxicillin, ampicillin, anfepramone, astorvatatin, cathine, chlorocycline, chlorpromazine, clobenzorex, citalopram n-oxide, doxycycline, enrofloxacin, erythromycin, fenfluramine, lansoprazole, lomefloxacin, mazindol, metformin, moxifloxacin, nimesulide, dl-norephedrine, norfloxacin, norfluoxetine, norsertraline, o-demethylvenlafaxine, potassium clavulanate, propanolol, prulifloxacin, ribonabant, sibutramine, sulfadiazine, sulfadimethoxine, sulfamethazine, sulfamethizole, sulfamethoxypyridazine, sulfaquinoxaline, sulfathiazole, synephrine, zonisamide		

Table S12. Number of compounds, metabolites, degradation products, therapeutical classes, detection frequency, and concentration in μg/L of the detected analytes in the sampling campaign performed in 2019.

SC	Observations	River water	WWTP Effluents	WWTP influents
2019	**Analytes** (Gray shadow-compounds that appear in all matrices)	**29 compounds**	**33 compounds**	**29 compounds**

River water (29 compounds):

Carbamazepine	Sulfadiazine	Ketoprofen
Citalopram	Sulfamethazine	Naproxen
Fluoxetine	Sulfamethoxazole	Nimesulide
Sertraline	Sulfapyridine	Salicylic acid
Trazodone	Trimethoprim	Gemfibrozil
Venlafaxine	Acetaminophen	Astorvatatin
Bupropion	Carboxyibuprofen	Topiramate
Azithromycin	Diclofenac	Caffeine
Clarithromycin	Hydroxyibuprofen	Diltiazem
Ofloxacin	Ibuprofen	

WWTP Effluents (33 compounds):

Carbamazepine	Ciprofloxacin	Ketoprofen
10,11-Epoxycarbamazepine	Clarithromycin	Naproxen
Citalopram	Ofloxacin	Salicylic acid
Citalopram propionic acid	Sulfadiazine	Gemfibrozil
Desmethylcitalopram	Trimethoprim	Astorvatatin
Fluoxetine	Tetracycline	Fenfluramine
Paroxetine	Chlorocycline	dl-Methamphetamine
Sertraline	Acetaminophen	Topiramate
Trazodone	Diclofenac	Caffeine
Bupropion	Hydroxyibuprofen	Atenolol
Azithromycin	Ibuprofen	Diltiazem

WWTP influents (29 compounds):

Carbamazepine	Ofloxacin	Ketoprofen
Citalopram	Sulfadiazine	Naproxen
Fluoxetine	Sulfamethoxazole	Salicylic acid
Sertraline	Sulfapyridine	Astorvatatin
Trazodone	Trimethoprim	Phenolphthalein
Venlafaxine	Acetaminophen	Topiramate
Bupropion	Carboxyibuprofen	Caffeine
Azithromycin	Diclofenac	Atenolol
Ciprofloxacin	Hydroxyibuprofen	Diltiazem
Clarithromycin	Ibuprofen	

SC	Observations	River water	WWTP Effluents	WWTP influents
	**Detected metabolites**	Hydroxyibuprofen	Hydroxyibuprofen	Hydroxyibuprofen
		Carboxyibuprofen	--	Carboxyibuprofen
		--	10,11-Epoxycarbamazepine	--
		--	Citalopram propionic acid	--
		--	Desmethylcitalopram	--
	**Degradation product**	Salicylic acid	Salicylic acid	Salicylic acid
	**Therapeutic classes**	Psychiatric drugs	Psychiatric drugs	Psychiatric drugs
		Antibiotics	Antibiotics	Antibiotics
		Nsaids	Nsaids	Nsaids
		Calcium channel blocker	Calcium channel blocker	Calcium channel blocker
		Lipid regulator and cholesterol lowering statin drugs	Lipid regulator and cholesterol lowering statin drugs	--
		Stimulant, anorectic, anxiolytics, laxatives	Stimulant, anorectic, anxiolytics, and laxatives	Stimulant, anorectic, anxiolytics, and laxatives
			B-blockers	B-blockers
	**Therapeutic classes of the most detected compounds**	**With 24 compounds**	**With 25 compounds**	**With 23 compounds**
		Nsaids	Nsaids	Nsaids
		Antibiotics	Antibiotics	Antibiotics
		Psychiatric drugs	Psychiatric drugs	Psychiatric drugs
	**Detection frequency (100 %)**	**Observed for 8 compounds:**	**Observed for 21 compounds:**	**Observed for 23 compounds:**
		Carbamazepine	Carbamazepine	Carbamazepine
		Fluoxetine	Fluoxetine	Fluoxetine
		Acetaminophen	Acetaminophen	Acetaminophen
		Hydroxyibuprofen	Hydroxyibuprofen	Hydroxyibuprofen
		Ibuprofen	Ibuprofen	Ibuprofen
		Ketoprofen	Ketoprofen	Ketoprofen
		Salicylic acid	Salicylic acid	Salicylic acid
		Caffeine	--	Caffeine
		--	Citalopram	Citalopram
		--	Citalopram propionic acid	--
		--	Sertraline	Sertraline
		--	Trazodone	Trazodone
		--	Bupropion	Bupropion
		--	Azithromycin	Azithromycin
		--	Clarithromycin	Clarithromycin
		--	Ofloxacin	Ofloxacin
		--	Trimethoprim	Trimethoprim
		--	Diclofenac	Diclofenac
		--	Naproxen	Naproxen
		--	Gemfibrozil	--
		--	Astorvatatin	--

(cont. Table S12)

SC	Observations	River water	WWTP Effluents	WWTP influents
		--	Topiramate	--
		--	--	Ciprofloxacin
		--	--	Sulfamethoxazole
		--	--	Sulfapyridine
		--	--	Carboxyibuprofen
		--	--	Atenolol
	**Concentration (µg/L)**	--	Hydroxyibuprofen (E1)	Hydroxyibuprofen (I1 and I2)
		--	Diclofenac (E1, E2, I1, and I2)	Diclofenac (E1, E2, I1, and I2)
		--	Ibuprofen (I1 and I2)	Ibuprofen (I1 and I2)
		--	Atenolol (E1)	Atenolol (I1 and I2)
		--	Chlorocycline (E2)	--
		--	--	Carboxyibuprofen (I1 and I2)
		--	--	Acetaminophen (I1 and I2)
		--	--	Naproxen (I1 and I2)
		--	--	Salicylic acid (I1 and I2)
		--	--	Caffeine (I1 and I2)
	**Concentration < MDL**	Citalopram (SP4)	--	--
		Bupropion (SP4)	--	--
		Ofloxacin (SP3 and SP4)	--	--
		Trimethoprim (SP1)	--	--
		Diltiazem (SP4)	--	--
		--	Sulfadiazine (E2)	--
		--	dl-Methamphetamine(E2)	--
		--	--	Ofloxacin (I1)
	**Compounds never detected**	**43 compounds:** Acetylsalicylic acid, alprazolam, amfepramone, amoxicillin, ampicillin, cathine, chlorpromazine, didemethylcitalopram, citalopraml N-oxide, clavulanate potassium, clobenzorex, O-desmethylvenlafaxine, Diazepam, dodycycline, Enrofloxacin, ephendrine, Erythromycin, fenofibrate, fentermine, lansoprazole, lomefloxacin, lorazepam, mazindol, metformin, moxifloxacin, dl-norephedrine, Norfloxacin, Norfluoxetine, Norsertraline, oxytetracycline, pravastatin, propranolol, prulifloxacin, ribonabant, sibutramine, simvastatin, sulfadimethoxine, sulfamethizole, sulfamethoxypyridazine, sulfaquinoxaline, sulfathiazole, synephrine, zonisamide		

Table S13. Concentrations of the pharmaceuticals detected during five years in sampling point R1.

**R1**	Concentration (ng/L)													
Pharmaceutical	**Aug2013***	**Sep2013***	**Oct2013***	**Nov2013***	**Jan2014***	**Febr2014***	**March2014***	**April2014***	**May2014***	**Jun2014***	**Jul2014***	**Jun2017**	**Febr2018****	**May2019****
Carbamazepine	34.8	33.6	29.0	32.9	25.5	27.7	25.2	25.8	25.9	36.5	30.4	Not Performed	8.50	0.593
Citalopram	n.d.	n.d.	n.d.	n.d.	n.d.	n.d.	n.d.	n.d.	n.d.	n.d.	n.d.	Not Performed	n.d.	n.d.
Diazepam	n.d.	n.d.	n.d.	n.d.	n.d.	n.d.	n.d.	n.d.	n.d.	n.d.	n.d.	Not Performed	n.d.	n.d.
10,11-Epoxycarbamazepine	n.d.	n.d.	33.2	n.d.	n.d.	33.8	n.d.	n.d.	n.d.	n.d.	n.d.	Not Performed	n.d.	n.d.
Fluoxetine	2.60	2.48	2.17	3.32	10.0	2.26	6.04	2.52	2.01	2.18	2.34	Not Performed	n.d.	5.79
Norfluoxetine	n.d.	n.d.	n.d.	n.d.	n.d.	n.d.	n.d.	n.d.	n.d.	n.d.	n.d.	Not Performed	n.d.	n.d.
Paroxetine	n.d.	n.d.	n.d.	n.d.	n.d.	n.d.	n.d.	n.d.	n.d.	n.d.	n.d.	Not Performed	n.d.	n.d.
Sertraline	<MDL	<MDL	n.d.	<MDL	<MDL	n.d.	n.d.	<MDL	n.d.	<MDL	n.d.	Not Performed	n.d.	n.d.
Trazodone	n.d.	n.d.	n.d.	n.d.	n.d.	n.d.	n.d.	n.d.	n.d.	n.d.	n.d.	Not Performed	n.d.	n.d.
Venlafaxine	n.d.	<MDL	<MDL	n.d.	n.d.	n.d.	n.d.	n.d.	n.d.	n.d.	n.d.	Not Performed	6.19	n.d.
Azithromycin	n.d.	n.d.	n.d.	n.d.	n.d.	n.d.	16.2	n.d.	n.d.	n.d.	n.d.	Not Performed	n.d.	n.d.
Ciprofloxacin	n.d.	n.d.	n.d.	n.d.	n.d.	n.d.	n.d.	n.d.	n.d.	n.d.	n.d.	Not Performed	n.d.	n.d.
Clarithromycin	n.d.	n.d.	n.d.	n.d.	n.d.	n.d.	n.d.	n.d.	n.d.	n.d.	n.d.	Not Performed	n.d.	n.d.
Enrofloxacin	n.d.	n.d.	n.d.	n.d.	n.d.	n.d.	n.d.	n.d.	n.d.	n.d.	n.d.	Not Performed	n.d.	n.d.
Ofloxacin	n.d.	n.d.	n.d.	n.d.	n.d.	n.d.	n.d.	n.d.	n.d.	n.d.	n.d.	Not Performed	n.d.	n.d.
Sulfadiazine	n.d.	n.d.	n.d.	n.d.	n.d.	n.d.	n.d.	n.d.	n.d.	n.d.	n.d.	Not Performed	n.d.	114
Sulfadimethoxine	n.d.	n.d.	n.d.	n.d.	n.d.	n.d.	n.d.	n.d.	n.d.	n.d.	n.d.	Not Performed	n.d.	n.d.
Sulfamethazine	n.d.	n.d.	n.d.	n.d.	n.d.	n.d.	n.d.	n.d.	n.d.	n.d.	n.d.	Not Performed	n.d.	n.d.
Sulfamethoxazole	n.d.	n.d.	n.d.	n.d.	n.d.	n.d.	n.d.	n.d.	n.d.	n.d.	n.d.	Not Performed	n.d.	n.d.
Sulfamethoxypyridazine	n.d.	n.d.	n.d.	n.d.	n.d.	n.d.	n.d.	n.d.	n.d.	n.d.	n.d.	Not Performed	n.d.	n.d.
sulfapyridine	n.d.	n.d.	n.d.	n.d.	n.d.	n.d.	n.d.	n.d.	n.d.	n.d.	n.d.	Not Performed	n.d.	n.d.
Trimethoprim	n.d.	n.d.	n.d.	n.d.	n.d.	n.d.	n.d.	n.d.	n.d.	n.d.	n.d.	Not Performed	n.d.	<MDL
Acetaminophen	33.6	47.0	<MDL	<MDL	<MDL	<MDL	<MDL	<MDL	<MDL	<MDL	<MDL	Not Performed	<MDL	35.2
Acetylsalicylic acid	n.d.	n.d.	n.d.	n.d.	n.d.	n.d.	n.d.	n.d.	n.d.	n.d.	n.d.	Not Performed	n.d.	n.d.
Carboxyibuprofen	n.d.	n.d.	n.d.	n.d.	n.d.	n.d.	n.d.	n.d.	n.d.	n.d.	n.d.	Not Performed	n.d.	n.d.
Diclofenac	n.d.	n.d.	n.d.	n.d.	n.d.	n.d.	n.d.	n.d.	n.d.	n.d.	n.d.	Not Performed	n.d.	n.d.
Hydroxyibuprofen	81.4	318	<MDL	<MDL	24.4	n.d.	<MDL	n.d.	n.d.	<MDL	18.9	Not Performed	15.3	49.0
Ibuprofen	<MDL	<MDL	<MDL	<MDL	<MDL	<MDL	<MDL	<MDL	<MDL	<MDL	<MDL	Not Performed	1.4	3.4
Ketoprofen	<MDL	52.1	<MDL	<MDL	<MDL	<MDL	<MDL	<MDL	<MDL	<MDL	<MDL	Not Performed	<MDL	17.5
Naproxen	n.d.	n.d.	n.d.	n.d.	n.d.	n.d.	n.d.	n.d.	n.d.	n.d.	n.d.	Not Performed	n.d.	n.d.
Nimesulide	n.d.	n.d.	n.d.	n.d.	n.d.	n.d.	n.d.	n.d.	n.d.	n.d.	n.d.	Not Performed	n.d.	n.d.
Salicylic acid	43.1	78.5	95.4	89.2	35.6	68.8	52.0	36.8	64.8	53.8	53.6	Not Performed	106.0	54.6
**SUM**	**196**	**531**	**160**	**125**	**95.5**	**133**	**99.4**	**65.1**	**92.6**	**92.5**	**105**		**137**	**280**

* Results presented in the study of Paíga, P., Santos, L.H.M.L.M., Ramos, S., Jorge, S., Silva, J.G., and Delerue-Matos, C. Presence of pharmaceuticals in the Lis River (Portugal): Sources, fate and seasonal variation. *Sci Total Environ* **2016,** 573, 164-177.

** Present study

Table S14. Concentrations of the pharmaceuticals detected during five years in sampling point R2.

**R2**	Concentration (ng/L)													
Pharmaceutical	**Aug2013***	**Sep2013***	**Oct2013***	**Nov2013***	**Jan2014***	**Febr2014***	**March2014***	**April2014***	**May2014***	**Jun2014***	**Jul2014***	**Jun2017**	**Febr2018****	**May2019****
Carbamazepine	30.9	33.2	30.3	36.9	25.2	27.5	24.9	25.1	25.2	36.3	25.9	Not Performed	n.d.	2.43
Citalopram	n.d.	n.d.	n.d.	n.d.	n.d.	< MDL	17.8	< MDL	n.d.	n.d.	n.d.	Not Performed	n.d.	n.d.
Diazepam	n.d.	n.d.	n.d.	n.d.	n.d.	n.d.	n.d.	n.d.	n.d.	n.d.	n.d.	Not Performed	n.d.	n.d.
10,11-Epoxycarbamazepine	n.d.	n.d.	n.d.	n.d.	n.d.	33.5	n.d.	n.d.	n.d.	n.d.	n.d.	Not Performed	n.d.	n.d.
Fluoxetine	3.04	2.44	2.25	3.11	5.34	3.00	19.5	3.08	2.07	2.15	2.29	Not Performed	<MDL	6.42
Norfluoxetine	n.d.	n.d.	n.d.	n.d.	n.d.	n.d.	n.d.	n.d.	n.d.	n.d.	n.d.	Not Performed	n.d.	n.d.
Paroxetine	n.d.	n.d.	n.d.	25.6	n.d.	n.d.	n.d.	n.d.	n.d.	n.d.	n.d.	Not Performed	n.d.	n.d.
Sertraline	n.d.	n.d.	n.d.	<MDL	n.d.	n.d.	n.d.	n.d.	n.d.	n.d.	n.d.	Not Performed	n.d.	n.d.
Trazodone	n.d.	n.d.	n.d.	n.d.	n.d.	n.d.	n.d.	n.d.	n.d.	n.d.	n.d.	Not Performed	n.d.	n.d.
Venlafaxine	n.d.	n.d.	n.d.	n.d.	n.d.	n.d.	n.d.	n.d.	n.d.	n.d.	n.d.	Not Performed	3.85	n.d.
Azithromycin	n.d.	n.d.	n.d.	n.d.	n.d.	n.d.	15.0	n.d.	n.d.	n.d.	n.d.	Not Performed	n.d.	n.d.
Ciprofloxacin	n.d.	n.d.	n.d.	n.d.	n.d.	n.d.	n.d.	n.d.	n.d.	n.d.	n.d.	Not Performed	n.d.	n.d.
Clarithromycin	n.d.	n.d.	n.d.	n.d.	n.d.	n.d.	26.8	n.d.	n.d.	n.d.	n.d.	Not Performed	n.d.	n.d.
Enrofloxacin	n.d.	n.d.	n.d.	n.d.	n.d.	n.d.	n.d.	n.d.	n.d.	n.d.	n.d.	Not Performed	n.d.	n.d.
Ofloxacin	n.d.	n.d.	n.d.	n.d.	n.d.	n.d.	n.d.	n.d.	n.d.	n.d.	n.d.	Not Performed	n.d.	n.d.
Sulfadiazine	n.d.	n.d.	n.d.	n.d.	n.d.	n.d.	n.d.	n.d.	n.d.	n.d.	n.d.	Not Performed	n.d.	n.d.
Sulfadimethoxine	n.d.	n.d.	n.d.	n.d.	n.d.	n.d.	n.d.	n.d.	n.d.	n.d.	n.d.	Not Performed	n.d.	n.d.
Sulfamethazine	25.7	n.d.	n.d.	45.4	36.3	30.0	n.d.	n.d.	n.d.	n.d.	n.d.	Not Performed	n.d.	n.d.
Sulfamethoxazole	n.d.	n.d.	n.d.	n.d.	n.d.	n.d.	n.d.	n.d.	n.d.	n.d.	n.d.	Not Performed	n.d.	n.d.
Sulfamethoxypyridazine	n.d.	n.d.	n.d.	n.d.	n.d.	n.d.	n.d.	n.d.	n.d.	n.d.	n.d.	Not Performed	n.d.	n.d.
sulfapyridine	n.d.	n.d.	n.d.	n.d.	n.d.	n.d.	n.d.	n.d.	n.d.	n.d.	n.d.	Not Performed	n.d.	n.d.
Trimethoprim	n.d.	n.d.	n.d.	n.d.	n.d.	n.d.	n.d.	n.d.	n.d.	n.d.	n.d.	Not Performed	n.d.	n.d.
Acetaminophen	n.d.	<MDL	<MDL	33.6	59.2	22.7	38.3	<MDL	<MDL	<MDL	<MDL	Not Performed	77.6	44.3
Acetylsalicylic acid	n.d.	n.d.	n.d.	n.d.	n.d.	n.d.	n.d.	n.d.	n.d.	n.d.	n.d.	Not Performed	n.d.	n.d.
Carboxyibuprofen	n.d.	n.d.	n.d.	n.d.	n.d.	n.d.	n.d.	n.d.	n.d.	n.d.	n.d.	Not Performed	n.d.	n.d.
Diclofenac	n.d.	n.d.	n.d.	n.d.	n.d.	n.d.	n.d.	n.d.	n.d.	n.d.	n.d.	Not Performed	n.d.	n.d.
Hydroxyibuprofen	142	28.6	18.2	n.d.	n.d.	n.d.	n.d.	87.4	60.5	41.0	98.9	Not Performed	36.2	65.4
Ibuprofen	<MDL	62.3	<MDL	35.2	<MDL	53.7	169	<MDL	124	144	129	Not Performed	4.78	8.88
Ketoprofen	<MDL	<MDL	<MDL	<MDL	<MDL	<MDL	<MDL	<MDL	<MDL	<MDL	<MDL	Not Performed	<MDL	18.8
Naproxen	n.d.	<MDL	<MDL	<MDL	<MDL	n.d.	n.d.	n.d.	n.d.	<MDL	n.d.	Not Performed	n.d.	n.d.
Nimesulide	n.d.	n.d.	n.d.	n.d.	n.d.	n.d.	n.d.	n.d.	n.d.	n.d.	n.d.	Not Performed	n.d.	6.50
Salicylic acid	137	72.2	95.1	165	90.0	49.3	91.5	69.1	43.9	49.6	25.0	Not Performed	348	74.9
**SUM**	**338**	**199**	**146**	**345**	**216**	**220**	**401**	**185**	**255**	**273**	**281**		**470**	**228**

* Results presented in the study of Paíga, P., Santos, L.H.M.L.M., Ramos, S., Jorge, S., Silva, J.G., and Delerue-Matos, C. Presence of pharmaceuticals in the Lis River (Portugal): Sources, fate and seasonal variation. *Sci Total Environ* **2016,** 573, 164-177.

** Present study.

Table S15. Concentrations of the pharmaceuticals detected during five years in sampling point R3.

**R3**	Concentration (ng/L)													
Pharmaceutical	**Aug2013***	**Sep2013***	**Oct2013***	**Nov2013***	**Jan2014***	**Febr2014***	**March2014***	**April2014***	**May2014***	**Jun2014***	**Jul2014***	**Jun2017**	**Febr2018****	**May2019****
Carbamazepine	214	173	34.4	46.8	29.1	32.9	27.8	39.9	39.7	68.9	51.2	Not Performed	166	45.7
Citalopram	n.d.	n.d.	n.d.	n.d.	n.d.	n.d.	n.d.	n.d.	n.d.	1.67	n.d.	Not Performed	14.4	6.64
Diazepam	n.d.	n.d.	n.d.	n.d.	n.d.	n.d.	n.d.	n.d.	n.d.	n.d.	n.d.	Not Performed	n.d.	n.d.
10,11-Epoxycarbamazepine	n.d.	n.d.	n.d.	n.d.	n.d.	34.1	n.d.	n.d.	n.d.	n.d.	n.d.	Not Performed	n.d.	n.d.
Fluoxetine	15.9	8.25	3.74	5.76	8.20	3.25	7.64	5.63	3.57	6.52	5.58	Not Performed	<MDL	8.5
Norfluoxetine	n.d.	n.d.	n.d.	n.d.	n.d.	n.d.	n.d.	n.d.	n.d.	n.d.	n.d.	Not Performed	n.d.	n.d.
Paroxetine	n.d.	n.d.	n.d.	n.d.	n.d.	n.d.	n.d.	n.d.	n.d.	n.d.	n.d.	Not Performed	n.d.	n.d.
Sertraline	n.d.	n.d.	<MDL	n.d.	<MDL	n.d.	<MDL	n.d.	<MDL	n.d.	n.d.	Not Performed	n.d.	12.5
Trazodone	27.6	5.42	n.d.	< MDL	n.d.	n.d.	n.d.	n.d.	n.d.	<MDL	<MDL	Not Performed	2.15	34.0
Venlafaxine	92.0	65.0	n.d.	n.d.	n.d.	n.d.	n.d.	n.d.	n.d.	n.d.	n.d.	Not Performed	45.6	8.3
Azithromycin	n.d.	n.d.	n.d.	<MDL	n.d.	n.d.	11.1	n.d.	<MDL	n.d.	<MDL	Not Performed	187	73.0
Ciprofloxacin	88.7	n.d.	n.d.	n.d.	n.d.	n.d.	n.d.	n.d.	n.d.	n.d.	n.d.	Not Performed	n.d.	n.d.
Clarithromycin	n.d.	n.d.	<MDL	<MDL	n.d.	n.d.	21.9	n.d.	n.d.	n.d.	n.d.	Not Performed	99.1	69.4
Enrofloxacin	n.d.	n.d.	n.d.	n.d.	n.d.	n.d.	n.d.	n.d.	n.d.	n.d.	n.d.	Not Performed	n.d.	n.d.
Ofloxacin	n.d.	n.d.	n.d.	n.d.	n.d.	n.d.	n.d.	n.d.	n.d.	n.d.	n.d.	Not Performed	n.d.	< MDL
Sulfadiazine	n.d.	n.d.	n.d.	n.d.	n.d.	n.d.	n.d.	n.d.	n.d.	n.d.	n.d.	Not Performed	n.d.	n.d.
Sulfadimethoxine	n.d.	n.d.	n.d.	n.d.	n.d.	n.d.	n.d.	n.d.	n.d.	n.d.	n.d.	Not Performed	n.d.	n.d.
Sulfamethazine	n.d.	n.d.	n.d.	n.d.	n.d.	n.d.	n.d.	n.d.	n.d.	n.d.	n.d.	Not Performed	n.d.	n.d.
Sulfamethoxazole	n.d.	n.d.	n.d.	n.d.	n.d.	n.d.	n.d.	n.d.	n.d.	n.d.	n.d.	Not Performed	n.d.	n.d.
Sulfamethoxypyridazine	n.d.	n.d.	n.d.	n.d.	n.d.	n.d.	n.d.	n.d.	n.d.	n.d.	n.d.	Not Performed	n.d.	n.d.
sulfapyridine	n.d.	n.d.	n.d.	n.d.	n.d.	n.d.	n.d.	n.d.	n.d.	n.d.	n.d.	Not Performed	11.6	n.d.
Trimethoprim	n.d.	n.d.	n.d.	n.d.	n.d.	n.d.	n.d.	n.d.	n.d.	n.d.	n.d.	Not Performed	n.d.	n.d.
Acetaminophen	133	43.6	4.9	35.1	19.0	<MDL	<MDL	19.7	<MDL	74.0	<MDL	Not Performed	20.6	51.7
Acetylsalicylic acid	n.d.	n.d.	n.d.	n.d.	n.d.	n.d.	n.d.	n.d.	n.d.	n.d.	n.d.	Not Performed	n.d.	n.d.
Carboxyibuprofen	n.d.	n.d.	n.d.	n.d.	n.d.	n.d.	n.d.	n.d.	n.d.	n.d.	n.d.	Not Performed	1277	43.8
Diclofenac	n.d.	n.d.	n.d.	n.d.	n.d.	n.d.	n.d.	n.d.	n.d.	n.d.	n.d.	Not Performed	285	119
Hydroxyibuprofen	n.d.	n.d.	n.d.	n.d.	n.d.	n.d.	n.d.	n.d.	n.d.	n.d.	16.3	Not Performed	1673	480
Ibuprofen	635	196	<MDL	360	77.9	129	426	83.1	139	238	<MDL	Not Performed	69.1	57.8
Ketoprofen	<MDL	75.3	<MDL	8.22	<MDL	<MDL	<MDL	19.6	<MDL	<MDL	<MDL	Not Performed	10.1	28.6
Naproxen	176	260	<MDL	<MDL	<MDL	<MDL	<MDL	72.7	<MDL	<MDL	<MDL	Not Performed	156	50.5
Nimesulide	n.d.	n.d.	n.d.	n.d.	n.d.	n.d.	n.d.	n.d.	n.d.	n.d.	n.d.	Not Performed	n.d.	n.d.
Salicylic acid	232	198	128	110	46.1	46.6	85.1	61.3	40.9	81.2	32.8	Not Performed	180	75.4
**SUM**	**1614**	**1025**	**171**	**566**	**180**	**246**	**580**	**302**	**217**	**470**	**106**		**4195**	**1165**

* Results presented in the study of Paíga, P., Santos, L.H.M.L.M., Ramos, S., Jorge, S., Silva, J.G., and Delerue-Matos, C. Presence of pharmaceuticals in the Lis River (Portugal): Sources, fate and seasonal variation. *Sci Total Environ* **2016,** 573, 164-177.

** Present study.

Table S16. Concentrations of the pharmaceuticals detected during five years in sampling point R4.

**R4**	Concentration (ng/L)													
Pharmaceutical	**Aug2013***	**Sep2013***	**Oct2013***	**Nov2013***	**Jan2014***	**Febr2014***	**March2014***	**April2014***	**May2014***	**Jun2014***	**Jul2014***	**Jun2017**	**Febr2018****	**May2019****
Carbamazepine	64.4	45.1	27.7	29.7	27.2	27.1	29.6	30.8	31.7	36.8	32.1	Not Performed	41.7	21.8
Citalopram	n.d.	n.d.	n.d.	n.d.	n.d.	n.d.	n.d.	n.d.	n.d.	n.d.	n.d.	Not Performed	n.d.	< MDL
Diazepam	n.d.	n.d.	n.d.	n.d.	n.d.	n.d.	n.d.	n.d.	n.d.	n.d.	n.d.	Not Performed	n.d.	n.d.
10,11-Epoxycarbamazepine	n.d.	n.d.	n.d.	n.d.	n.d.	n.d.	n.d.	n.d.	34.3	n.d.	n.d.	Not Performed	n.d.	n.d.
Fluoxetine	4.00	2.57	2.92	2.38	3.45	2.83	4.04	2.72	2.67	3.63	2.53	Not Performed	<MDL	7.19
Norfluoxetine	n.d.	n.d.	n.d.	n.d.	n.d.	n.d.	n.d.	n.d.	n.d.	n.d.	n.d.	Not Performed	n.d.	n.d.
Paroxetine	n.d.	n.d.	n.d.	n.d.	25.5	n.d.	n.d.	n.d.	n.d.	n.d.	n.d.	Not Performed	n.d.	n.d.
Sertraline	n.d.	n.d.	<MDL	<MDL	n.d.	<MDL	n.d.	n.d.	n.d.	n.d.	n.d.	Not Performed	n.d.	9.34
Trazodone	MDL	n.d.	n.d.	n.d.	n.d.	n.d.	n.d.	n.d.	<MDL	n.d.	n.d.	Not Performed	n.d.	15.8
Venlafaxine	66.7	n.d.	n.d.	n.d.	n.d.	n.d.	n.d.	n.d.	n.d.	n.d.	<MDL	Not Performed	9.34	n.d.
Azithromycin	n.d.	n.d.	n.d.	n.d.	n.d.	n.d.	<MDL	n.d.	<MDL	n.d.	n.d.	Not Performed	n.d.	6.20
Ciprofloxacin	n.d.	n.d.	n.d.	n.d.	n.d.	n.d.	n.d.	n.d.	n.d.	n.d.	n.d.	Not Performed	n.d.	n.d.
Clarithromycin	n.d.	n.d.	<MDL	n.d.	n.d.	n.d.	<MDL	n.d.	n.d.	n.d.	n.d.	Not Performed	23.0	9.73
Enrofloxacin	n.d.	n.d.	n.d.	n.d.	n.d.	n.d.	n.d.	n.d.	n.d.	n.d.	n.d.	Not Performed	n.d.	n.d.
Ofloxacin	n.d.	n.d.	n.d.	n.d.	n.d.	n.d.	n.d.	n.d.	n.d.	n.d.	n.d.	Not Performed	n.d.	MDL
Sulfadiazine	n.d.	n.d.	n.d.	n.d.	n.d.	n.d.	n.d.	n.d.	n.d.	n.d.	n.d.	Not Performed	n.d.	n.d.
Sulfadimethoxine	n.d.	n.d.	n.d.	n.d.	n.d.	n.d.	n.d.	n.d.	n.d.	n.d.	n.d.	Not Performed	n.d.	n.d.
Sulfamethazine	<MDL	<MDL	<MDL	<MDL	<MDL	<MDL	<MDL	<MDL	67.4	<MDL	<MDL	Not Performed	n.d.	4.87
Sulfamethoxazole	n.d.	n.d.	n.d.	n.d.	n.d.	n.d.	n.d.	n.d.	n.d.	n.d.	n.d.	Not Performed	n.d.	6.96
Sulfamethoxypyridazine	n.d.	n.d.	n.d.	n.d.	n.d.	n.d.	n.d.	n.d.	n.d.	n.d.	n.d.	Not Performed	n.d.	n.d.
sulfapyridine	n.d.	n.d.	n.d.	n.d.	n.d.	n.d.	n.d.	n.d.	n.d.	n.d.	n.d.	Not Performed	n.d.	15.2
Trimethoprim	n.d.	n.d.	n.d.	n.d.	n.d.	n.d.	n.d.	n.d.	n.d.	n.d.	n.d.	Not Performed	n.d.	n.d.
Acetaminophen	49.0	170	22.3	<MDL	527	48.0	99.0	76.6	72.3	120	71.2	Not Performed	141.9	43.7
Acetylsalicylic acid	n.d.	n.d.	n.d.	n.d.	n.d.	n.d.	n.d.	n.d.	n.d.	n.d.	n.d.	Not Performed	n.d.	n.d.
Carboxyibuprofen	n.d.	n.d.	n.d.	n.d.	n.d.	n.d.	n.d.	n.d.	n.d.	n.d.	n.d.	Not Performed	n.d.	109
Diclofenac	n.d.	n.d.	n.d.	n.d.	n.d.	n.d.	n.d.	n.d.	n.d.	n.d.	n.d.	Not Performed	13.1	72.5
Hydroxyibuprofen	<MDL	<MDL	<MDL	<MDL	n.d.	n.d.	<MDL	<MDL	<MDL	n.d.	n.d.	Not Performed	388	300
Ibuprofen	156	<MDL	<MDL	81.5	1317	801	985	476	352	416	491	Not Performed	45.5	43.1
Ketoprofen	<MDL	<MDL	<MDL	<MDL	25.4	<MDL	<MDL	<MDL	<MDL	<MDL	<MDL	Not Performed	<MDL	71.7
Naproxen	<MDL	n.d.	n.d.	n.d.	n.d.	n.d.	n.d.	n.d.	<MDL	<MDL	n.d.	Not Performed	12.3	40.9
Nimesulide	n.d.	n.d.	n.d.	n.d.	n.d.	n.d.	n.d.	n.d.	n.d.	n.d.	n.d.	Not Performed	n.d.	n.d.
Salicylic acid	209	89.1	114	91.9	107	190	97.6	77.6	86.1	130	87.2	Not Performed	118	85.0
**SUM**	**549**	**306**	**166**	**205**	**2032**	**1068**	**1215**	**664**	**646**	**707**	**684**		**792**	**863**

* Results presented in the study of Paíga, P., Santos, L.H.M.L.M., Ramos, S., Jorge, S., Silva, J.G., and Delerue-Matos, C. Presence of pharmaceuticals in the Lis River (Portugal): Sources, fate and seasonal variation. *Sci Total Environ* **2016,** 573, 164-177.

** Present study.

Table S17. Concentrations of the pharmaceuticals detected during five years in sampling point R5.

**R5**	Concentration (ng/L)												
Pharmaceutical	**Aug2013***	**Sep2013***	**Oct2013***	**Nov2013***	**Jan2014***	**Febr2014***	**March2014***	**April2014***	**May2014***	**Jun2014***	**Jul2014***	**Jun2017**	**Febr2018****	**May2019****
Carbamazepine	37.3	112.8	52.7	142.8	27.1	29.7	30.1	41.5	42.2	50.3	30.7	Not Performed	266	185
Citalopram	n.d.	n.d.	n.d.	28.9	n.d.	< MDL	n.d.	n.d.	n.d.	n.d.	n.d.	Not Performed	41.5	46.2
Diazepam	n.d.	n.d.	n.d.	n.d.	n.d.	n.d.	n.d.	n.d.	n.d.	n.d.	n.d.	Not Performed	13.9	n.d.
10,11-Epoxycarbamazepine	n.d.	n.d.	36.3	40.4	n.d.	33.3	n.d.	n.d.	n.d.	33.7	n.d.	Not Performed	n.d.	n.d.
Fluoxetine	2.46	7.10	5.81	17.6	3.69	2.87	3.85	4.19	3.82	6.10	2.89	Not Performed	<MDL	21.1
Norfluoxetine	n.d.	n.d.	n.d.	n.d.	n.d.	n.d.	n.d.	n.d.	n.d.	n.d.	n.d.	Not Performed	n.d.	n.d.
Paroxetine	n.d.	n.d.	n.d.	n.d.	25.5	n.d.	n.d.	n.d.	n.d.	n.d.	n.d.	Not Performed	n.d.	n.d.
Sertraline	n.d.	n.d.	n.d.	<MDL	n.d.	n.d.	n.d.	n.d.	n.d.	n.d.	n.d.	Not Performed	8.74	21.4
Trazodone	n.d.	n.d.	<MDL	<MDL	n.d.	<MDL	n.d.	n.d.	11.6	7.14	n.d.	Not Performed	35.2	148
Venlafaxine	n.d.	159	17.5	43.0	n.d.	n.d.	<MDL	n.d.	37.2	51.5	n.d.	Not Performed	124	n.d.
Azithromycin	n.d.	n.d.	<MDL	29.6	n.d.	n.d.	n.d.	n.d.	n.d.	n.d.	n.d.	Not Performed	532	41.7
Ciprofloxacin	n.d.	n.d.	n.d.	n.d.	n.d.	n.d.	n.d.	n.d.	n.d.	n.d.	n.d.	Not Performed	n.d.	n.d.
Clarithromycin	n.d.	n.d.	n.d.	8.67	n.d.	n.d.	n.d.	n.d.	n.d.	n.d.	n.d.	Not Performed	187	31.6
Enrofloxacin	n.d.	n.d.	n.d.	n.d.	n.d.	n.d.	n.d.	n.d.	n.d.	n.d.	n.d.	Not Performed	n.d.	n.d.
Ofloxacin	n.d.	n.d.	n.d.	n.d.	n.d.	n.d.	n.d.	n.d.	n.d.	n.d.	n.d.	Not Performed	n.d.	n.d.
Sulfadiazine	n.d.	n.d.	n.d.	n.d.	n.d.	n.d.	n.d.	n.d.	n.d.	n.d.	n.d.	Not Performed	n.d.	n.d.
Sulfadimethoxine	n.d.	n.d.	n.d.	n.d.	n.d.	n.d.	n.d.	n.d.	n.d.	n.d.	n.d.	Not Performed	n.d.	n.d.
Sulfamethazine	n.d.	n.d.	n.d.	n.d.	n.d.	n.d.	n.d.	69.7	123	n.d.	n.d.	Not Performed	n.d.	n.d.
Sulfamethoxazole	n.d.	n.d.	n.d.	43.0	n.d.	n.d.	n.d.	n.d.	n.d.	n.d.	n.d.	Not Performed	n.d.	22.1
Sulfamethoxypyridazine	n.d.	n.d.	n.d.	n.d.	n.d.	n.d.	n.d.	n.d.	n.d.	n.d.	n.d.	Not Performed	n.d.	n.d.
sulfapyridine	n.d.	n.d.	n.d.	n.d.	n.d.	n.d.	n.d.	n.d.	n.d.	n.d.	n.d.	Not Performed	n.d.	n.d.
Trimethoprim	n.d.	n.d.	n.d.	n.d.	n.d.	n.d.	n.d.	n.d.	n.d.	n.d.	n.d.	Not Performed	38.1	80.6
Acetaminophen	95.5	144	51.1	75.4	40.5	138	151	138	140	327	175	Not Performed	93.8	148
Acetylsalicylic acid	n.d.	n.d.	n.d.	n.d.	n.d.	n.d.	n.d.	n.d.	n.d.	n.d.	n.d.	Not Performed	n.d.	n.d.
Carboxyibuprofen	n.d.	n.d.	n.d.	n.d.	n.d.	n.d.	n.d.	n.d.	n.d.	n.d.	n.d.	Not Performed	n.d.	n.d.
Diclofenac	n.d.	38.0	n.d.	n.d.	n.d.	n.d.	n.d.	n.d.	n.d.	n.d.	n.d.	Not Performed	112	848
Hydroxyibuprofen	40.3	n.d.	n.d.	n.d.	n.d.	n.d.	n.d.	n.d.	n.d.	n.d.	n.d.	Not Performed	1295	226
Ibuprofen	<MDL	541	<MDL	<MDL	<MDL	<MDL	827	<MDL	875	277	<MDL	Not Performed	94.7	35.9
Ketoprofen	<MDL	<MDL	41.6	72.2	<MDL	33.3	<MDL	15.4	<MDL	<MDL	19.6	Not Performed	42.4	61.3
Naproxen	n.d.	n.d.	<MDL	202	n.d.	< MDL	n.d.	<MDL	57.0	55.5	n.d.	Not Performed	28.3	130
Nimesulide	n.d.	n.d.	n.d.	n.d.	n.d.	n.d.	n.d.	n.d.	n.d.	n.d.	n.d.	Not Performed	n.d.	n.d.
Salicylic acid	165	172	214	209	129	217	100	139	99.2	123	294	Not Performed	205	125
**SUM**	**340**	**1173**	**419**	**913**	**226**	**454**	**1112**	**408**	**1388**	**931**	**522**		**3117**	**2172**

* Results presented in the study of Paíga, P., Santos, L.H.M.L.M., Ramos, S., Jorge, S., Silva, J.G., and Delerue-Matos, C. Presence of pharmaceuticals in the Lis River (Portugal): Sources, fate and seasonal variation. *Sci Total Environ* **2016,** 573, 164-177.

** Present study.

Table S18. Concentrations of the pharmaceuticals detected during five years in WWTP Effluent E1.

**E1, Effluent**	Concentration (ng/L)													
Pharmaceutical	**Aug2013***	**Sep2013***	**Oct2013***	**Nov2013***	**Jan2014***	**Febr2014***	**March2014***	**April2014***	**May2014***	**Jun2014***	**Jul2014***	**Jun2017**	**Febr2018****	**May2019****
Carbamazepine	Not Performed	Not Performed	98.5	245	83.5	97.2	117	219	211	182	128	Not Performed	1337	834
Citalopram	Not Performed	Not Performed	26.0	61.4	14.9	20.4	n.d.	n.d.	n.d.	n.d.	n.d.	Not Performed	158	157
Diazepam	Not Performed	Not Performed	n.d.	n.d.	n.d.	38.9	n.d.	n.d.	n.d.	n.d.	n.d.	Not Performed	n.d.	n.d.
10,11-Epoxycarbamazepine	Not Performed	Not Performed	n.d.	88.0	n.d.	n.d.	n.d.	n.d.	n.d.	n.d.	n.d.	Not Performed	86.9	57.0
Fluoxetine	Not Performed	Not Performed	12.9	27.5	15.4	11.6	10.5	16.4	33.2	34.0	26.4	Not Performed	13.5	66.8
Norfluoxetine	Not Performed	Not Performed	n.d.	n.d.	n.d.	n.d.	n.d.	n.d.	n.d.	n.d.	n.d.	Not Performed	n.d.	n.d.
Paroxetine	Not Performed	Not Performed	n.d.	n.d.	n.d.	n.d.	n.d.	n.d.	n.d.	n.d.	n.d.	Not Performed	17.8	112
Sertraline	Not Performed	Not Performed	<MDL	n.d.	<MDL	<MDL	n.d.	n.d.	n.d.	<MDL	<MDL	Not Performed	84.9	89.7
Trazodone	Not Performed	Not Performed	7.50	21.7	3.63	6.19	27.8	39.7	69.3	44.9	73.5	Not Performed	111	344
Venlafaxine	Not Performed	Not Performed	91.9	171	63.8	66.1	166	162	201	278	327	Not Performed	370	n.d.
Azithromycin	Not Performed	Not Performed	n.d.	11.4	<MDL	21.5	<MDL	<MDL	<MDL	n.d.	n.d.	Not Performed	4399	210
Ciprofloxacin	Not Performed	Not Performed	96.6	n.d.	n.d.	n.d.	n.d.	99.3	150	146	179	Not Performed	580	257
Clarithromycin	Not Performed	Not Performed	n.d.	70.4	22.3	49.9	n.d.	<MDL	n.d.	n.d.	n.d.	Not Performed	2566	166
Enrofloxacin	Not Performed	Not Performed	n.d.	n.d.	n.d.	n.d.	n.d.	n.d.	n.d.	n.d.	n.d.	Not Performed	n.d.	n.d.
Ofloxacin	Not Performed	Not Performed	n.d.	n.d.	n.d.	n.d.	n.d.	n.d.	n.d.	n.d.	n.d.	Not Performed	1037	110
Sulfadiazine	Not Performed	Not Performed	n.d.	n.d.	n.d.	n.d.	n.d.	n.d.	n.d.	n.d.	n.d.	Not Performed	n.d.	n.d.
Sulfadimethoxine	Not Performed	Not Performed	n.d.	n.d.	n.d.	n.d.	n.d.	n.d.	n.d.	n.d.	n.d.	Not Performed	n.d.	n.d.
Sulfamethazine	Not Performed	Not Performed	n.d.	n.d.	n.d.	n.d.	n.d.	n.d.	n.d.	n.d.	n.d.	Not Performed	n.d.	n.d.
Sulfamethoxazole	Not Performed	Not Performed	n.d.	73.4	n.d.	n.d.	n.d.	n.d.	n.d.	n.d.	n.d.	Not Performed	97.7	n.d.
Sulfamethoxypyridazine	Not Performed	Not Performed	n.d.	n.d.	n.d.	n.d.	n.d.	n.d.	n.d.	n.d.	n.d.	Not Performed	n.d.	n.d.
sulfapyridine	Not Performed	Not Performed	n.d.	n.d.	n.d.	n.d.	n.d.	n.d.	n.d.	n.d.	n.d.	Not Performed	n.d.	n.d.
Trimethoprim	Not Performed	Not Performed	n.d.	59.3	n.d.	n.d.	n.d.	n.d.	n.d.	61.3	n.d.	Not Performed	41.9	215
Acetaminophen	Not Performed	Not Performed	736	2139	45.9	195	484	2463	1906	2299	1723	Not Performed	194	70.8
Acetylsalicylic acid	Not Performed	Not Performed	n.d.	n.d.	n.d.	n.d.	n.d.	n.d.	n.d.	n.d.	n.d.	Not Performed	85	n.d.
Carboxyibuprofen	Not Performed	Not Performed	n.d.	n.d.	n.d.	n.d.	n.d.	n.d.	n.d.	n.d.	n.d.	Not Performed	n.d.	n.d.
Diclofenac	Not Performed	Not Performed	n.d.	n.d.	n.d.	n.d.	165	n.d.	n.d.	123	n.d.	Not Performed	1606	2272
Hydroxyibuprofen	Not Performed	Not Performed	285	359	n.d.	n.d.	n.d.	n.d.	n.d.	n.d.	n.d.	Not Performed	3035	10002
Ibuprofen	Not Performed	Not Performed	517	324	174	757	539	1097	773	<MDL	1086	Not Performed	79.5	2457
Ketoprofen	Not Performed	Not Performed	22.3	55.9	<MDL	<MDL	47.5	28.7	56.7	<MDL	48.0	Not Performed	140	222
Naproxen	Not Performed	Not Performed	<MDL	111	279	<MDL	n.d.	101	n.d.	<MDL	n.d.	Not Performed	534	900
Nimesulide	Not Performed	Not Performed	n.d.	n.d.	n.d.	n.d.	n.d.	n.d.	n.d.	n.d.	n.d.	Not Performed	n.d.	n.d.
Salicylic acid	Not Performed	Not Performed	187	127	126	128	147	296	244	226	249	Not Performed	215	978
**SUM**			**2081**	**3944**	**829**	**1392**	**1703**	**4522**	**3643**	**3394**	**3839**		**16789**	**19518**

* Results presented in the study of Paíga, P., Santos, L.H.M.L.M., Ramos, S., Jorge, S., Silva, J.G., and Delerue-Matos, C. Presence of pharmaceuticals in the Lis River (Portugal): Sources, fate and seasonal variation. *Sci Total Environ* **2016,** 573, 164-177.

** Present study

Table S19. Concentrations of the pharmaceuticals detected during five years in WWTP Effluent E2.

**E2, Effluent**	Concentration (ng/L)													
Pharmaceutical	**Aug2013***	**Sep2013***	**Oct2013***	**Nov2013***	**Jan2014***	**Febr2014***	**March2014***	**April2014***	**May2014***	**Jun2014***	**Jul2014***	**Jun2017****	**Febr2018*****	**May2019*****
Carbamazepine	Not Performed	Not Performed	103	243	62.7	94.9	88.9	139	140	106	2	2118	858	639
Citalopram	Not Performed	Not Performed	n.d.	n.d.	14.3	n.d.	n.d.	n.d.	24.1	29.8	67.4	296	158	153
Diazepam	Not Performed	Not Performed	n.d.	45.5	n.d.	n.d.	n.d.	n.d.	n.d.	n.d.	n.d.	n.d.	n.d.	n.d.
10,11-Epoxycarbamazepine	Not Performed	Not Performed	n.d.	63.4	n.d.	n.d.	n.d.	n.d.	n.d.	n.d.	n.d.	n.d.	n.d.	n.d.
Fluoxetine	Not Performed	Not Performed	13.2	20.4	13.2	13.2	14.0	11.3	14.6	12.6	30.1	115	5.72	63.2
Norfluoxetine	Not Performed	Not Performed	n.d.	n.d.	n.d.	n.d.	n.d.	n.d.	n.d.	n.d.	n.d.	n.d.	n.d.	n.d.
Paroxetine	Not Performed	Not Performed	n.d.	n.d.	n.d.	n.d.	26.5	n.d.	n.d.	n.d.	n.d.	n.d.	n.d.	n.d.
Sertraline	Not Performed	Not Performed	<MDL	<MDL	<MDL	n.d.	<MDL	n.d.	n.d.	n.d.	<MDL	n.d.	50.8	87.8
Trazodone	Not Performed	Not Performed	38.8	85.7	22.6	39.8	8.50	43.4	74.3	62.6	100	317	126	362
Venlafaxine	Not Performed	Not Performed	194	373	89.0	125	86.6	208	227	19	374	968	488	n.d.
Azithromycin	Not Performed	Not Performed	<MDL	<MDL	<MDL	n.d.	<MDL	<MDL	n.d.	n.d.	<MDL	566	4370	34.3
Ciprofloxacin	Not Performed	Not Performed	n.d.	n.d.	n.d.	n.d.	n.d.	n.d.	n.d.	n.d.	n.d.	318	482	n.d.
Clarithromycin	Not Performed	Not Performed	<MDL	<MDL	<MDL	n.d.	n.d.	n.d.	<MDL	n.d.	n.d.	<MDL	1291	17
Enrofloxacin	Not Performed	Not Performed	n.d.	n.d.	n.d.	n.d.	n.d.	n.d.	n.d.	n.d.	n.d.	n.d.	n.d.	n.d.
Ofloxacin	Not Performed	Not Performed	n.d.	n.d.	n.d.	n.d.	n.d.	n.d.	n.d.	n.d.	n.d.	294	382	42
Sulfadiazine	Not Performed	Not Performed	n.d.	n.d.	n.d.	n.d.	n.d.	n.d.	n.d.	n.d.	n.d.	n.d.	n.d.	<MDL
Sulfadimethoxine	Not Performed	Not Performed	n.d.	n.d.	n.d.	n.d.	n.d.	n.d.	n.d.	n.d.	n.d.	n.d.	n.d.	n.d.
Sulfamethazine	Not Performed	Not Performed	n.d.	n.d.	n.d.	n.d.	n.d.	n.d.	n.d.	n.d.	n.d.	n.d.	n.d.	n.d.
Sulfamethoxazole	Not Performed	Not Performed	67.5	n.d.	n.d.	n.d.	n.d.	n.d.	n.d.	n.d.	n.d.	n.d.	67.5	n.d.
Sulfamethoxypyridazine	Not Performed	Not Performed	n.d.	n.d.	n.d.	n.d.	n.d.	n.d.	n.d.	n.d.	n.d.	n.d.	n.d.	n.d.
sulfapyridine	Not Performed	Not Performed	n.d.	n.d.	n.d.	n.d.	n.d.	n.d.	n.d.	n.d.	n.d.	n.d.	n.d.	n.d.
Trimethoprim	Not Performed	Not Performed	n.d.	22.2	<MDL	n.d.	<MDL	n.d.	n.d.	n.d.	n.d.	49	<MDL	236
Acetaminophen	Not Performed	Not Performed	2309	908	2970	2806	313	1941	2454	4910	1347	n.d.	182	494
Acetylsalicylic acid	Not Performed	Not Performed	n.d.	n.d.	n.d.	n.d.	n.d.	n.d.	n.d.	n.d.	n.d.	n.d.	n.d.	n.d.
Carboxyibuprofen	Not Performed	Not Performed	n.d.	n.d.	n.d.	n.d.	n.d.	n.d.	n.d.	n.d.	n.d.	n.d.	2336	n.d.
Diclofenac	Not Performed	Not Performed	287	724	n.d.	277	n.d.	n.d.	370	n.d.	328	3868	1648	2799
Hydroxyibuprofen	Not Performed	Not Performed	780	n.d.	n.d.	n.d.	n.d.	n.d.	n.d.	n.d.	n.d.	589	n.d.	299
Ibuprofen	Not Performed	Not Performed	2397	2578	1497	2273	1419	1665	1936	3140	3304	434	290	163
Ketoprofen	Not Performed	Not Performed	10.2	233	77.7	53.4	<MDL	19.8	<MDL	70.0	17.5	113	222	179
Naproxen	Not Performed	Not Performed	n.d.	<MDL	n.d.	<MDL	221	n.d.	n.d.	74.5	n.d.	n.d.	95.4	492
Nimesulide	Not Performed	Not Performed	n.d.	n.d.	n.d.	n.d.	n.d.	n.d.	n.d.	n.d.	n.d.	n.d.	n.d.	n.d.
Salicylic acid	Not Performed	Not Performed	106	144	118	133	198	186	171	240	246	214	271	142
**SUM**			**6307**	**5441**	**4864**	**5815**	**2375**	**4212**	**5411**	**8842**	**5946**	**10259**	**13323**	**6202**

* Results presented in the study of Paíga, P., Santos, L.H.M.L.M., Ramos, S., Jorge, S., Silva, J.G., and Delerue-Matos, C. Presence of pharmaceuticals in the Lis River (Portugal): Sources, fate and seasonal variation. *Sci Total Environ* **2016,** 573, 164-177.

** Results presented in the study of Paíga, P., Correia, M., Fernandes, M.J., Silva, A., Carvalho, M., Vieira, J., Jorge, S., Silva, J.G., Freire, C., and Delerue-Matos, C. Assessment of 83 pharmaceuticals in WWTP influent and effluent samples by UHPLC-MS/MS: Hourly variation. Sci Total Environ 2019, 648, 582-600.

*** Present study.

Table S20. Concentrations of the pharmaceuticals detected during five years in WWTP Influent I1.

**I1, Influent**	Concentration (ng/L)													
Pharmaceutical	**Aug2013***	**Sep2013***	**Oct2013***	**Nov2013***	**Jan2014***	**Febr2014***	**March2014***	**April2014***	**May2014***	**Jun2014***	**Jul2014***	**Jun2017**	**Febr2018****	**May2019****
Carbamazepine	Not Performed	Not Performed	66.2	111	50.6	226	57.5	114	74.7	89.9	108	Not Performed	1048	733
Citalopram	Not Performed	Not Performed	n.d.	15.1	n.d.	n.d.	n.d.	13.3	20.2	n.d.	n.d.	Not Performed	131	71.6
Diazepam	Not Performed	Not Performed	n.d.	n.d.	n.d.	n.d.	n.d.	n.d.	n.d.	n.d.	n.d.	Not Performed	n.d.	n.d.
10,11-Epoxycarbamazepine	Not Performed	Not Performed	n.d.	n.d.	n.d.	43.2	n.d.	n.d.	n.d.	44.7	n.d.	Not Performed	79.9	n.d.
Fluoxetine	Not Performed	Not Performed	5.21	8.83	7.32	5.69	9.22	10.0	9.50	17.8	9.46	Not Performed	<MDL	82.1
Norfluoxetine	Not Performed	Not Performed	n.d.	n.d.	n.d.	n.d.	n.d.	n.d.	51.2	n.d.	n.d.	Not Performed	n.d.	n.d.
Paroxetine	Not Performed	Not Performed	n.d.	n.d.	n.d.	21.6	22.3	n.d.	n.d.	n.d.	n.d.	Not Performed	n.d.	n.d.
Sertraline	Not Performed	Not Performed	n.d.	n.d.	n.d.	n.d.	n.d.	n.d.	n.d.	<MDL	n.d.	Not Performed	121	224
Trazodone	Not Performed	Not Performed	<MDL	17.7	n.d.	<MDL	<MDL	7.92	18.0	23.6	11.8	Not Performed	29.1	298
Venlafaxine	Not Performed	Not Performed	<MDL	15.4	<MDL	n.d.	n.d.	11.9	10.6	39.4	11.5	Not Performed	367	397
Azithromycin	Not Performed	Not Performed	67.0	8.59	n.d.	n.d.	n.d.	n.d.	n.d.	n.d.	n.d.	Not Performed	652	139
Ciprofloxacin	Not Performed	Not Performed	118.9	n.d.	n.d.	n.d.	n.d.	251	176	226	182	Not Performed	939	264
Clarithromycin	Not Performed	Not Performed	n.d.	n.d.	n.d.	n.d.	<MDL	<MDL	n.d.	n.d.	n.d.	Not Performed	2214	158
Enrofloxacin	Not Performed	Not Performed	n.d.	n.d.	n.d.	n.d.	n.d.	n.d.	n.d.	n.d.	n.d.	Not Performed	n.d.	n.d.
Ofloxacin	Not Performed	Not Performed	n.d.	n.d.	n.d.	n.d.	n.d.	n.d.	n.d.	n.d.	n.d.	Not Performed	1071	<MDL
Sulfadiazine	Not Performed	Not Performed	n.d.	<MDL	n.d.	n.d.	n.d.	n.d.	n.d.	n.d.	n.d.	Not Performed	n.d.	n.d.
Sulfadimethoxine	Not Performed	Not Performed	n.d.	n.d.	n.d.	n.d.	n.d.	n.d.	n.d.	n.d.	n.d.	Not Performed	n.d.	n.d.
Sulfamethazine	Not Performed	Not Performed	n.d.	n.d.	n.d.	n.d.	n.d.	n.d.	n.d.	n.d.	n.d.	Not Performed	n.d.	n.d.
Sulfamethoxazole	Not Performed	Not Performed	n.d.	224	n.d.	50.6	n.d.	164	n.d.	333	343	Not Performed	945	179
Sulfamethoxypyridazine	Not Performed	Not Performed	n.d.	n.d.	n.d.	n.d.	n.d.	n.d.	n.d.	n.d.	n.d.	Not Performed	n.d.	n.d.
sulfapyridine	Not Performed	Not Performed	n.d.	n.d.	n.d.	n.d.	n.d.	43.4	40.8	95.5	256	Not Performed	696	245
Trimethoprim	Not Performed	Not Performed	n.d.	n.d.	n.d.	n.d.	n.d.	n.d.	n.d.	n.d.	n.d.	Not Performed	<MDL	236
Acetaminophen	Not Performed	Not Performed	30030	615135	10568	2024	7815	182072	159225	180459	204232	Not Performed	96770	30949
Acetylsalicylic acid	Not Performed	Not Performed	n.d.	n.d.	n.d.	n.d.	n.d.	n.d.	n.d.	n.d.	n.d.	Not Performed	99.5	n.d.
Carboxyibuprofen	Not Performed	Not Performed	41554	120365	37120	7215	19300	85960	56165	76409	74383	Not Performed	392986	14282
Diclofenac	Not Performed	Not Performed	n.d.	n.d.	n.d.	n.d.	971.9	n.d.	n.d.	n.d.	n.d.	Not Performed	1939	2178
Hydroxyibuprofen	Not Performed	Not Performed	190	198.	<MDL	n.d.	n.d.	196	115	n.d.	n.d.	Not Performed	169633	11364
Ibuprofen	Not Performed	Not Performed	4389	14125	7628	4069	3877	11755	6394	17511	19118	Not Performed	5862	4655
Ketoprofen	Not Performed	Not Performed	<MDL	<MDL	<MDL	<MDL	<MDL	<MDL	<MDL	<MDL	<MDL	Not Performed	66.3	384
Naproxen	Not Performed	Not Performed	2079	533	3245	1417	1091	459	236	812	791	Not Performed	2225	3126
Nimesulide	Not Performed	Not Performed	n.d.	n.d.	n.d.	n.d.	n.d.	n.d.	n.d.	n.d.	n.d.	Not Performed	n.d.	n.d.
Salicylic acid	Not Performed	Not Performed	6332	33536	1610	1174	1186	9030	13514	16086	10386	Not Performed	15719	4612
**SUM**			**84832**	**784292**	**60228**	**16246**	**34330**	**290088**	**236050**	**292146**	**309831**		**693594**	**74574**

* Results presented in the study of Paíga, P., Santos, L.H.M.L.M., Ramos, S., Jorge, S., Silva, J.G., and Delerue-Matos, C. Presence of pharmaceuticals in the Lis River (Portugal): Sources, fate and seasonal variation. *Sci Total Environ* **2016,** 573, 164-177.

** Present study.

Table S21. Concentrations of the pharmaceuticals detected during five years in WWTP Influent I2.

**I2, Influent**	Concentration (ng/L)													
Pharmaceutical	**Aug2013***	**Sep2013***	**Oct2013***	**Nov2013***	**Jan2014***	**Febr2014***	**March2014***	**April2014***	**May2014***	**Jun2014***	**Jul2014***	**Jun2017****	**Febr2018*****	**May2019*****
Carbamazepine	Not Performed	Not Performed	101	107	47.0	67.1	65.5	70.6	116	120	101	1639	652	633
Citalopram	Not Performed	Not Performed	13.4	35.7	n.d.	n.d.	n.d.	n.d.	19.3	28.1	n.d.	299	144	81
Diazepam	Not Performed	Not Performed	n.d.	n.d.	n.d.	n.d.	n.d.	n.d.	n.d.	n.d.	n.d.	n.d.	73	n.d.
10,11-Epoxycarbamazepine	Not Performed	Not Performed	n.d.	n.d.	n.d.	n.d.	n.d.	n.d.	n.d.	n.d.	n.d.	n.d.	56	n.d.
Fluoxetine	Not Performed	Not Performed	9.21	9.20	5.20	8.24	8.14	8.69	9.39	8.32	8.03	156	<MDL	81.8
Norfluoxetine	Not Performed	Not Performed	n.d.	45.2	n.d.	n.d.	n.d.	n.d.	n.d.	n.d.	n.d.	n.d.	n.d.	n.d.
Paroxetine	Not Performed	Not Performed	n.d.	n.d.	n.d.	n.d.	23.6	n.d.	n.d.	n.d.	n.d.	n.d.	n.d.	n.d.
Sertraline	Not Performed	Not Performed	n.d.	n.d.	n.d.	n.d.	n.d.	n.d.	n.d.	n.d.	n.d.	n.d.	106	171
Trazodone	Not Performed	Not Performed	n.d.	n.d.	n.d.	5.13	n.d.	13.7	38.3	31.6	33.6	465	115	414
Venlafaxine	Not Performed	Not Performed	50.0	65.2	<MDL	n.d.	27.2	24.7	49.2	66.7	53.5	550	408	n.d.
Azithromycin	Not Performed	Not Performed	n.d.	n.d.	n.d.	n.d.	n.d.	n.d.	n.d.	<MDL	n.d.	804	1096	15.1
Ciprofloxacin	Not Performed	Not Performed	174	246	n.d.	n.d.	n.d.	n.d.	n.d.	n.d.	n.d.	897	1814	378
Clarithromycin	Not Performed	Not Performed	n.d.	48.2	n.d.	n.d.	n.d.	n.d.	n.d.	n.d.	n.d.	<MDL	1690	12.5
Enrofloxacin	Not Performed	Not Performed	n.d.	n.d.	n.d.	n.d.	n.d.	n.d.	n.d.	n.d.	n.d.	n.d.	n.d.	n.d.
Ofloxacin	Not Performed	Not Performed	n.d.	n.d.	n.d.	n.d.	n.d.	n.d.	n.d.	n.d.	n.d.	<MDL	954	39.9
Sulfadiazine	Not Performed	Not Performed	n.d.	n.d.	n.d.	n.d.	n.d.	n.d.	n.d.	n.d.	n.d.	n.d.	n.d.	20.6
Sulfadimethoxine	Not Performed	Not Performed	n.d.	n.d.	n.d.	n.d.	n.d.	n.d.	n.d.	n.d.	n.d.	n.d.	n.d.	n.d.
Sulfamethazine	Not Performed	Not Performed	n.d.	n.d.	n.d.	n.d.	n.d.	n.d.	n.d.	n.d.	n.d.	n.d.	n.d.	n.d.
Sulfamethoxazole	Not Performed	Not Performed	n.d.	173	n.d.	140	n.d.	n.d.	243	203	290	1200	917	291
Sulfamethoxypyridazine	Not Performed	Not Performed	n.d.	n.d.	n.d.	n.d.	n.d.	n.d.	n.d.	n.d.	n.d.	n.d.	n.d.	n.d.
sulfapyridine	Not Performed	Not Performed	75.9	n.d.	n.d.	n.d.	n.d.	n.d.	n.d.	162	228	985	955	353
Trimethoprim	Not Performed	Not Performed	n.d.	n.d.	n.d.	n.d.	n.d.	n.d.	n.d.	n.d.	n.d.	n.d.	<MDL	349
Acetaminophen	Not Performed	Not Performed	119560	215182	41983	33493	32610	287801	125873	151233	77633	1365	82702	31965
Acetylsalicylic acid	Not Performed	Not Performed	n.d.	n.d.	n.d.	n.d.	n.d.	n.d.	n.d.	n.d.	n.d.	n.d.	115	n.d.
Carboxyibuprofen	Not Performed	Not Performed	n.d.	n.d.	n.d.	n.d.	n.d.	n.d.	n.d.	n.d.	n.d.	n.d.	639403	21480
Diclofenac	Not Performed	Not Performed	n.d.	n.d.	n.d.	n.d.	n.d.	n.d.	n.d.	n.d.	n.d.	998	3316	3372
Hydroxyibuprofen	Not Performed	Not Performed	n.d.	n.d.	n.d.	n.d.	n.d.	103	180	334	178	5675	283651	11273
Ibuprofen	Not Performed	Not Performed	18792	24505	12557	15476	17962	14095	14052	1636	21039	842	12490	8229
Ketoprofen	Not Performed	Not Performed	<MDL	<MDL	147	116	121	<MDL	<MDL	<MDL	<MDL	<MDL	564	552
Naproxen	Not Performed	Not Performed	500	<MDL	1133	536	1058	234	211	373	236	57.0	3004	1696
Nimesulide	Not Performed	Not Performed	n.d.	n.d.	n.d.	n.d.	n.d.	n.d.	n.d.	n.d.	n.d.	n.d.	n.d.	n.d.
Salicylic acid	Not Performed	Not Performed	27461	61259	1616	1241	1344	7776	20722	3421	17421	2198	29012	32733
**SUM**			**166737**	**301676**	**57488**	**51083**	**53220**	**310127**	**161513**	**172342**	**117219**	**18130**	**1063237**	**114140**

* Results presented in the study of Paíga, P., Santos, L.H.M.L.M., Ramos, S., Jorge, S., Silva, J.G., and Delerue-Matos, C. Presence of pharmaceuticals in the Lis River (Portugal): Sources, fate and seasonal variation. *Sci Total Environ* **2016,** 573, 164-177.

** Results presented in the study of Paíga, P., Correia, M., Fernandes, M.J., Silva, A., Carvalho, M., Vieira, J., Jorge, S., Silva, J.G., Freire, C., and Delerue-Matos, C. Assessment of 83 pharmaceuticals in WWTP influent and effluent samples by UHPLC-MS/MS: Hourly variation. Sci Total Environ 2019, 648, 582-600.

*** Present study.

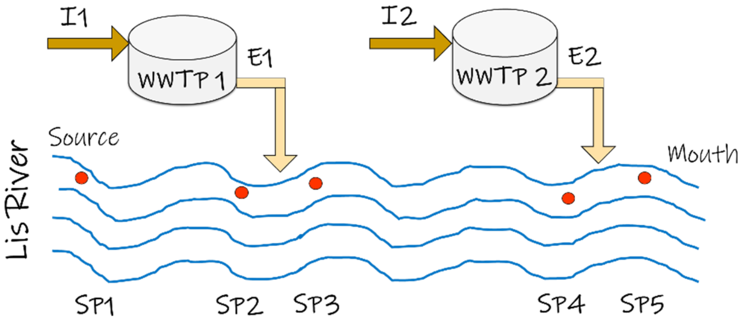

Figure S1. Scheme of sampling points in the Lis River (SP1 to SP5) and WWTP influents (WWTP I1 and WWTP I2) and effluents (WWTP E1 and WWTP E2).

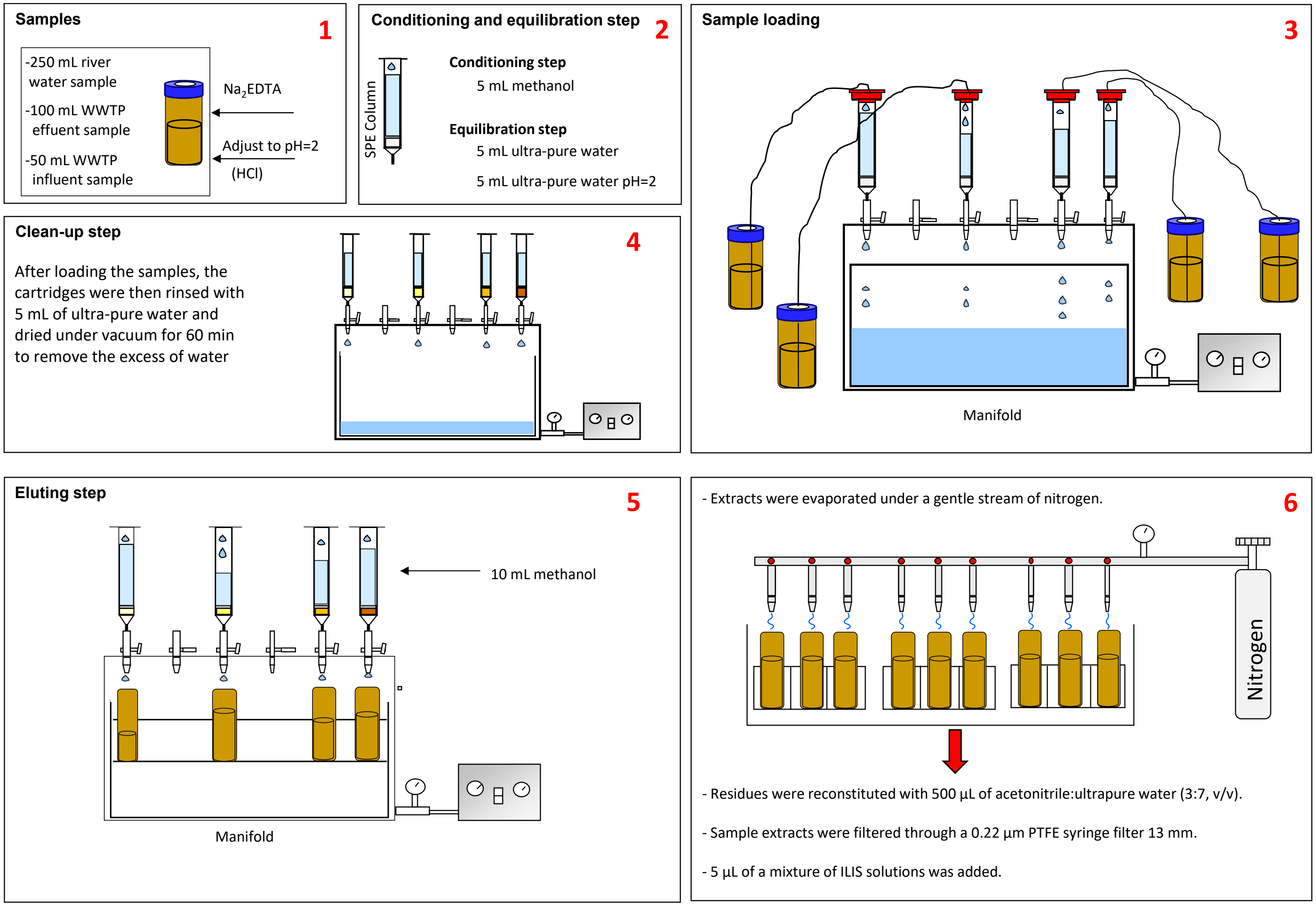

Figure S2. Solid phase extraction procedure used for the extraction of the studied compounds in river water and in wastewaters (influent and effluent) samples.

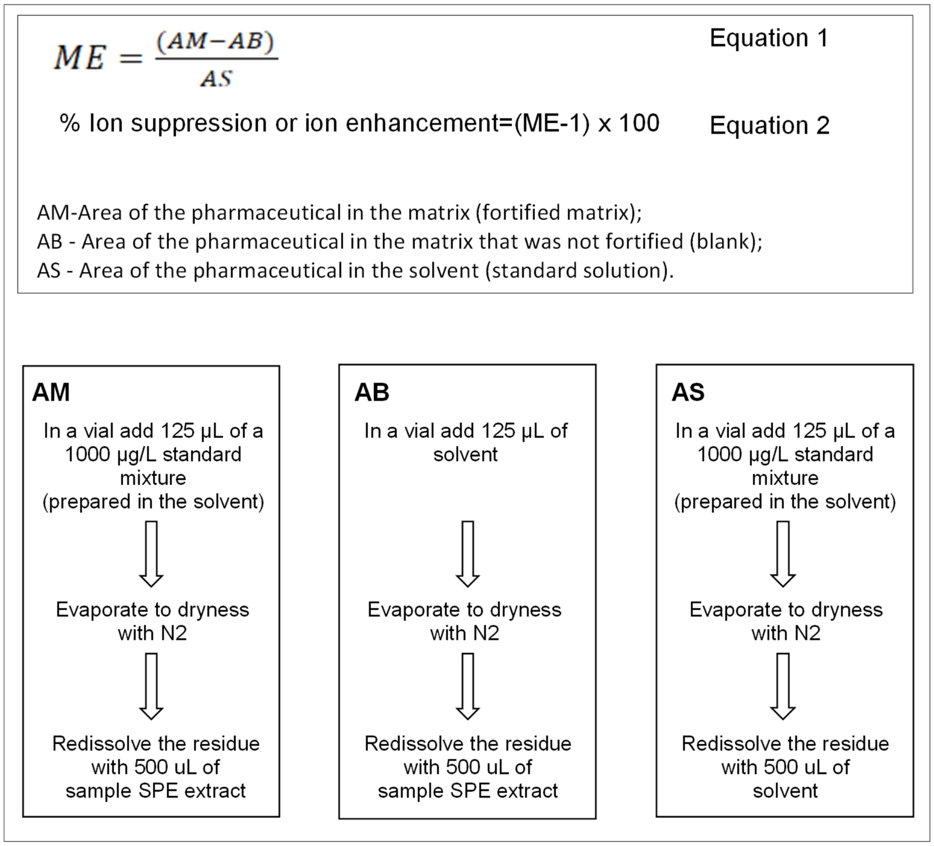

Figure S3. Procedures used for the matrix effect determination in the analyzed matrices.

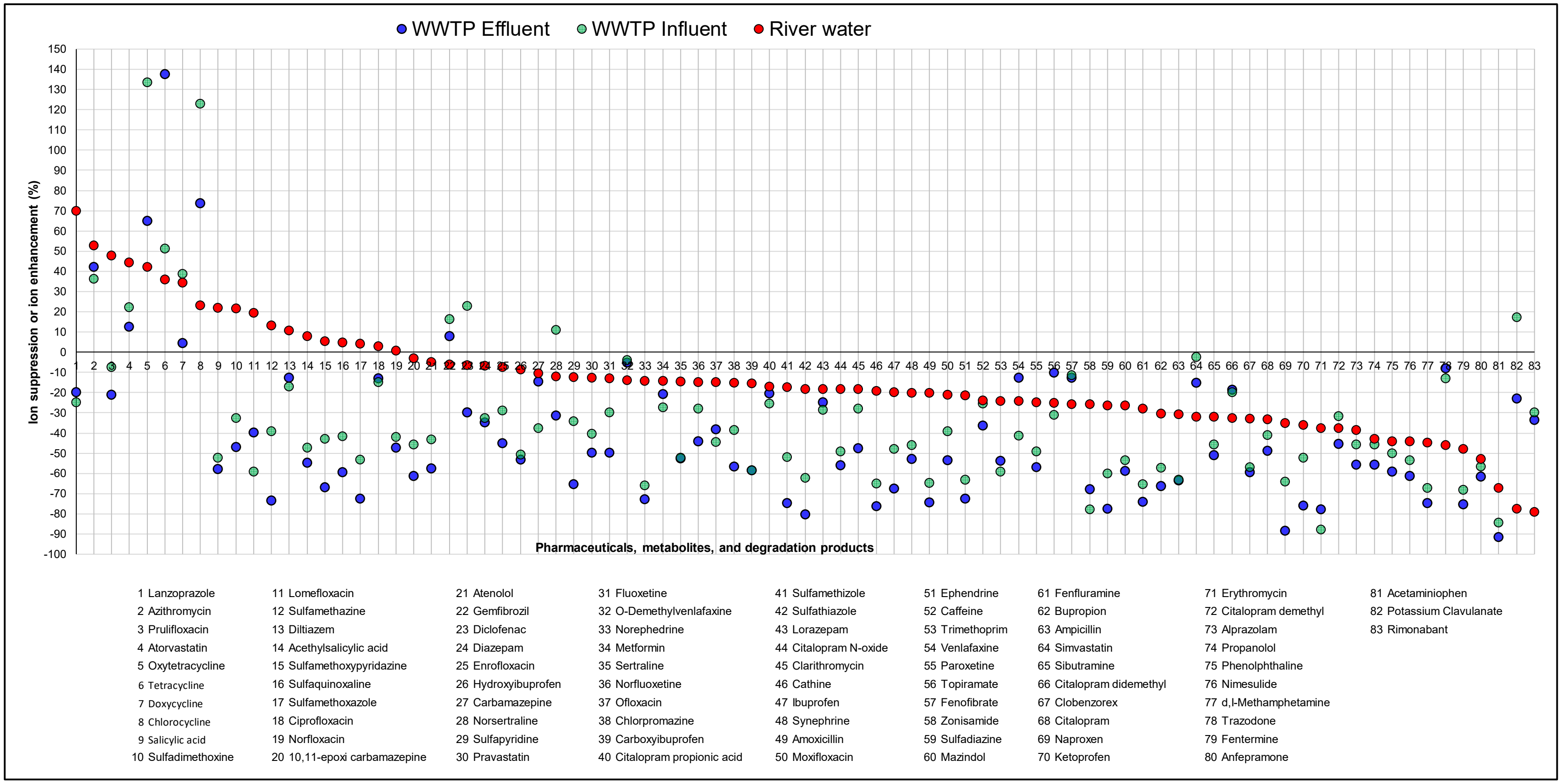

Note: Pharmaceuticals, Metabolites, and degradation products were organized from the highest to the lowest ion enhancement signal and then to the lowest to the highest ion suppression signal for the river water matrix.

Figure S4. Matrix effect in the river water and WWTP effluent and influent matrices.

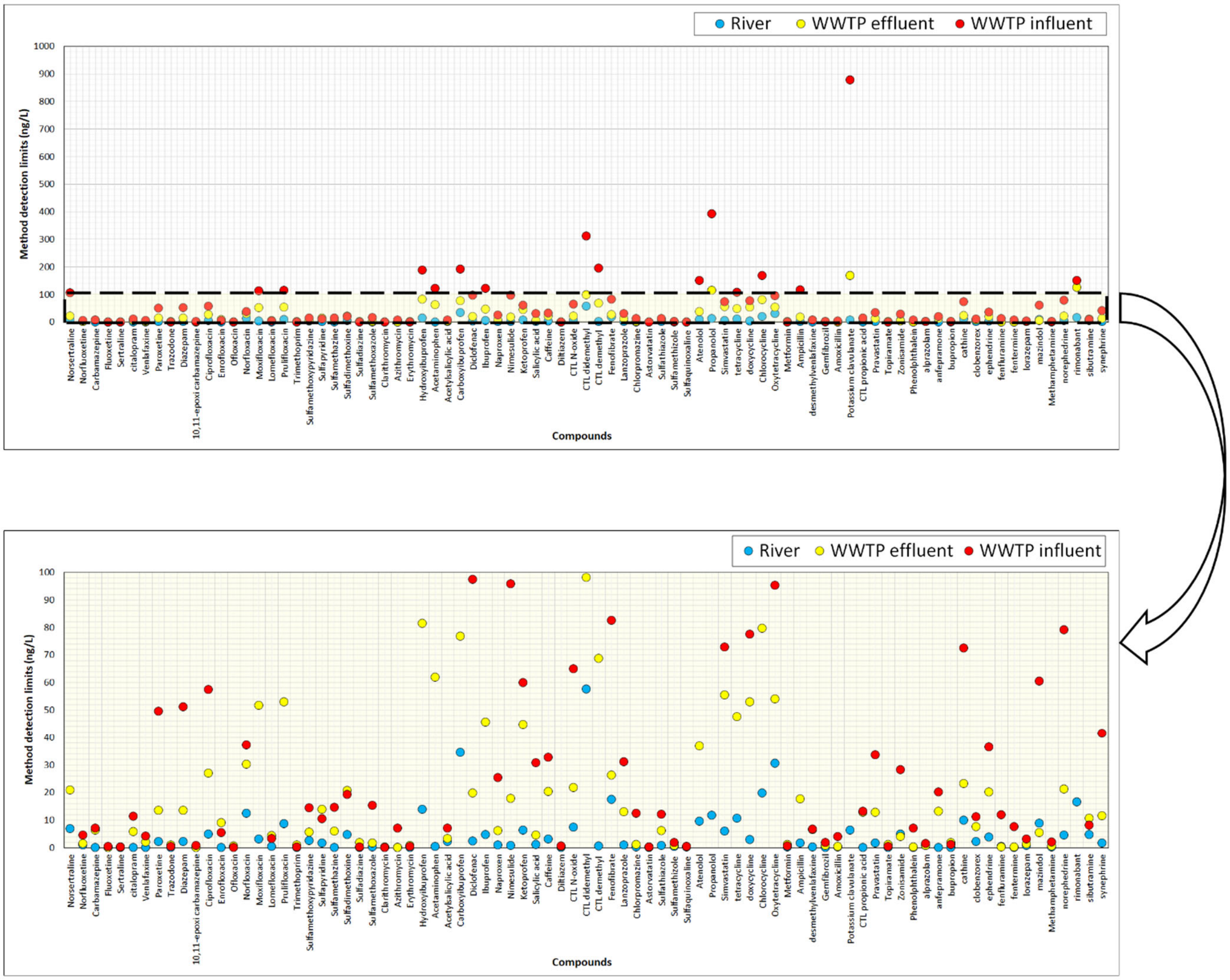

Figure S5. Method detection limits (MDL) for the studied compound for river water and WWTP effluent and influent matrices.

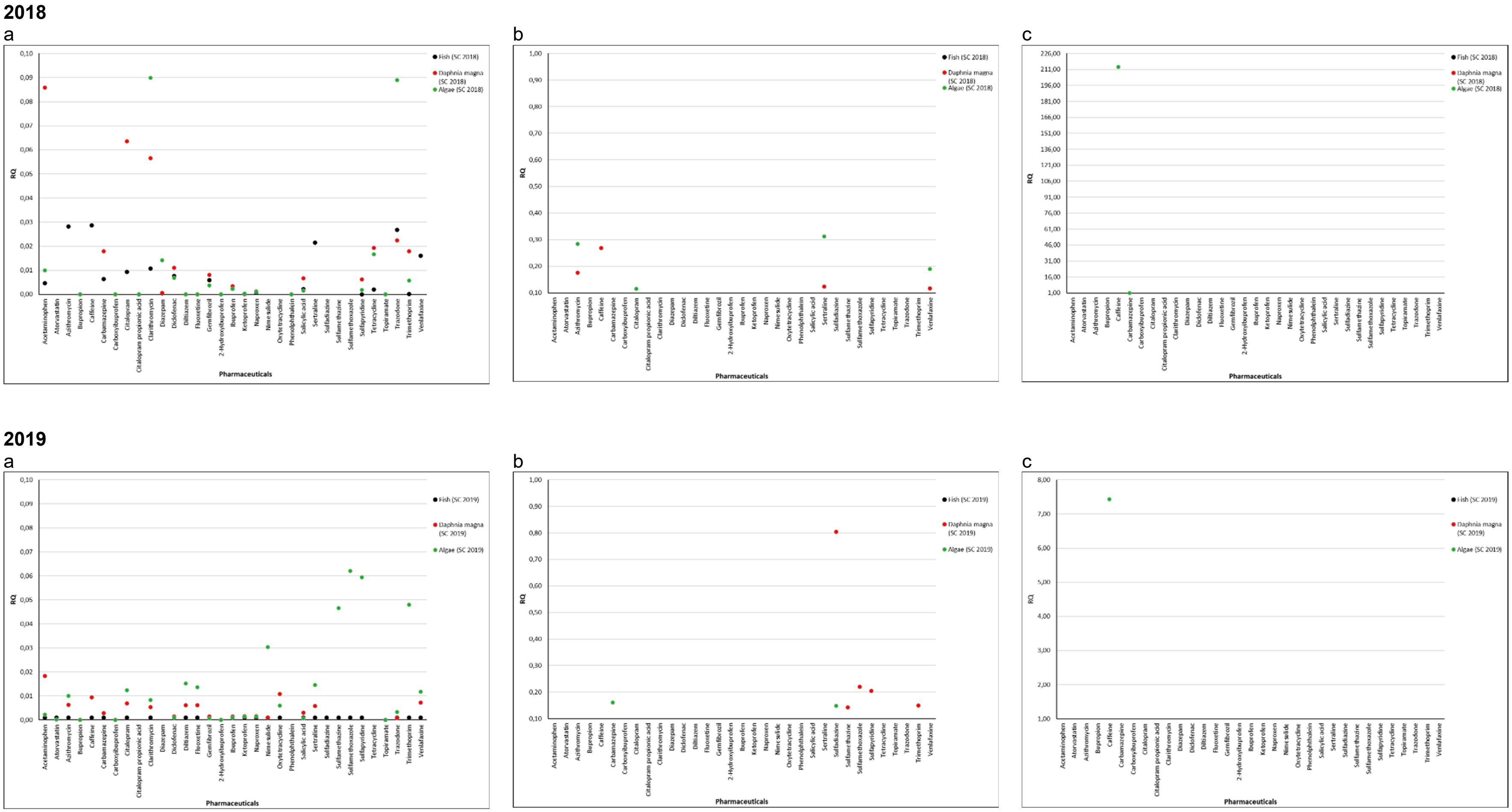

Figure S6. Risk Quotient obtained in each trophic level: (a) lower risk (RQ<0.1), (b) moderate risk (0.1<RQ<1), and (c) high risk (RQ>1).
